# USP24-i-101 targeting of USP24 activates autophagy to inhibit drug resistance acquired during cancer therapy

**DOI:** 10.1038/s41418-024-01277-7

**Published:** 2024-03-15

**Authors:** Ming-Jer Young, Shao-An Wang, Yung-Ching Chen, Chia-Yu Liu, Kai-Cheng Hsu, Sin-Wei Tang, Yau-Lin Tseng, Yi-Ching Wang, Shih-Min Lin, Jan-Jong Hung

**Affiliations:** 1 Department of Biotechnology and Bioindustry Sciences, National Cheng Kung University, Tainan, Taiwan; 2 School of Respiratory Therapy, College of Medicine, Taipei Medical University, Taipei, Taiwan; 3 Graduate Institute of Cancer Biology and Drug Discovery, College of Medical Science and Technology, Taipei Medical University, Taipei, Taiwan; 4 National Tainan First Senior High School, Tainan, Taiwan; 5 Division of Thoracic Surgery, Department of Surgery, College of Medicine National Cheng Kung University, Tainan, Taiwan; 6 Institute of Pharmacology, National Cheng Kung University, Tainan, Taiwan

**Keywords:** Lung cancer, Autophagy

## Abstract

Drug resistance in cancer therapy is the major reason for poor prognosis. Addressing this clinically unmet issue is important and urgent. In this study, we found that targeting USP24 by the specific USP24 inhibitors, USP24-i and its analogues, dramatically activated autophagy in the interphase and mitotic periods of lung cancer cells by inhibiting E2F4 and TRAF6, respectively. USP24 functional knockout, *USP24*^*C1695A*^, or targeting USP24 by USP24-i-101 inhibited drug resistance and activated autophagy in gefitinib-induced drug-resistant mice with doxycycline-induced *EGFR*^*L858R*^ lung cancer, but this effect was abolished after inhibition of autophagy, indicating that targeting USP24-mediated induction of autophagy is required for inhibition of drug resistance. Genomic instability and PD-L1 levels were increased in drug resistant lung cancer cells and were inhibited by USP24-i-101 treatment or knockdown of USP24. In addition, inhibition of autophagy by bafilomycin-A1 significantly abolished the effect of USP24-i-101 on maintaining genomic integrity, decreasing PD-L1 and inhibiting drug resistance acquired in chemotherapy or targeted therapy. In summary, an increase in the expression of USP24 in cancer cells is beneficial for the induction of drug resistance and targeting USP24 by USP24-i-101 optimized from USP24-i inhibits drug resistance acquired during cancer therapy by increasing PD-L1 protein degradation and genomic stability in an autophagy induction-dependent manner.

## Introduction

Drug resistance is induced by drug treatment in various diseases, such as bacterial infection and cancer, decreasing the effectiveness of the drug. Although many related studies have attempted to solve this issue, drug resistance remains a major problem. What are the challenges regarding cancer drug resistance? One major challenge is that drug resistance is multifactorial. The second challenge is that drug resistance is heterogeneous. The final challenge is that drug resistance is prone to undersampling in translational investigation [1]. Many factors are involved in drug resistance, including increased drug efflux, decreased drug uptake, altered cell cycle checkpoints, the induction of emergency response genes, apoptotic inhibition, drug compartmentalization and altered drug targets. Two major classes of drug resistance-associated membrane proteins have been identified: the ATP-binding cassette (ABC) transporter superfamily and solute carrier transporters [2]. All of these pathways are regulated by gene mutation and DNA damage repair pathways. Therefore, genome stabilization can decrease the rate of mutation and prevent the dysregulation of genes, potentially inhibiting drug resistance during cancer therapy. Our previous studies indicated that knockdown of USP24 is involved in the protein degradation of P-gp and ABCG2 in a proteasome-dependent and lysosome-dependent manner, respectively [3].

Deubiquitinases (DUBs) are specific enzymes that regulate multiple cellular functions by modulating ubiquitination. Ubiquitin-specific peptidases (USPs) belong to the DUB superfamily, which has been related to many human diseases, including cancer progression [4, 5]. More than 50 USPs have been identified, and most of these enzymes can reverse the polyubiquitination or monoubiquitination of target proteins. A malfunction in the ubiquitin system can either enhance the effect of oncogenes or reduce the activity of tumor suppressor genes, and therefore, this system has been implicated in the tumorigenesis of various cancers [6, 7]. In our previous study, we demonstrated that USP24 expression was upregulated in most late-stage lung cancer patients due to increased mRNA stability caused by single nucleotide polymorphisms (SNPs) or RNA editing [8]. We also observed that upregulation of USP24 increased the stability of MDM2, which is the E3-ligase of the methyltransferase Suv39h1, thereby increasing Suv39h1 degradation. The downregulation of Suv39h1 released downstream genes from inhibition, leading to the expression of metastasis-related genes, such as those encoding CCL5 and ADAM10 [8].

Autophagy is a lysosome-dependent degradation pathway that promotes cell homeostasis in response to various stress conditions, such as DNA damage, oxidative stress and starvation. Autophagy-related (atg) genes regulated by several kinases, such as mTOR and MAPK, are involved in autophagic progression [9, 10]. Three types of autophagy, macroautophagy, microautophagy and chaperone-mediated autophagy, use distinct mechanisms to degrade their client proteins in a lysosome-dependent manner [11]. Increasing evidence supports that autophagy is involved in various diseases, including cancer progression, diabetes and neuron degenerative diseases [12–15]. In cancer progression, the role of autophagy is very different in different cancer types and stages. Some studies have shown that autophagy positively regulates cancer progression, including proliferation, malignancy and drug resistance, and some other studies have shown negative regulation [16, 17]. Most of the studies on the role of autophagy in diabetes and neurodegenerative diseases are consistent. Inhibition of autophagy is a major cause of diabetes and neurodegenerative diseases [18–20]. Ubiquitination of proteins is involved in protein degradation in a proteasome-dependent manner. Proteins modified by ubiquitin can also be degraded *via* an autophagic pathway [21, 22]. Two ubiquitin-like conjugations found during autophagy, ATG12 and ATG8/LC3, are essential for the induction of autophagy [23]. ATG12 can be conjugated with ATG5 by ATG7, and LC3 can be conjugated to the membrane to form LC3-II [24, 25]. Several E3-ligases and DUBs have been found to be involved in autophagic progression [26]. Our recent results indicated that USP24 knockout or USP24-i treatment induced autophagy in vivo and in vitro to inhibit the drug resistance of cancers acquired from Taxol or gefitinib treatment.

## Material and methods

### Cell culture and transfection

Human lung adenocarcinoma epithelial cell lines, A549 was obtained from the American Type Culture Collection (ATCC). The human lung cancer cell lines PC9 and HCC827 kindly provided by Dr. Shang-Yin Wu (National Cheng Kung University, Tainan, Taiwan).

The breast cancer cell lines, MCF7 and MDAMB231 kindly provided by Dr. Ju-Ming Wang (National Cheng Kung University, Tainan, Taiwan). These cells were cultured with RPMI 1640 medium (Corning, Manassas, VA, USA) containing 10% fetal bovine serum (FBS, Thermal Fisher), 100 µg per ml streptomycin and 100 U per ml penicillin G sodium (Thermal Fisher). Taxol-resistant A549 (A549-T24), gefitinib-resistant PC9 (PC9-GR), and gefitinib-resistant HCC827 (HCC827-GR were maintained in the same culture medium containing Taxol and gefitinib (Sigma-Aldrich, St. Louis, MO, USA), respectively. Human hepatoma cell lines, HepG2 and Huh7, obtained from the laboratory of Professor Wen-Ya Huang in NCKU of Taiwan were cultured with DMEM medium (Corning, Manassas, VA, USA). All cells were incubated at 37 °C with 5% CO2. For transfecting plasmid, Polyjet (SignaGen) was used according to manufacturer’s instructions. All cell lines were tested for mycoplasma contamination and results were negative.

### Lentivirus knockdown system

Scramble knockdown (TRCN0000072246; CAAATCACAGAATCGTCGTAT) and USP24 knockdown lentivirus (TRCN0000245779; CTCTCGTATGTAACGTATTTG) were generated form RNAi core facility of Academia Sinica (Taipei, Taiwan). Cells were seeded in 6-well plates and incubated for 16 h, and then treated with 1 ml RPMI medium containing 10 µg Polybrene (Millipore) and lentivirus with 5 m.o.i. After 24 h of infection, medium containing lentivirus was replaced with fresh medium and maintained for another 72 h.

### Traffic light assay

A549-T24 cells were cultured on coverslips and upon transfecting cells with the 1 μg mRFP-EGFP-LC3 plasmid for 24 h, and then were exposed to distinct treatments of 10 μM Rapamycin, 10 nM Bafilomycin A1, and 5 μM USP24-I for an additional 24 h. The cells were fixed with 4% formaldehyde, and then the signal of EGFP and mRFP was measured with a Laser confocal microscope (FV1000, Olympus, Japan).

### Cell viability assay

Cells seeded in 96-well plates (1 × 10^4^ cells/well) were treated with 5 μM USP24-i-101, 24 nM Taxol (paclitaxel, Selleckchem, S1150), 10 nM Baf-A1 (Bafilomycin A1, Abcam, ab120497), and 5 μM CQ (Chloroquine, MedChem Express, HY-17589A) for 24 h. The cell viability was assessed using the PrestoBlue cell viability kit following the manufacturer’s instructions (Thermo Fisher Scientific).

### in vitro deubiquitination assay

PC9-GR cells were treated with 10 μM MG132 (Sigma-Aldrich) for 12 h. Cell lysates were harvested for immunoprecipitation assay (IP) with anti-PD-L1 antibodies (1:100) for 4 h, and then incubated with 140 μl protein A agarose (Millipore) for 1 h. After three times washing, the IP samples were mixed with human recombinant USP24 protein (50 µg per ml) (OriGene) in the deubiquitination buffer (50 mM Tris PH 8.0, 10 mM DTT and 5 µM MG132) for 2 h in 37 °C. The reaction was stopped by adding 140 μl sample buffer. The signal of ubiquitinated proteins were measured by western blotting.

### Protein stability assay

Cells were infected with scramble or USP24 shRNA expressing lentivirus (m.o.i. = 5) for 4 days, and then treated with 100 µg/ml cycloheximide (Sigma-Aldrich, St. Louis, Missouri, USA). Cells were harvested with sample buffer, and protein levels were measured by Western blot. The protein levels were quantified by using ImageJ 8.0 software.

### Immunohistochemistry

The human investigations were systematically conducted in strict compliance with prevailing guidelines and regulatory protocols. The study using human specimens was provided by National Cheng Kung University Hospital (NCKUH), under the approval of the Institutional Review Board (IRB) of NCKUH (IRB No.: B-ER-108-368). Human and animal specimens were incubated in 10% formaldehyde for 24 h for fixation, dehydration, and embedded in paraffin. Hematoxylin and eosin were used for staining sections (5 mm). For immunohistochemistry, paraffin-embedded sections were incubated in xylene for dewaxing and a graded series of ethanol for dehydration. Sections were incubated in PBS with 0.3% hydrogen peroxide for 30 min to block endogenous peroxidase, and then incubated in PBS with 1% bovine serum albumin for blocking. The anti-USP24 (1:200) (13126-1-AP, Proteintech, USA), anti-PD-L1 (1:200) (#13684, Cell Signaling technology, Danvers, MA, USA), anti-LC3-II (1:200) (#3868, Cell Signaling technology, Danvers, MA, USA) and anti-CD3 (1:200) antibodies (A700-174, Bethyl Laboratories, Inc., Montgomery, TX, USA) were used to cover sections for 1 h at room temperature, and the Vectastain ABC kit (Vector Laboratories, Burlingame, CA, USA) was used for visualizing the immunoreactivity. Sections were photographed by Olympus BX-51 microscope (Olympus, Melville, NY, USA).

### Immunofluorescent analysis

Cells seeded in 6-well plates with cover slips inside were infected with scramble and shUSP24-knockdown lentivirus (m.o.i. = 5) for 48 h. The cells in coverslips were fixed with 4% paraformaldehyde at 4 °C for 15 min. After fixation, coverslips were washed with PBS, and incubated with 0.2% Triton X-100 in PBS for 5 min at room temperature, then were blocked with 1% Bovine serum albumin (BSA) for 1 h, and stained with anti-GFP (1:200) (SC-9996, Santa Cruz Biotechnology, Santa Cruz, CA, USA), anti-LC3-II (1:200) (#3868, Cell Signaling technology, Danvers, MA, USA) for 16 h at 4 °C. After washing with PBS, cells were stained with Alexa Fluor® 488 or 568 (Invitrogen, Carlsbad, CA, USA) for 1 h at room temperature and mounted with 90% glycerol containing DAPI (Invitrogen, Carlsbad, CA, USA). The number of stained cells were examined by fluorescence microscopy (Olympus, Tokyo, Japan.). ImageJ (Bethesda, Maryland, USA.) was used to perform the statistical analysis.

### Animal cares, lung cancer animal model, and drug resistant lung cancer animal model

The experiments related to animals were approved by the Institutional Animal Care and Use Committee (IACUC: 109110) at National Cheng Kung University (NCKU). These transgenic mice were generated at National Laboratory Animal Center (NLAC, Taiwan, Tainan). After breeding, two-month-old transgenic mice were used to study lung cancer development. Caging provided suitable space and accommodated appropriate population densities that allowed animals sufficient freedom of movement. To provide amounts of food that must be for transgenic mice to normal growth, and maintenance of normal body weight. These transgenic mice were accessed to fresh and uncontaminated drinking water. Transgenic mice were also observed and cared at least for two to three times per week. All methods involving animals were performed in accordance with the relevant guidelines and regulations. *USP24* is localized on mice chromosome 4. By using CRISPR/Cas9 system to target *USP24* point mutation, C1695A, to construct C57BL/6J-NarI-*USP24*^*C1695A*^ mice. The used guide sequence is 5′-ACGGTGGCGCCACTGCTTACATGAATGCAGTGTTCCAGCAGC-3′ to add one another restriction site, NarI, delete one restriction site, BbsI, and create one mutation residue from cysteine to alanine (C1695A) (Supplementary Fig. 1A). *TetO-EGFR*^*L858R*^ transgenic mice were obtained from the laboratory of Professor Ming-Derg Lai in NCKU of Taiwan. The Scgb1a1-rtTA transgenic mice express the rtTA (reverse tetracycline trans-activator) protein regulated by the promoter of Scgb1a1, which is a specific promoter of lung. The *TetO-EGFR*^*L858R*^ transgenic mice expressed activated EGFR (EGFR^L858R^) regulated by the promoter contained TRE (tetracycline-responsive promoter element). After mating, the genomic DNA was isolated from the tail of mice, *EGFR*^*L858R*^*Usp24*^*C1695A*^ transgenic mice, and then genotyped by PCR using following primer: *rtTA* forward: 5′-AAAATCTTGCCAGCTTTCCCC-3′, reverse: 5′-ACTGCCCATTGCCCAAACAC-3′, *EGFR*^*L858R*^ forward: 5′-ACTGTCCAGCCCACCTGTGT-3′, reverse: 5′-GCCTGCGACGGCGGCACTCTGC-3′ and *Usp24*^*C1695A*^ forward: 5′-CGTACCAGCTCCTAACACAG-3′, reverse: 5′-GCCTGCTACTCCACAAGTTG-3′. Usp24^C1695A^ PCR product used NarI (New England Biolabs) at 37 °C for 1 h. EGFR^L858R^ was expressed by adding doxycycline (0.5 g/l) to the drinking water. After 1.5 months, the signal of lung tumor was measured by micro-CT (SkyScan-1276), and then treatment with gefitinib (15 mg/kg, i.p.), two times per week until to 31^th^ week.

### Western blotting

Cell extracts were harvested by sample buffer and analyzed by electrophoresis. Proteins were transferred to polyvinylidene difluoride (PVDF, Millipore, Bedford, MA, USA) membrane and TBST buffer (10 mM Tris-HCl, pH 8.0, 150 mM NaCl and 0.05% Tween 20) containing 5% nonfat milk was used for blocking. The anti-LC3-II (1:1000) (#3868, Cell Signaling technology, Danvers, MA, USA), anti-ULK1 (1:1000) (#8054, Cell Signaling technology, Danvers, MA, USA), anti-p62 (1:1000) (GTX100685, Genetex, Alton Pkwy Irvine, CA, USA), anti- Phospho-ULK1 (1:1000) (Ser555) (#5869, Cell Signaling technology, Danvers, MA, USA), anti-USP24 (1:1000) (13126-1-AP, Proteintech, Rosemont, IL, USA), anti-E2F4 (1:1000) (ab150360, Abcam, Cambridge, MA, USA), anti-E2F1 (1:1000) (#3742, Cell Signaling technology, Danvers, MA, USA), anti-γ-H2AX (1:1000) (AP0099, Abclonal, China), anti-CCNB1 (1:200) (sc-245, Santa Cruz Biotechnology, Santa Cruz, CA, USA), anti-TRAF6 (1:1000) (#8028, Cell Signaling technology, Danvers, MA, USA), anti-ATG3 (1:1000) (#3415, Cell Signaling technology, Danvers, MA, USA), anti-ATG4B (1:1000) (GTX115678, Genetex, Alton Pkwy Irvine, CA, USA), anti-ATG5 (1:1000) (#12994, Cell Signaling technology, Danvers, MA, USA), anti-ATG7 (1:1000) (#8558, Cell Signaling technology, Danvers, MA, USA), anti-ATG12 (#4180, Cell Signaling technology, Danvers, MA, USA), anti-ATG16L1 (1:1000) (#8089, Cell Signaling technology, Danvers, MA, USA), anti-mTOR (1:1000) (#ab2732, Abcam, Cambridge, MA, USA), anti-p-mTOR(S2448) (1:1000) (ab109268, Abcam, Cambridge, MA, USA), anti-Beclin-1 (1:1000) (#3495, Cell Signaling technology, Danvers, MA, USA), anti-ABCG2 (1:1000) (#42078, Cell Signaling technology, Danvers, MA, USA), anti-PD-L1 (1:1000) (GTX104763, Genetex, Alton Pkwy Irvine, CA, USA), and anti-actin (1:3000) (GTX26276, Genetex, Alton Pkwy Irvine, CA, USA) were used as the first antibodies. After incubation with primary antibodies for 12 h, PVDF membranes were then incubated with secondary immunoglobulin antibodies linked with horse radish peroxidase (Merck Millipore, Billerica, MA, USA) for 2 h. ECL western blotting detection system (Merck Millipore, Billerica, MA, USA) and ChemiDoc-it imager (UVP, Upland, CA, USA) were used for detecting signals.

### Whole-genome sequencing

A549 cell line was sequenced by a whole genome sequence. Genomic DNA materials were extracted using the QIAamp DNA Mini kit (QIAGEN, Germantown, MD, USA.) according to the manufacturer’s protocol. Following the manufacturer’s recommendations to add indices to each sample, using NEBNext® DNA Library Prep Kit. DNA sample preparations need 1.0 μg of DNA per sample. Randomly fragmented the genomic DNA to a size of 350 bp by shearing. DNA fragments were ligated with the NEBNext adapter and polished A-tailed for Illumina sequencing. Agilent 2100 Bioanalyzer analyzed for size distribution with purified PCR products (AMPure XP system) and quantified using real-time PCR. Variants are calling it by GATK, DELLY, and FREEC software.

### Fluorescence-activated cell sorting (FACS)

The experiment follows the methodology outlined by Wang et al. [3]. Cells were initially seeded in a 6-well plate, and the duration of culturing varied depending on the specific experiments conducted. Under appropriate cell density conditions, cells were washed with phosphate-buffered saline (PBS), and then were fixed with 75% ethanol at 4 °C for 2 h. Following fixation, cells were treated with PBS containing 0.1–0.15% Triton X-100 for 10–30 min. Next, cells were treated with 50 µg/ml propidium iodide, supplemented with RNase A, at 26 °C for 1 h. The stained cells were then analyzed using an Attune NxT Flow Cytometer (Thermo Fisher, USA).

### Transmission electron microscopy (TEM)

A549-T24 lung cancer drug resistant cells were treated with 5 µM of USP24-i-101 for 24 h, and then fixed with 4% formaldehyde for studying the cell morphology by using H-7650 TEM (TEM; Hitachi, Tokyo, Japan).

### Micro CT

Micro CT images of the lungs were obtained using a SkyScan 1276 (at Bruker Biospin, Billerica, MA). Mice were anesthetized with isoflurane. Scans were performed with the following parameters: source voltage and current (60 kV and 200 μA), imaging pixel size was 34.9999451 μm with 0.5 μm filter. The distance of the object to the source was set to 79.138 mm. The total scan time was approximately 20 min for the lung. Image processing was performed with DataViewer (Bruker-microCT NV, Kontich, Belgium) software and then rendered into 3D images in CTAn and CTVol softwares (Bruker-micro-CT NV, Kontich, Belgium).

### Masson’s trichrome staining

The lung tissues from mice were collected, routinely fixed in 4% formalin at 4 °C for 48 h and embedded in paraffin. Sections of 5-µm thickness were cut then deparaffinized. Using Masson’s trichrome kits (Abcam, ab150686) to measure the density of collagen fibers according to the manufacturer’ instruction.

### RNA-Seq

Total RNA was extracted and purified by a Quick-RNA MiniPrep Kit (Zymo Research, Irvine, CA, USA). For RNA Sequencing analysis, 3 μg of isolated total RNA was qualified and sequenced by Biotools Biotech Co. Ltd (Taipei City, Taiwan). The detailed methods are shown in the supplementary material.

### Immunoprecipitation assay

Cells were washed with PBS and resuspended with lysis buffer (25 nM, pH 7.5 Tris-HCl, 150 mM NaCl, 5 mM EDTA, 1% Triton X-100, 1% SDS). Cell extracts were prepared, and the protein concentration was determined using a bicinchoninic acid (BCA) protein assay kit. Immunoprecipitation was performed as previously described [27]. Briefly, an equal amount of protein was used in each experiment. Diluted 50 µl supernatant with 450 µl dilution buffer (50 mM, pH 8.0 Tris-HCl, 0.5% NP-40, 0.2 M NaCl, 0.5 mM EDTA) and incubated with antibodies against USP24 (1:200) (13126-1-AP, Proteintech, Rosemont, IL, USA) or anti-PD-L1 (1:200) (#13684, Cell Signaling technology, Danvers, MA, USA or Normal IgG (1:250, Santa Cruz)) at 4 °C for 16 h. The immunoprecipitated pellets were subsequently incubated with protein G-Sepharose at 4 °C for 3 h, washed three times with lysis buffer, and separated on SDS-PAGE.

### Cryo-EM single particle reconstruction

The recombinant USP24 used for cryo-EM analysis was purchased from OriGene with a concentration of 0.15 mg/ml (CAT#: TP329472, OriGene, USA), and an aliquot of 4 μl was loaded onto a glow-discharged grid coated with a holey carbon film (300 mesh, R2/1, Quatifoil GmbH, Germany). The vitrification of the grids was carried out using a Vitrobot system (Thermo Fisher Scientific, USA) with a waiting time of 3 s and blotting time of 4.5 s. The cryo-EM micrographs were acquired using a 200 kV Talos Arctica transmission electron microscope equipped with a Falcon III detector (Thermo Fisher Scientific, USA) with a magnification of ×120,000, corresponding to a pixel size of 0.86 Å/pixel. The micrographs were processed with motion correction and CTF estimation using cryoSPARC [28]. For the single particle reconstitution analysis, 16,213 particles were selected from a total of 57,814 particles picked from 2921 micrographs after three runs of 2D classification. The initial map was refined and polished through two rounds of homogenous refinement and was then visualized using Chimera.

### Statistical assay

All samples were used for statistical analysis. The investigator was aware of the sample allocation during the experiment and when assessing its outcome for all animal experiments. For all experiments, at least three independent biological replicates of each condition were analyzed. The estimated variation within each experiment group is similar. The difference between two groups was analyzed by a two-tailed unpaired Student’s t-test but used one-way ANOVA among three or more independent groups. The *P*-value, which is <0.05, was considered statistically significant. The center value is defined as the mean value, and s.e.m. is used to calculate and plot error bars from raw data.

## Results

### Targeting USP24 induces autophagy in vitro and in vivo

**Fig. 1:**
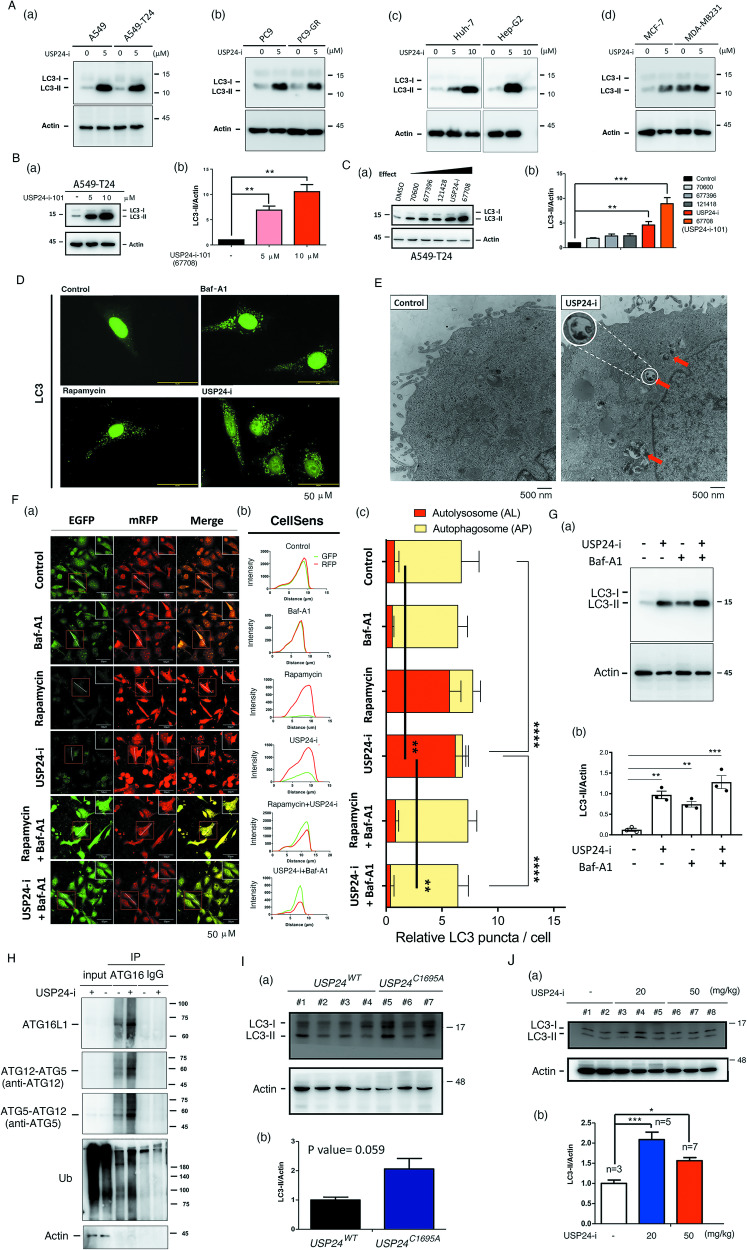
Targeting USP24 induces autophagy. **A** Various cancer cells, A549 and A549-T24 (a), PC9 and PC9-GR (b), Huh and Hep-G2 (c), MCF-7 and MDA-MB231 (d), were treated with 0, 5 or 10 μM USP24-i for 24 h, and then, the samples were harvested to study the level of LC3B with IB. **B** A549-T24 cells were treated with 0, 5 and 10 μM of the USP24-i analogues, USP24-i-101 for 24 h, and the samples were harvested to study the level of LC3-II with IB (a). The level of LC3-II was quantitated after three independent experiments (b). **C** USP24-i and various USP24-i-analogues (5 μM) were used to treat A549 cells for 24 h, and the level of LC3-II was studied by IB (a). The level of LC3-II was quantitated after three independent experiments (b). **D** A549 cells were treated with bafilomycin A1 (Baf-A1), rapamycin or USI24-i for 24 h, the level and localization of LC3-II were studied by IF. **E** The autolysosomes in the A549 cells with or without USP24-i treatment were studied by TEM. **F** A plasmid, pmRFP-EGFP-LC3, from Dr. Tamotsu Yoshimori was transfected into A549 cells for 24 h to study the signals of mRFP and EGFP by immunofluorescence microscopy (IF) (a). The signal of mRFP and EGFP was also analyzed by software in Carl Zeiss LSM780 (b), and the relative LC3 puncta per cell, mRFP (autolysosome, red) and mRFP+EGFP (autophagosome, yellow), in every cell were quantified (c). After three independent experiments were finished, the statistical analysis was performed by ANOVA (c). **G** A549-T24 cells treated with 5 μM USP24-i or 400 nM Baf-A1 for 8 h were also collected to study the level of LC3-II by western blotting (a), and the level of LC3-II was quantified, and statistical analysis was performed by t-test after three independent experiments (b). **H** A549-T24 cells with or without USP24-i treatment for 24 h, and then samples were collected for IP with anti-ATG16 antibodies and IgG. The IP samples were used to measure the levels of ATG16L1, ATG12-ATG5 and ubiquitination signal by IB. **I** The lung organs were collected from *USP24*^*WT*^ (*n* = 3) and *USP24*^*C1695A*^ (*n* = 3) mice to study the level of LC3-II by IB (a), and then, quantitation was performed (b). **J** *USP24*^*WT*^ mice were treated (i.p.) with 0 (*n* = 3), 20 mg/kg (*n* = 5) and 50 mg/kg (*n* = 7) for 24 h. The lung organs were harvested to study the level of LC3-II with IB (a), and the level of LC3-II was quantitated (b). All quantitation were analyzed by statistical analysis with a t test; *p < 0.05, **p < 0.01, ***p < 0.005, ****P < 0.001.

Our previous studies indicate that USP24 promotes drug resistance acquired through cancer therapy by stabilizing ABC transporters and inducing the genomic instability [3]. One study indicated that USP24 stabilized permeability glycoprotein (P-gp) and ATP binding cassette subfamily G member 2 (ABCG2) in a proteosome- and lysosome-dependent manner, respectively [3], implying that lysosomal function may be involved in USP24-mediated drug resistance. In this study, we found that 5 μM and 10 μM USP24-i (Fig. 1A) or USP24-i-101 (Fig. 1B) treatment induced LC3-II in several cell lines, including lung cancer cells A549 cells, Taxol-induced drug resistant A549 cells (A549-T24) (Fig. 1A(a)), PC9 cells and gefitinib-induced drug resistant PC9 cells (PC9-GR) (Fig. 1A(b)), liver cells, Huh-7 and HepG2 cells (Fig. 1A(c)), and breast cancer cells, MCF7 and MDA-MB231 cells (Fig. 1A(d)). In the past, we have measured the effects of USP24-i and its analogs, 70600, 677396, 121428 and 67708 (USP24-i-101) on Taxol-induced cytotoxicity, and found that only 67708 (USP24-i-101) can increase Taxol-induced cytotoxicity than USP24-i. Herein we found a positive correlation between targeting USP24-mediated LC3-II signaling and the cytotoxicity of Taxol in drug resistance, implying that targeting USP24-induced autophagy might be important for inhibiting drug resistance during cancer therapy (Fig. 1C). According to the previous studies, both autophagic inhibition and activation can cause LC3B accumulation. To study the exact effect of targeting USP24 on autophagy, we used USP24-i, the autophagy inhibitor bafilomycin-A1 (Baf-A1), and the activator rapamycin to study the LC3-II level (Fig. 1D). Indeed, the LC3-II levels in the cytoplasm of A549 cells treated with USP24-i, bafilomycin-A1 and rapamycin were increased compared to the controls (Fig. 1D). Next, we studied the effect of targeting USP24 on autophagy by TEM (Fig. 1E). The autolysosomes were found in the USP24-i-treated cells (red arrow) (Fig. 1E). We also used a pH-sensitive plasmid, pcDNA3.1-mRFP-EGFP-LC3 which sometimes referred to as traffic light assay, is one test to assess autophagic flux in mammalian cells [29, 30]. In this assay, autophagosomes appear as yellow puncta. Unlike RFP, GFP fluorescence being pH sensitive, gets quenched in the acidic pH environment of autolysosomes and thus appears red (Fig. 1F(a)). The data indicated that autolysosome-induced acidic pH induced higher mRFP-LC3 but lower EGFP expression in rapamycin- and USP24-i-treated cells (Fig. 1F(a)), resulting in the overlap (yellow color) between EGFP and mRFP in the control, Baf-A1-, rapamycin/Baf-A1- and USP24-i/Baf-A1-treated cells being higher than that in the rapamycin- or USP24-i-treated A549 cells (red color), indicating that USP24-i treatment induces autolysosome (Fig. 1F(b) and Fig. 1F(c)). The level of LC3-II was significantly accumulated in the presence of USP24-i and Baf-A1 compared to Baf-A1 treatment only (Fig. 1G(a) and Fig. 1G(b)). In addition, ATG5, ATG12 and ATG16L1 will form a complex in autophagy activation [31], herein we found that USP24-i increased the interaction among ATG5, ATG12 and ATG16L1 in A549-T24 cells (Fig. 1H). Finally, the level of LC3-II was also studied in vivo (Fig. 1I, Fig. 1J and Supplementary Fig. 1). *USP24*^*C1695A*^ mice constructed by CRISPR/Cas9 were used to study the role of USP24 in autophagy (Supplementary Fig. 1). LC3-II was increased in USP24 knockout mice (Fig. 1I) and USP24-i-treated mice (Fig. 1J). Overall, targeting USP24 by USP24-i induces autophagy in vitro and in vivo.

### Targeting USP24 decreases E2F4 and TRAF6 levels to induce autophagy in interphase and mitotic cancer cells

**Fig. 2:**
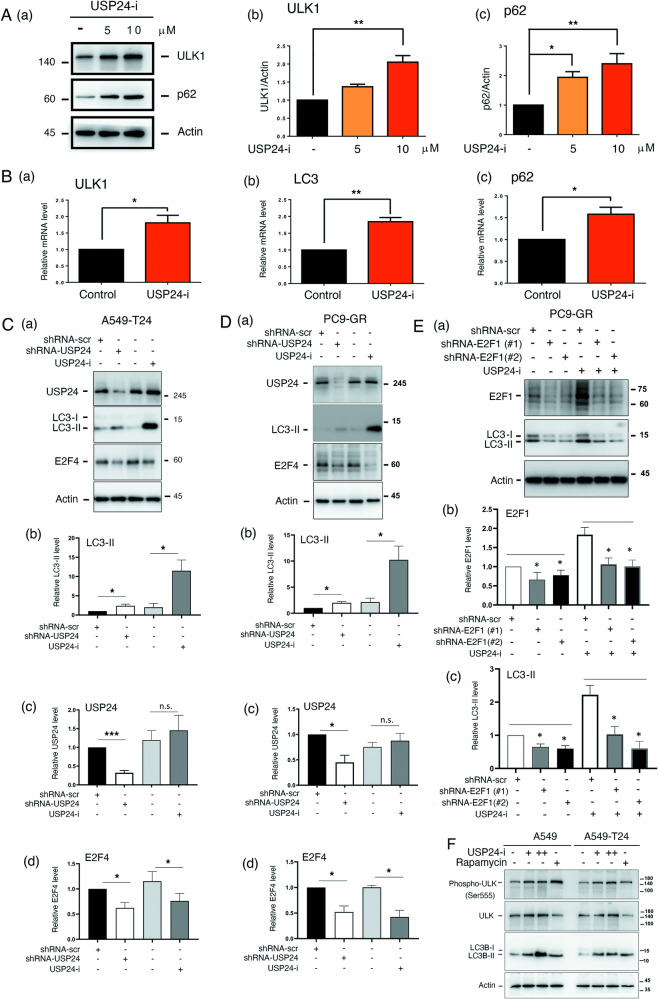
Targeting USP24 induces the expression of ULK1, LC3 and p62 in lung cancer cells. A549 cells were treated with USP24-i (5 μM and 10 μM) for 24 h, the protein (**A**) and mRNA (**B**) levels of ULK1, LC3 and p62 were studied by IB (**A** (a)) and qPCR (**B**), and the levels of protein, ULK1 (A(b)) and p62 (A(c)), and mRNA, ULK1 (B(a)), LC3 (B(b)) and p62 (B(c)), were quantitated. USP24 was silenced with sh-USP24 or inhibited with 5 μM USP24-i in A549-T24 (**C** (a)) and PC9-GR (**D** (a)) cells, and then, the levels of LC3-II (**C**(b), (**D**(b)), USP24 (**C** (c), (**D** (c)) and E2F4 (**C**(d), (**D**(d)) were studied by IB. The levels were quantitated after three independent experiments. **E** (a) E2F1 was silenced by sh-E2F1 (#1 and #2) in PC9-GR cells with or without USP24-i treatment. The levels of E2F1 (**E** (b)) and LC3-II (**E** (c)) were studied by IB. After three independent experiments, the data were quantitated. **F** A549 and A549-T24 cells were treated with USP24-i and Rapamycin. The samples were collected to measure the levels of phosphor-ULK, ULK, LC3-II and actin by IB. All quantitation data were analyzed by statistical analysis with a t test; *p < 0.05, **p < 0.01, ***p < 0.005.

**Fig. 3:**
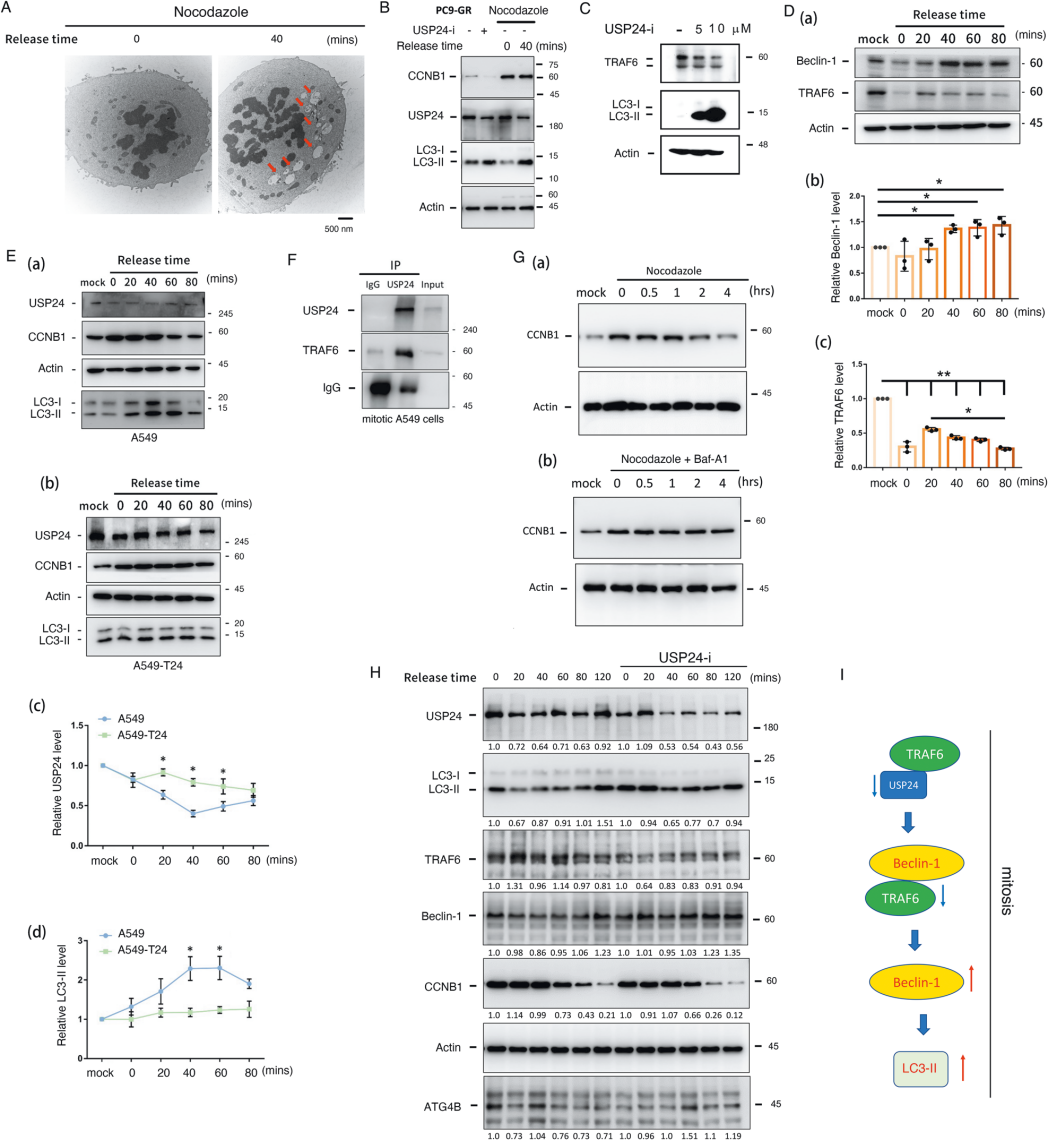
USP24 downregulation in mitosis induces autophagy. **A** A549 cells were synchronized at G2/M with nocodazole treatment, and then, nocodazole was removed to collect samples at 0 and 40 min. The autophagosomes and autolysosomes were studied by TEM. **B**, **C** PC9-GR cells were treated with 5 and 10 μM USP24-i, synchronized with nocodazole for 18 h and released for 2 h. Samples were collected to study the levels of the indicated proteins with antibodies against these proteins. **D** The levels of beclin-1, TRAF6 and actin in mitotic A549 cells were determined by IB (**D** (a)), and the levels were quantitated after three independent experiments (**D** (b) and (c)). **E** A549 and A549-T24 cells were synchronized at G2/M with nocodazole treatment, and then, nocodazole was removed to collect samples at 0, 20, 40, 60 and 80 min. The levels of USP24, CCNB1, LC3-II and actin were studied by IB (**E** (a) and (b)), and the levels were quantitated after three independent experiments (**E** (c) and (d)). **F** A549 cells were synchronized at G2/M with nocodazole treatment, and then, nocodazole was removed to collect samples at 40 min. The interaction among USP24 and the indicated proteins was studied by IP with anti-USP24 antibodies and IB with antibodies against the indicated proteins. **G**, **H** A549 cells were synchronized with nocodazole treatment for 18 h, and then released from nocodazole (G(a)), and further treated with 10 nM bafilomycin A1 (Baf-A1) (**G** (b)) or 5 μM USP24-i (**H**) for 2 h. Samples were harvested to study the protein levels with IB, and then the levels of indicated proteins were quantitated. Summary of the mechanism of how USP24-induced autophagy during mitosis (**I**). All the quantitated data were analyzed by statistical analysis with a t test; *p < 0.05, **p < 0.01, ***p < 0.005.

We also investigated the molecular mechanism of how USP24-mediated autophagy in interphase and mitosis (Fig. 2, Fig. 3 and Supplementary Fig. 2). At first, the effect of USP24-i treatment on global gene expression profile was studied by RNA-seq. Most of the autophagy-related genes were upregulated in the USP24-i- and Taxol-treated A549-T24 cells compared to those with Taxol treatment only (Supplementary Fig. 2A– D). However, when we studied the effect of targeting USP24 on the levels of ATGs, there was no significant difference in the levels of ATGs (Supplementary Fig. 2E). In addition, knockdown of USP24 did not inhibit the phosphorylation of mTOR, which is an important pathway of autophagy induced by rapamycin Supplementary Fig. 2F). USP24-i treatment in A549 cells increased the levels of ULK1 and p62 (Fig. 2A) and the mRNA levels of ULK1, LC3 and p62 (Fig. 2B), implying that USP24 may stabilize one transcription factor, thereby recruiting to the promoters of ULK1, LC3 and p62. Previous studies have indicated that E2F1 can positively regulate the transcriptional activities of ULK1 and LC3. Our previous studies indicated that USP24 can stabilize E2F4 and subsequently inhibit E2F1 [32]. Herein, we found that knockdown of USP24 by shUSP24 or targeting USP24 by the USP24 inhibitor USP24-i in A549-T24 and PC9-GR drug-resistant lung cancer cell lines increased the LC3 levels but decreased the E2F4 levels (Fig. 2C, D). Because E2F4 can negatively regulate E2F1 expression, the levels of E2F1 and LC3-II were studied under knockdown of E2F1 and USP24-i treatment in PC9-GR calls (Fig. 2E). The data indicated that USP24-i treatment increased E2F1 and LC3-II expression. Knockdown of E2F1 inhibited the expression of LC3-II under USP24-i treatment (Fig. 2E). In addition, previous studies also indicate that ULK is phosphorylated at Ser555 under autophagy condition [33]. Herein we also found that USP24-i treatment increased the phosphorylation of ULK at Ser555 (Fig. 2F). In summary, targeting USP24 by USP24-i inhibits E2F4, thereby increasing E2F1 expression, and subsequently increasing ULK1 and LC3 expression, resulting in autophagy in interphase.

In previous studies, we showed that USP24 is downregulated during mitosis to destabilize securin, which will be beneficial for mitotic progression [32]. Therefore, a decrease in USP24 during mitosis may also induce autophagy during mitosis (Fig. 3). Interestingly, autolysosomes were found in mitotic cells (red arrow), indicating that USP24-mediated autophagy occurs not only in interphase but also in mitosis (Fig. 3A). Mitotic A549 cells treated with USP24-i increased LC3-II levels and decreased levels of TRAF6, which is one of the E3 ligases of beclin-1 (Fig. 3B and Fig. 3C). Next, we found that the level of beclin-1 was increased and TRAF6 was decreased in mitotic A549 cells (Fig. 3D). In addition, we found that USP24 levels were significantly decreased in mitotic A549 cells (Fig. 3E(a) and Fig. 3E(c)) but only slightly decreased in A549-T24 cells (Fig. 3E(b) and Fig. 3E(c)). LC3-II was significantly increased in mitotic A549 cells but not in A549-T24 cells (Fig. 3E(a), Fig. 3E(b) and Fig. 3E(d)), implying that the downregulation of USP24 may induce autophagy to maintain genomic stability in drug-sensitive lung cancer cells but not in drug-resistant lung cancer cells (Fig. 3E). USP24, as a deubiquitinase decreased during mitosis, might be related to a decrease in TRAF6. The interaction between USP24 and TRAF6 in mitosis was studied (Fig. 3F). The data indicated that USP24 can interact with TRAF6 in mitotic A549 cells (Fig. 3F). In addition, inhibition of autophagy by bafilomycin A1 delayed the decrease in CCNB1 during mitosis, indicating that autophagy is beneficial for mitotic progression (Fig. 3G). Furthermore, USP24-i was also used to treat mitotic A549 cells to study the levels of LC3-II, Beclin-1, CCNB1, ATG4B and TRAF6 (Fig. 3H). The data revealed that the levels of LC3-II and Beclin-1 were increased, but those of TRAF6 and CCNB1 were decreased in the present of USP24-i, suggesting that USP24-i targeting USP24 may be beneficial for mitotic progression (Fig. 3H). In summary, downregulation of USP24 during the mitotic period may increase the degradation of TRAF6, thereby increasing the protein stability of beclin-1 and leading to autophagic induction (Fig. 3I).

### Targeting USP24 induces autophagy to inhibit drug resistance during lung cancer therapy

**Fig. 4:**
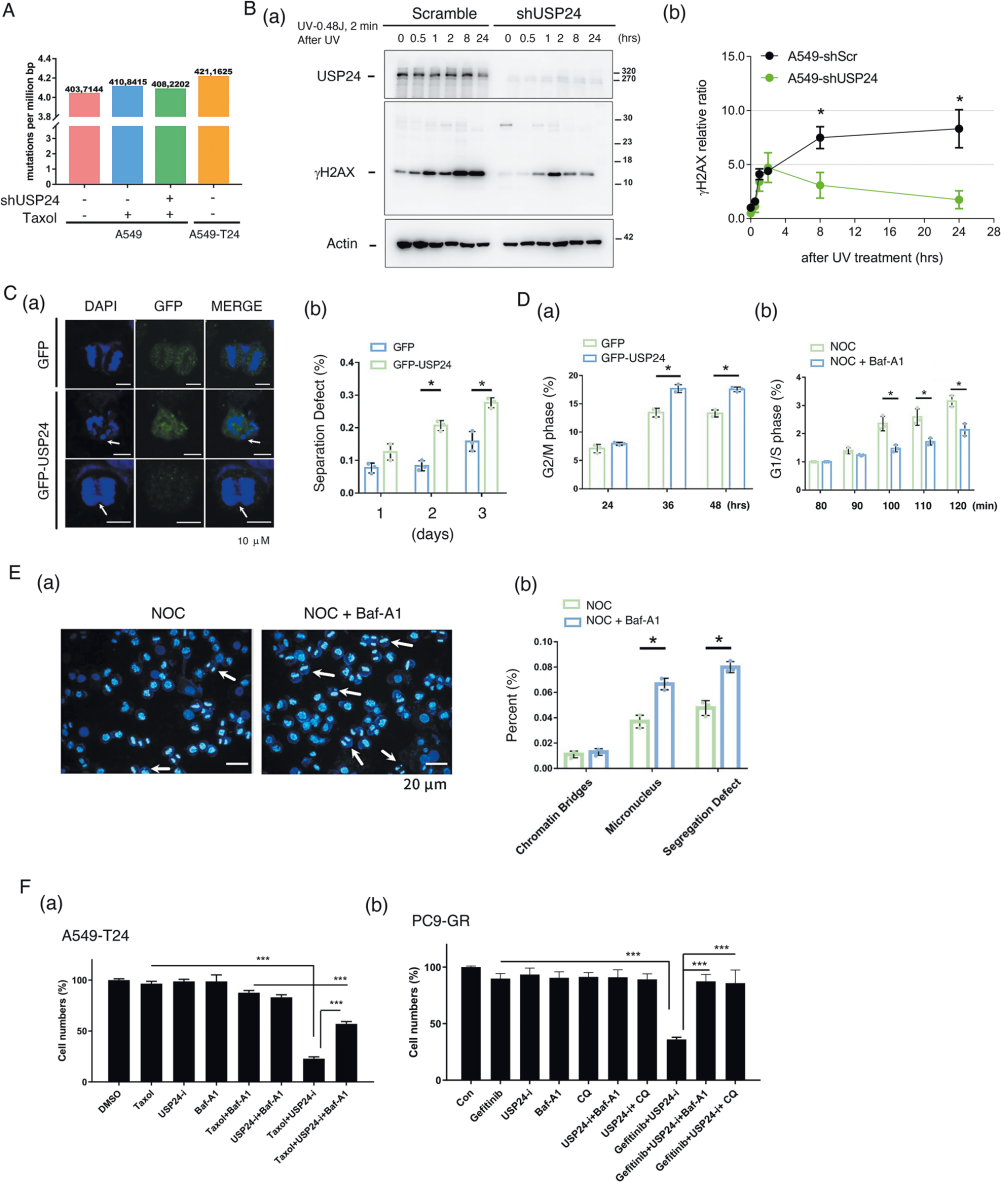
Targeting USP24 induces autophagy to inhibit drug resistance acquired lung cancer drug resistance. **A** USP24 was knocked down by lenti-shUSP24 virus in A549 and Taxol-induced drug-resistant A549 cells (A549-T24). The genomic mutation profiles were studied by whole genome sequencing (WGS). **B** USP24 was silenced by lenti-shUSP24 virus in A549 cells with ion irradiation. Cell extracts were harvested at 0 h, 0.5 h, 1 h, 2 h, 8 h and 24 h after irradiation to study the levels of the indicated proteins with IB (a), and the level of γ-H2AX was quantified after three independent experiments (b). **C** GFP-USP24 was expressed in A549 cells for 1 day, 2 days and 3 days, and the chromosome separation defect in anaphase was studied by IF (a), and the separation defect was quantified after three independent experiments (b). **D** GFP and GFP-USP24 were expressed in A549 cells, and the cells arrested in G2/M phase were studied by flow cytometry (a). A549 cells were arrested at G2/M by nocodazole treatment, and mitotic progression was then studied with or without 10 nM of the autophagy inhibitor bafilomycin-A1 (Baf-A1) by flow cytometry (b). **E** A549 cells were synchronized in G2/M phase by 100 ng/ml nocodazole with or without bafilomycin-A1 (Baf-A1) treatment. The chromosome segregation defect, chromatin bridge, micronucleus and segregation defect, were studied by IF (a), and the chromosome separation defect was quantified after three independent experiments (b). **F** Taxol-induced drug-resistant A549 cells (A549-T24) (a) and gefitinib-induced drug resistant PC9 cells (PC9-GR) (b) were treated with 24 nM Taxol or 5 μM gefitinib with or without 5 μM USP24-i and 10 nM Baf-A1 or 5 μM chloroquine (CQ). Cytotoxicity was studied by cell counting. After three independent experiments, the data were quantitated, and statistical analysis was performed with a t test; *p < 0.05, **p < 0.01, ***p < 0.005.

What is the role of targeting USP24-mediated autophagy in drug resistance? Because tumor heterogeneity is related to drug resistance, herein we studied the role of USP24 in genomic integrity by whole genome sequencing (WGS) (Fig. 4). All the DNA sequences were compared to the Human sequence of PubMed (GRCH 38). The data indicated that Taxol treatment, including short-term treatment and long-term Taxol-induced A549 drug resistance (A549-T24), increased the mutation rate, but reduced the mutation efficacy under knockdown of USP24 condition, indicating that USP24 might be involved in genomic instability (Fig. 4A). The effect of USP24 on repair activity was also studied by irradiation here (Fig. 4B). The same irradiation exposure in A549 cells with or without USP24 knockdown caused the same DNA damage, and then measured the DNA damage repair activity in the early period (Fig. 4B). The data indicated that knockdown of USP24 significantly decreased the levels of γ-H2AX in cells with irradiation, suggesting that loss of USP24 results in a higher DNA damage repair activity in response to the same DNA damage repair by irradiation (Fig. 4B). Our previous studies revealed that USP24 was decreased in mitosis and involved in chromosome separation [32]. Herein, we also found that GFP-USP24 overexpression in mitotic cells caused chromosome separation defects and increased the G2/M arrest in cells, implying that a decrease in USP24 during mitosis is beneficial to the genomic integrity during mitosis (Fig. 4C). In Supplementary Fig. 4, overexpression of GFP-USP24 led to cell arrest at mitosis for more than 12 h and caused unequal segregation finally (Supplementary Fig. 4). GFP-USP24 overexpression increased the cells stayed in G2/M phase, indicating that downexpression of USP24 is advantage for mitosis progression (Fig. 4D(a)). Next, we studied the effect of autophagy on mitosis. A549 cells were synchronized in G2/M, and then treated with the autophagy inhibitor, Baf1, to study the cell cycle (Fig. 4D(b)) and chromosome separation defects (Fig. 4E). The data indicated that inhibition of autophagy decreased the cell entry from G2/M (Fig. 4D(b)), and increased chromosome separation defects, micronuclei and segregation defects (Fig. 4E), indicating that autophagic induction during mitosis could maintain genomic stability.

Next, the effect of targeting USP24-induced autophagy on chemotherapy- and targeted therapy-induced drug-resistant lung cancer cells was studied (Fig. 4F and Supplementary Fig. 3). The data indicated that Taxol, USP24-i or Baf-A1 treatment alone cannot kill Taxol-induced drug resistant A549 cells (A549-T24). However, Taxol and USP24-i cocktail treatment significantly killed cells (73.6%). Taxol, USP24-i and Baf-A1 treatment decreased cytotoxicity (34.2%), suggesting that USP24-i-induced autophagy contributes to inhibiting drug resistance acquired by chemotherapy (Fig. 4F(a)). Next, we also used gefitinib-induced PC9 drug-resistant cells (PC9-GR) to study the effect of targeting USP24 induced autophagy on inhibiting drug resistance acquired by targeted therapy (Fig. 4F(b), Supplementary Fig. 3A, B). The data indicated that gefitinib, USP24-i, Baf-A1, chloroquine (CQ), USP24-i + Baf-A1, and USP24-i + CQ treatment did not lead to cell death. Gefitinib and USP24-i cocktail treatment significantly induced cell death but abolished this effect by inhibiting autophagy with Baf-A1 or CQ treatment, suggesting that targeting USP24-induced autophagy is essential for the effect of USP24-i on blocking drug resistance acquired by targeted therapy. In addition, we studied the effect of rapamycin-induced autophagy on blocking drug resistance (Supplementary Fig. 3C, D). The data indicated that, similar to USP24-i treatment, rapamycin-induced autophagy also increased the cytotoxicity of Taxol- or gefitinib-treated drug-resistant A549-T24 and PC9-GR lung cancer cells (Supplementary Fig. 3C, D). In addition, rapamycin-induced autophagy was also essential for the effect of USP24 in inhibiting the drug resistance to Taxol and gefitinib (Supplementary Fig. 3C, D). In summary, targeting USP24-induced autophagy is important for blocking drug resistance acquired by chemotherapy and targeted therapy.

### USP24 functional knockout inhibits drug resistance in drug resistant mice with gefitinib-induced *EGFR*^*L858R*^ lung cancer

**Fig. 5:**
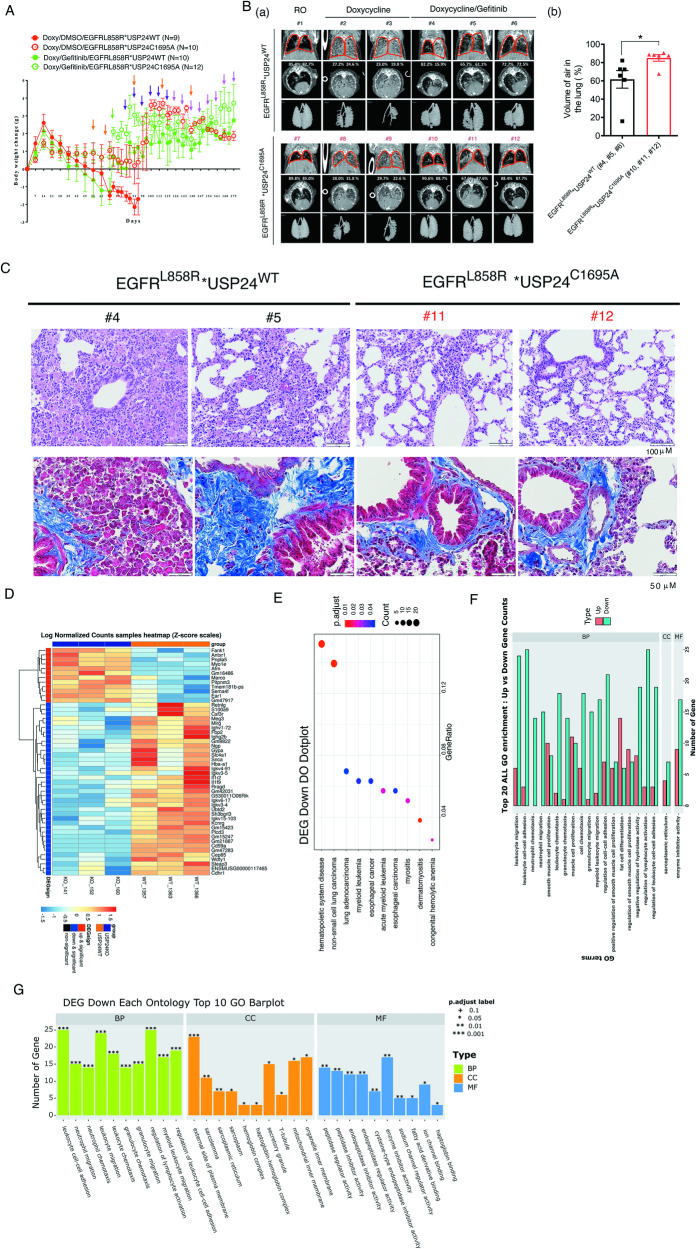
USP24 functional knockout inhibits drug resistance in mice with EGFR^L858R^ lung cancer induced by gefitinib treatment by regulating gene expression involved in lung cancer progression and drug resistance during drug resistance in vivo. **A** *EGFR*^L858R^*USP24^*WT*^ and *EGFR*^L858R^*USP24^*C1695A*^ mice were treated with 10 mg/l doxycycline in the drinking water for 6 weeks, and then treated with gefitinib for a long time. Tumor growth in vivo was studied by body weight every week (**A**) and micro-CT (**B** (a)). The micro-CT signal was quantitated by CTAn software from Bruker (**B** (b)). After sacrifice, the pathology in the lung organs was studied by H&E staining (C, upper panel), and the fibrosis (deep red) and collagen (blue) were studied by Masson’s trichrome staining (**C**, lower panel). Total RNA specimens were isolated from the lungs of gefitinib-induced *EGFR*^L858R^*USP24^*WT*^ and *EGFR*^L858R^*USP24^*C1695A*^ drug resistant mice to study the gene expression profile by RNA-Seq (*EGFR*^L858R^*USP24^*C1695A*^/ *EGFR*^L858R^*USP24^*WT*^). (**D**). Heatmap (**E**). DEG Down DO Dotplot (**F**). Top 20 ALL GO enrichment (**G**). DEG Down Each Ontology Top 10 GO Barplot. The statistical analysis was performed by one way ANOVA.

Recently, we successfully constructed a drug resistant animal model (Fig. 5). Doxycycline in the drinking water was drank by the mice to form a complex with scgb101-triggered rTA in the lung, thereby was recruited to the promoter of *EGFR*^*L858R*^ to express EGFR^L858R^, resulting in lung cancer formation [34]. We used the body weight of mice and the signal of microcomputed tomography (micro-CT) to monitor the tumor size under gefitinib longtime treatment. We also constructed USP24 functional knockout mice by CRISPR/Cas9, *USP24*^*C1695A*^ (Supplementary Fig. 1). Here, we used *EGFR*^L858R^*USP24^*WT*^ and *EGFR*^L858R^*USP24^*C1695A*^ mice to study the role of USP24 in the drug resistance of lung cancer acquired by gefitinib treatment (Fig. 5). The body weight of the mice was monitored every week until that the body weight did not increase after gefitinib injection (15 mg/kg, i.p.), implying that gefitinib is not effective (Fig. 5A). Before sacrifice, the tumors in mice were detected by micro-CT (Fig. 5B). The data indicated that there was no signal found in the RO-treated wild-type and USP24 knockout mice (#1 and #7 mice), indicating that no tumors were in the lung. Doxycycline treatment dramatically increased the signal in the lungs of the wild-type and USP24 knockout mice, suggesting that doxycycline successfully induced tumors in the lung of the *EGFR*^L858R^*USP24^*WT*^ (#2 and #3 mice) and *EGFR*^L858R^*USP24^*C1695A*^ (#8 and #9 mice) mice. Furthermore, after 172 days of gefitinib treatment, a partial signal was found in the *EGFR*^L858R^*USP2^*WT*^ mice (#4, #5 and #6 mice) but not in the *EGFR*^L858R^*USP24^*C1695A*^ mice (#10, #11 and #12 mice), suggesting that loss of USP24 inhibits targeted therapy-induced acquired drug resistance (Fig. 5B(a)). We also used Image J software to quantitate the air volume in the lungs of the various mice (Fig. 5B(b)). The data indicated that the air volume in the lungs of doxycycline-induced *EGFR*^L858R^*USP24^*WT*^ mice and *EGFR*^L858R^*USP24^*C1695A*^ mice was 60% and 81% respectively after 175 days gefitinib treatment, indicating that loss of USP24 inhibits gefitinib-induced drug resistance (Fig. 5B(b)). After sacrifice of mice, the lung pathology was studied (Fig. 5C). The data indicated that the tumor area in the gefitinib-induced *EGFR*^L858R^*USP24^*C1695A*^ drug-resistant mice was inhibited compared with that in the gefitinib-induced *EGFR*^L858R^*USP24^*WT*^ drug-resistant mice, indicating that USP24 promotes drug resistance acquired during cancer therapy (Fig. 5C, upper panel). In addition, fiber and collagen analysis by Masson trichrome staining indicated that collagen (blue) was decreased in the gefitinib-induced *EGFR*^L858R^*USP24^*C1695A*^ drug resistant mice compared to gefitinib-induced *EGFR*^L858R^*USP24^*WT*^ drug resistant mice, implying that USP24 might be involved in extracellular matrix (ECM) modelling during cancer therapy (Fig. 5C, lower panel).

Systemic gene expression profiling in the *EGFR*^L858R^*USP24^*WT*^ and *EGFR*^L858R^*USP24^*C1695A*^ mice with gefitinib treatment until drug resistance was performed with RNA-Seq. (Fig. 5D–G). The data indicated that 419 genes were downregulated, and 274 genes were upregulated in the lung tumors of the *EGFR*^L858R^*USP24^*C1695A*^ mice compared to the *EGFR*^L858R^*USP24^*WT*^ mice (Supplementary Fig. 5). After analysis of these genes, as shown by the DEG DO Dotplot, loss of USP24 enzyme activity was significantly involved in several diseases, including hematopoietic system disease, non-small cell lung carcinoma, lung adenocarcinoma, myeloid leukemia, myositis and so on (Fig. 5D and Fig. 5E), suggesting that USP24 is really involved in drug resistance during cancer therapy. By using GO enrichment to study the involved pathways, we found that many genes related to leukocyte migration, cell–cell adhesion, cell proliferation, cell differentiation and so on were regulated by USP24 under drug resistant conditions (Fig. 5F). In addition, the DEG Each Ontology GO Barplot addressed the related pathways (Fig. 5G). The data indicated that migration, mitochondrial inner membrane, ion transportation and fatty acid synthesis and son on were regulated by USP24 (Fig. 5G). Thus, loss of USP24 enzyme activity inhibits gefitinib-induced drug resistance by regulating the expression of genes involved in cancer, ECM and other cells around cancer cells.

### PD-L1 and ABCG2 upregulation in drug-resistant lung cancer cells was regulated by USP24

**Fig. 6:**
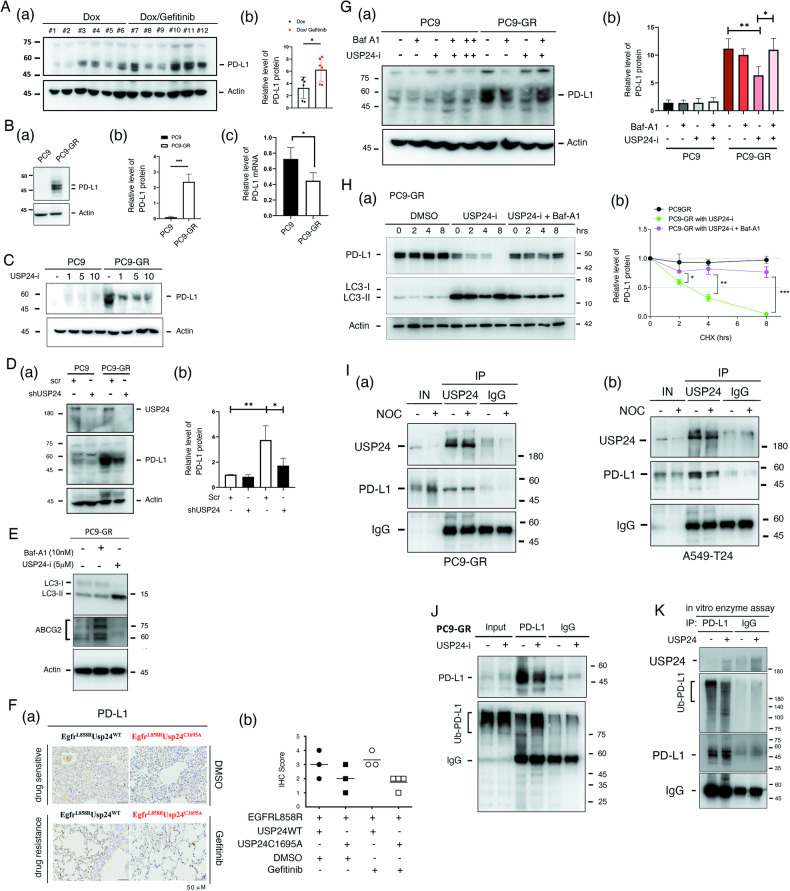
Several drug resistance-related proteins are upregulated in drug resistant mice. **A**, **B** The level of PD-L1 in doxycycline-induced *EGFR*^*L858R*^ lung cancer mice with or without gefitinib-induced drug resistance (**A** (a) and (b)) and in PC9 and PC9-GR (gefitinib resistant cells) (**B**, (a) and (b)) were studied by IB with anti-PD-L1 antibody, and the level of PD-L1 was quantified and statistical analysis by one way ANOVA (**A** (b)) or by t-test assay (**B** (b)). The mRNA levels of PD-L1 in PC9 and PC9-GR cells were studied by qPCR (**B**(c)). **C**–**E**. PC9 and PC9-GR cancer cells were treated with various doses of USP24-i for 24 h with or without 10 nM bafilomycin A1 (Baf-A1) treatment (**C**, **E**) or knockdown of USP24 with lenti-shRNA-USP24 viruses for 3 days (**D**), and the levels of ABCG2, USP24, LC3-II and PD-L1 were studied by IB (D(a)), and the level of PD-L1 was quantified after three independent experiments (**D**(b)). **F** The level of PD-L1 in mice with doxycycline-induced *EGFR*^L858R^*USP24^*WT*^ and doxycycline-induced *EGFR*^L858R^*USP24^*C1695A*^ lung cancer mice with or without gefitinib-induced drug resistance was measured by IHC with anti-PD-L1 antibodies (a) and were quantified with scoring the signal of PD-L1 (no expression = 0, low expression = 1, intermediate expression = 2, high expression = 3) (b). **G** USP24 stabilizes PD-L1 in cancer drug resistance. PC9 and PC9-GR cancer cells were treated with various doses of USP24-i for 24 h with or without 10 nM bafilomycin A1 (Baf-A1) treatment. The level of PD-L1 was studied by IB with anti-PD-L1 antibodies (a), and the level of PD-L1 was quantified after three independent experiments (b). All the experiments were performed at least three times, and then, the quantitation and statistical analysis were performed by a t test; *p < 0.05, **p < 0.01. **H** PC9-GR cells treated with cycloheximide and Baf-A1 and collected the samples at indicated times for studying the level of PD-L1 by IB with anti-PD-L1 antibodies (a), and the level of PD-L1 was quantified after three independent experiments (b). **I** PC9-GR cells (a) and A549-T24 cells (b) with or without nocodazole treatment were harvested to study the interaction of USP24 and PD-L1 by IP with anti-USP24 antibodies and IB with anti-PD-L1 antibodies. **J** Cell extracts harvested from PC9-GR cells with or without 5 μM USP24-i treatment in the presence of 10 μM MG132 were used to study the ubiquitination of PD-L1 by IP with anti-PD-L1 and IB with anti-Ubiquitin antibodies. **K** The in vitro enzyme assay was studied by using purify USP24 recombinant protein and PD-L1 from PC9-GR cells by IP with anti-PD-L1 antibodies. All the experiments were performed at least three times, and then, the quantitation and statistical analysis were performed by a t test; *p < 0.05, **p < 0.01, ***p < 0.005.

Our drug-resistant lung cancer animal model (Fig. 5) not only had cancer cells but also had an intact tumor-associated microenvironment (TAM). Because immune therapy is important for cancer therapy, our animal models are highly suitable for studying the interaction between cancer cells and TAMs. First, we found that the level of PD-L1 was increased in the gefitinib-induced drug-resistant mice (Fig. 6A). In addition, the protein level of PD-L1 was also significantly increased in PC9-GR cells compared to PC9 cells (Fig. 6B(a) and Fig. 6B(b)) but was decreased in the mRNA level (Fig. 6B(c)). Targeting USP24 by USP24-i in PC9-GR cells decreased the level of PD-L1 (Fig. 6C). Knockdown of USP24 in PC9 and PC9-GR cells significantly decreased the level of PD-L1 (Fig. 6D). Because our previous studies also found that USP24 stabilizes ABCG2 in drug-resistant cells [3], herein, we found that ABCG2 protein levels were significantly increased after inhibition of autophagy by bafilomycin-A1 (Baf-A1), but was decreased under USP24-i treatment, suggesting that USP24-i-induced autophagy is critical for decrease in PD-L1 (Fig. 6E). In addition, the level of PD-L1 was increased in PC9-GR compared to PC9 but can be inhibited under USP24-i treatment. We found that the PD-L1 level was significantly decreased in the doxycycline-induced *EGFR*^L858R^*USP24C^*1695A*^ drug-sensitive and drug-resistant mice compared to that in the doxycycline-induced *EGFR*^L858R^*USP24^*WT*^ drug-sensitive and drug-resistant mice respectively, suggesting that USP24 can stabilize PD-L1 in lung cancer mice, which is involved in drug resistance during cancer therapy (Fig. 6F). However, Baf-A1 treatment can reverse this effect of USP24-i, indicating that targeting USP24-induced autophagy is involved in the degradation of PD-L1 and ABCG2 (Fig. 6G). Studying the protein stability of PD-L1 found that the protein stability of PD-L1 was decreased in USP24-i-treated PC9-GR cells, but can be rescued by Baf-A1 treatment, indicating USP24-i-induced autophagy increases PD-L1 degradation (Fig. 6H). Interestingly, we found that USP24 can interact with PD-L1 in interphase and mitotic cells, implying that USP24 may stabilize PD-L1 in cancer drug-resistant cells PC9-GR (Fig. 6I(a)) and A549-T24 cells (Fig. 6I(b)). USP24-i treatment in PC9-GR cells increased the ubiquitination signal of PD-L1 (Fig. 6J). The in vitro enzyme assay of purify recombinant USP24 protein was used to study the ubiquitination of PD-L1 (Fig. 6K). The data indicated that USP24 decreased the ubiquitination signal of PD-L1, suggesting that PD-L1 is the substrate of USP24 (Fig. 6K). In summary, USP24-i targeting USP24 activates autophagy to promote the degradation of ABCG2 and PD-L1, leading to the inhibition of drug resistance acquired from lung cancer therapy.

### Targeting USP24 by USP24-i-101 inhibits drug resistance in vivo

**Fig. 7:**
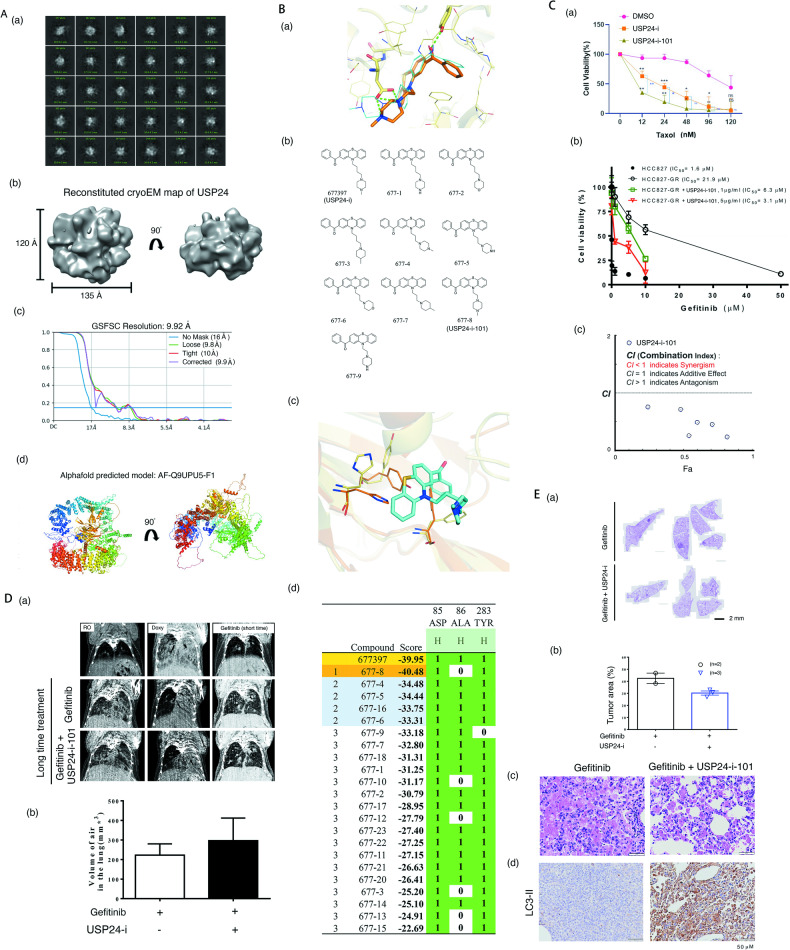
Optimization of USP24-i and targeting USP24 in *EGFR*^*L858R*^ lung cancer drug resistant mice. **A** The preliminary cryo-EM structure of USP24 showed a molecular morphology consistent with the model predicted by Alphafold (a). Representative particle images after 2D classification clustering (b). The reconstituted cryo-EM map shown in top view and side view (c). GSFSC plot showing that the map resolution is 9.9 Å (d). **B** The Alphafold predicted model displayed in similar orientation as the cryo-EM map. The interaction between the catalytic motif and USP24-I (a) and (c). The USP24-i and its analogues (b), and the score of the docking affinity score between the USP24 catalytic domain and USP24 inhibitors (d). **C** A549-T24 cells were treated with Taxol and USP24 inhibitors, USP24-i and USP24-i-101, to study cell viability by cell counting (a). HCC827 and its gefitinib drug-resistant cells, HCC827-GR, were treated with gefitinib and USP24-i to study the cytotoxicity of gefitinib by cell counting (b), and the combination index (CI) and dose reduction index (DRI) were analyzed by CompuSyn software (c). **D** *EGFR*^L858R^*USP24^*WT*^ mice were treated with 10 mg/l doxycycline in the drinking water for 6 weeks, and then treated with gefitinib (20 mg/kg) and USP24-i-101 (10 mg/kg) for 217 days. Tumor growth in vivo was studied by micro-CT (a), and the volume of air in the lung was calculated by CTAn software from Bruker (b). **E** After sacrifice, the pathology in the lung organs was studied by H&E staining, and the tumor area in the lung tissues was quantitated (a) and (c), and the tumor area was studied by ImageJ (b). The level of LC3-II in the lung organs was studied by IHC with anti-LC3-II antibodies (d). All the experiments were performed at least three times, and then, the quantitation and statistical analysis were performed by a t test; *p < 0.05, **p < 0.01, ***p < 0.005.

Our previous studies identified a specific USP24 inhibitor, NCI677397, which can inhibit the drug resistance of cancer acquired by Taxol treatment [3]. To evaluate the binding site of this inhibitor, we attempted to investigate the protein structure of USP24 complexed with USP24-i by using cryo-EM single particle analysis. After acquisition of 2,921 micrographs for the purified recombinant USP24, the particle images were selected and clustered for 2D classification and 3D structure reconstitution (Fig. 7A(a) and Fig. 7A(b)). However, the current resolution of the constructed map is only 9.9 Å, which is not sufficient to reveal the ligand binding site in detail. In contrast, the Alphafold-predicted structure of USP24 revealed a ring-shaped conformation that was similar to the cryo-EM map, suggesting the accuracy of this predicted model. Therefore, we used a structure modelling strategy to investigate the molecular interaction between USP24 and USP24 inhibitors. The binding site of the USP24 model was identified according to the structural alignment with USP7 complexed with its inhibitor (Fig. 7B(a)). Compound 677397 (USP24-i) was prepared by protonation in aqueous solution. Molecular docking was performed using LeadIT and generated poses for analysis. The score for binding affinity between USP24 and USP24-i analogues was estimated by using a hybrid enthalpy/entropy approach with default scoring parameters. We calculated the docking score for a series of USP24-i analogues obtained by using AI deep learning in previous study. The results indicated that only one analogue, 677-8 (USP24-i-101), had higher binding affinity with the catalytic motif of USP24 than NCI677397 (Fig. 7B(d)). The docking posture shows that the catalytic triad of USP24 interacts with 677-8 through hydrogen bonds, which may result in blocking its hydrolytic activity (Fig. 7B(c)). Therefore, we further examined the potency of this optimized USP24-i analogue with a cytotoxicity assay. The USP24 inhibitors, 677397 (USP24-i) and 677-08 (USP24-i-101), were used to study the cytotoxic effect of Taxol on A549-T24 cells (Fig. 7C). The data indicated that USP24-i-101 is better than USP24-i in the inhibition of drug resistance in Taxol-induced drug resistant A549 cells (A549-T24) (Fig. 7D(a)) and gefitinib-induced drug resistant PC9 cells (PC9-GR) (Fig. 7D(a)) through a synergistic effect manner (CI < 1, Fig. 7D(b)).

**Fig. 8:**
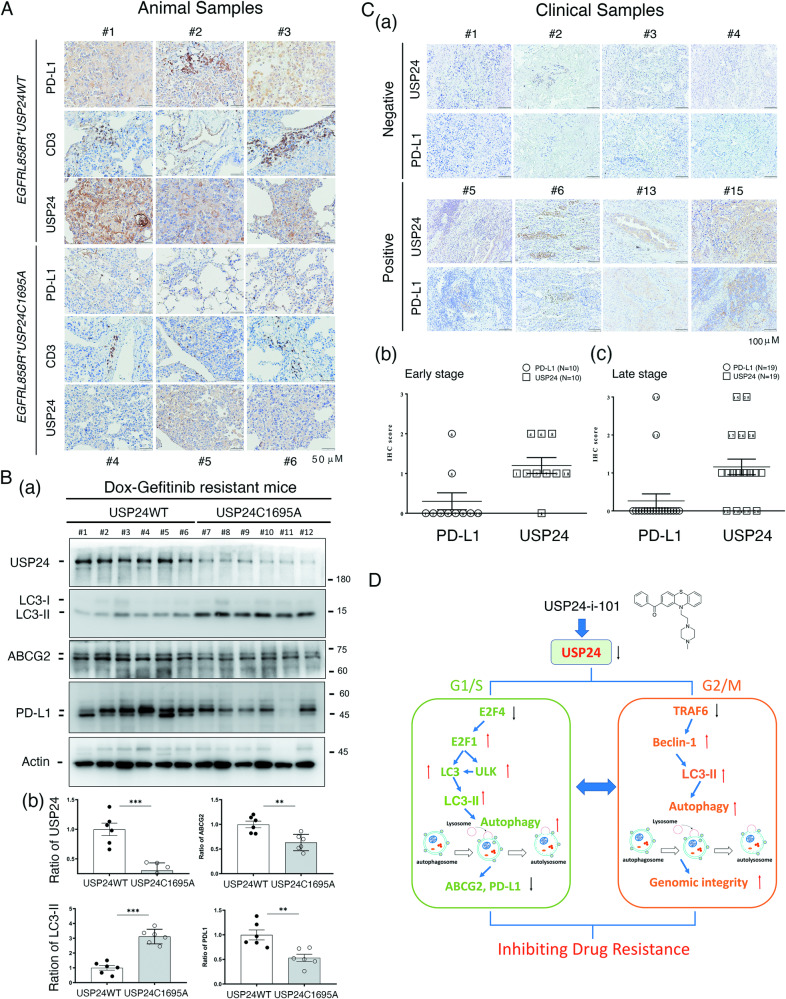
The positive correlation between the levels of USP24 and PD-L1 in animal and clinical specimens. The relevance among prognosis and several related proteins, including E2F4, E2F1, LC3-II, ULK1, ABCG2, PD-L1 and CD3 in *EGFR*^L858R^*USP24^*WT*^ and *EGFR*^L858R^*USP24^*C1695A*^ drug resistant mice was studied by IHC (**A**) and IB (**B(a)**), and then the levels of the indicated protein were quantitated after three independent experiments (B(b)). **C** The levels of PD-L1 and USP24 in clinical lung cancer patients were studied by IHC with anti-PD-L1 and anti-USP24 antibodies (a), and the correlation of USP24 levels and PD-L1 levels in early-stage of lung cancer (b), and late-stage of lung cancer (c) was show here. **D** The working model of how targeting USP24 prevents drug resistance during lung cancer therapy. The statistical analysis was performed by one way ANOVA; **p < 0.01, ***p < 0.005.

Next, we used USP24-i-101 to study the effect of targeting USP24 on drug-resistant *EGFR*^*L858R*^ mice with lung cancer induced by gefitinib treatment for a long time (217 days) (Supplementary Fig. 6, Fig. 7E and Fig. 8D). Body weight and lung micro-CT were used to monitor the effect of gefitinib during therapy (Supplementary Fig. 6A, Supplementary Fig. 6B, Fig. 7D). After short-term gefitinib treatment, the body weight of the mice was increased, and the tumor signal of micro-CT in the lung organs was nearly abolished compared to that of the mice with doxycycline-induced lung cancer (Supplementary Fig. 6A,  B and Fig. 7D), suggesting that gefitinib was able to kill tumor cells at the initiation of treatment. After treatment for a long time (217th day), body weight of the mice treated with gefitinib only did not increase again (green arrow), and the tumor signal of lung micro-CT in the gefitinib-treated mice was slightly higher than that with the cocktail treatment of gefitinib and USP24-i-101 (Supplementary Fig. 6A, Supplementary Fig. 6B and Fig. 7D (b)). After sacrificing these mice, we found that the tumor area in the gefitinib-treated mice was slightly greater than that in the gefitinib/USP24-i-101-treated mice, implying that USP24-i-101 treatment may inhibit or delay the drug resistance during gefitinib treatment (Fig. 7E (a), Fig. 7E(b) and Fig. 7E(c)). In addition, the LC3-II levels in the gefitinib/USP24-i-101-treated mice was higher than that in the gefitinib-treated mice, implying that targeting USP24-mediated activation of autophagy may be involved in inhibiting drug resistance in vitro and in vivo (Fig. 7E (d)). Several related proteins, PD-L1, ABCG2, USP24 and CD3, were decreased, but LC3-II was increased in the *EGFR*^L858R^*USP24^*C1695A*^ mice (Fig. 8A and Fig. 8B). The levels of USP24 and PD-L1 in lung tumors of lung cancer patients indicated that a partially positive correlation between USP24 and PD-L1 (Fig. 8C). High PD-L1 expression was found only in 4 cases of 29 lung cancer patients (13.8%) (Fig. 8C(b) and Fig. 8C(c)), and all the 4 patients with higher PD-L1 expression had higher USP24 expression (Fig. 8C(b) and Fig. 8C(c)). However, many cases with higher USP24 expression still have low PD-L1 expression, indicating that other unknow factor(s) are also essential for the regulation of PD-L1 during lung cancer progression.

## Discussion

Targeting USP24 by USP24-i-101 induces autophagy in interphase and mitosis by inhibiting E2F4 and TRAF6, respectively, thereby inhibiting the expression of ABCG2 and PD-L1 and maintaining genomic integrity, resulting in the inhibition of drug resistance acquired by gefitinib- or Taxol-treatment of lung cancer (Fig. 8D).

In this study, we found that targeting USP24 by USP24-i-101, silencing USP24 by shRNA-USP24 or knockout by CRISPR-Cas9 can induce autophagy in vitro and in vivo. After analysis of all Atg proteins in the USP24-i-treated cells, we found no significant difference. However, we found that the ULK and LC3 gene expression was regulated by USP24-mediated E2F4 and E2F1 in interphase, and beclin-1 levels were increased by a decrease in TRAF6 levels in mitosis. USP24 has been reported to inhibit autophagy in neurons by increasing the degradation of ULK1 to repress the human induced-pluripotent stem cell (iPSC) differentiation into dopaminergic neurons, which is involved in Parkinson’s disease (PD) [35]. In this study, we found that targeting USP24 by USP24-i-101 can dramatically induce autophagy through upregulation of ULK1, LC3 and ULK phosphorylation at Ser555 in lung cancer to negatively regulate drug resistance. Because autophagic inhibition was found in most neurodegenerative diseases, such as Parkinson’s disease (PD) and Alzheimer’s disease (AD), the specific USP24 inhibitor, USP24-i-101, might be also work to prevent PD and AD, which needs to be clarified in the future [36]. In addition, we found that USP24-i treatment dramatically induced autophagy, but only slightly activated autophagy through silencing USP24 by shRNA. One possibility is that USP24-i-101 treatment can rapidly inhibit all USP24 activity, but this activity is only partially silenced by shRNA, and this process requires 2-3 days. The other possibility is that USP24-i-101 not only inhibits USP24 but also other similar USPs, which induce polyubiquitin signaling, thereby dramatically inducing autophagy [37]. According to previous studies, autophagy is induced under stress conditions such as starvation [38]. Several kinds of autophagy have been found to respond to different stress conditions [39], including macroautophagy, microautophagy, lipophagy, chaperone-dependent autophagy and ubiquitin-dependent autophagy [40]. Because USP24 is a deubiquitinase, targeting USP24-induced autophagy might be ubiquitin-mediated autophagy. According to the previous studies, several USPs are also involved in autophagy [21]. USP20 induces autophagy by stabilizing ULK1 [41]. In addition, USP19 induces autophagy through deubiquitination of beclin1 [42]. USP10 and USP13 can deubiquitinate polyubiquitinated beclin-1 to induce autophagy [43]. USP8 keeps Parkin, a VDAC1 E3 ligase, in its active form to induce mitophagy [44, 45]. USP15, USP30 and USP35 inhibit mitophagy by removing the ubiquitin signal from Parkin targets [46–48]. USP10 also stabilizes p53 to elicit a balanced breakdown of beclin1, resulting in the inhibition of autophagy [49]. Most of previous studies indicate that appropriate autophagy is related to pro-survival response, but excessive autophagy and impaired lysosomal activity may be involved in autophagy-dependent ferroptosis [50–52]. In addition, p62/SQSTM1 is not only involved in autophagy, but also involved in select autophagy-mediated lipid peroxidation, thereby inducing ferroptosis [53, 54]. In this study, we found that the signal of autophagy by USP24-i was higher than that by rapamycin. In addition, the level of p62/SQSTM1 was increased, not decreased, by USP24-i treatment, implying USP24-i targeting USP24 inhibits drug resistance might be through inducing p62/SQSTM1-mediated select autophagy-dependent ferroptosis. However, the detail mechanism of how USP24 involved in select autophagy needs to be clarified in the future. Our previous study also revealed that USP24 can stabilize p53 in lung cancer cells [55], which is consistent with the induction of autophagy by targeting USP24 with USP24-i-101. Most of studies indicate that autophagy is induced in interphase in response to stress conditions, and only a few reports have revealed that autophagy is also induced during mitosis [56]. In this study, we found that USP24 downregulation in mitosis might be related to the induction of autophagy by destabilizing TRAF6, which is the E3 ligase of beclin-1, thereby maintaining genomic integrity. LC3-I and LC3-II were significantly increased in mitosis, implying that the enzyme activities of ATG4 and ATG7 might be increased during the mitotic period, According to previous studies, ATG4 phosphorylation at Ser383 and Ser392 could activate these enzymes [57]. In addition, the interaction between ATG7 and ATG3 can activate ATG7 [58]. The interaction and post-translational modification of ATG4 and ATG7 during mitosis will be studied in the future. According to the previous studies, most organelles will be repurposed to allow the correct distribution of all chromosomes during mitosis [59]. Most studies on the role of autophagy in mitosis have focused on genomic integrity. The role of autophagy during mitosis is diverse. Some studies indicate that autophagic induction during mitosis promotes genomic instability, but others reveal that it can maintain the genome integrity [60, 61]. Based on the different situations, the role of autophagy may be different. In addition, CDK1 and AMPK have been reported to be involved in triggering autophagy in mitosis [62], but the detailed signaling pathways of autophagic induction during mitosis need to be clarified in the future.

Many factors, such as genomic instability and ABC transporters, cause drug resistance [63], which is currently a clinical emergency issue. A number of previous studies on the relationship between drug resistance and autophagy have been performed [64]. Some studies support that autophagy positively regulates drug resistance; others suggest that it inhibits drug resistance [65]. In this study, targeting USP24 induced autophagy was essential for blocking drug resistance acquired by Taxol or gefitinib treatment in vitro and in vivo through a decrease in ABCG2 levels and genomic instability, which can inhibit drug pumping out of cells and clonal selection for drug resistance [3]. We not only studied the role of USP24 in cancer cells, also studied it by using lung cancer animal model, *EGFR*^*L858R*^, treated with gefitinib and USP24-i-101 for a long time. In addition, we used USP24 knockout mice, *USP24*^C1695A^*EGFR^*L858R*^, to study the role of USP24 in drug resistance acquired by gefitinib. All the data indicated that USP24 may positively regulated drug resistance acquired by gefitinib treatment. However, whether USP24-i-101 is also effective on other genomic types of lung cancer, such as *Kras*^*G12D*^ or other cancer types in vivo, needs to be clarified in the future. In addition, we used gefitinib to induce drug resistance in mice to evaluate the effect of targeting USP24 on blocking drug resistance. USP24-i-101 may inhibit drug resistance acquired from other targeted therapy drugs, such as erlotinib, osimertinib, and afatinib, which needs to be addressed in the future.

In this study, we found that targeting USP24 by USP24-i-101 can overcome Taxol- or gefitinib-induced drug resistance in lung cancer. In addition, we found that USP24-i-101-induced autophagy is essential for blocking drug resistance. We also studied the level of the apoptosis marker cleaved caspase 3 in autophagy-dependent cell death. However, the level of the cleaved form of caspase 3 was not changed, indicating that it does not occur through apoptosis. Several pathways are involved in autophagy-mediated cell death [66]. First, autophagy only accompanies the cell death process but is not involved in the cell death pathway. Second, autophagy induction triggers apoptosis. Finally, autophagy leads to cell death through a distinct mechanism that is triggered independently of apoptosis [67]. In this study, targeting USP24-induced autophagy caused cell death, which could be inhibited by bafilomycin A1 or chloroquine treatment. In addition, there was no change in the caspase 3 cleavage level. Based on these results, targeting USP24-induced cell death occurs in an autophagy-dependent but apoptosis-independent manner. The detailed cell death signal pathway(s) triggered by targeting USP24 need to be clarified in the future.

In addition to the gene expression involved in lung cancer progression, other genes related to other pathways involved in hematopoietic disease, dermatomyositis, leukocyte migration/cell-cell adhesion/chemotaxis and fat cell differentiation might also be related to USP24-mediated drug resistance. Compared to most of the solid cancers, hematopoietic malignancies have more abnormalities in T-cell development and differentiation [68]. Our previous studies indicated that USP24 in macrophages can regulate the tumor microenvironment to enhance lung cancer progression [27], indicating that USP24 might also be critical for the regulation of T-cell development and differentiation. The detailed mechanism by which USP24 regulates T cells needs to be addressed in the future. In addition, many genes related to cellular adhesion, migration and chemotaxis were regulated by USP24, which might be related to the ECM around tumors. In addition, as shown in Fig. 5D, we found that collagen was downregulated in *EGFR*^L858R^*USP24^*C1695A*^ drug-resistant mice with lung cancer, which is also related to the ECM. Our recent studies also indicated that USP24 can regulate CD44, which is also involved in the ECM of lung cancer [3]. Thus, USP24 may be not only important inside cancer cells but also critical in other cells around cancer cells. USP24 in other cells might also be important for lung cancer drug resistance. Recently, immune therapy has been used in cancer treatment. PD-1 in T cells can bind with PD-L1 in cancer cells, leading to the inhibition of T cells [69]. Therefore, anti-PD1 or anti-PD-L1 antibodies can block the interaction between T cells and cancer cells, subsequently inducing an immune response and resulting in cancer prevention [70]. Several studies have reported that the overexpression of PD-L1 in cancer cells is related to drug resistance [71]. In this study, we found that PD-L1 was highly expressed in drug resistant mice, and also highly expressed in PC9-GR cells, indicating that PD-L1 might be involved in drug resistance through inactivation of T_reg_ cells. In addition, previous studies have indicated that autophagy is related to PD-L1 levels [72]. In this study, targeting USP24-induced autophagy was related to PD-L1 degradation in cancer cells. In summary, targeting USP24 not only regulates cancer cells themselves but also other cells around cancer cells to inhibit drug resistance acquired by cancer therapy.

### Reporting summary

Further information on research design is available in the Nature Research Reporting Summary linked to this article.

### Supplementary information

SUPPLEMENTARY FIGURES

Suppl. Fig4. GFP-USP24 overexpression in lung cancer cells causes uneven cytokinesis.-MOV

Supplementary- All Raw Western Files

Reporting Summary

## Data Availability

The datasets generated and/or analyzed during the current study are available from the corresponding author on reasonable request.

## References

[1] Konieczkowski DJ, Johannessen CM, Garraway LA (2018). A convergence-based framework for cancer drug resistance. Cancer Cell.

[2] Roberts AG (2021). The structure and mechanism of drug transporters. Methods Mol Biol.

[3] Wang SA, Young MJ, Wang YC, Chen SH, Liu CY, Lo YA (2021). USP24 promotes drug resistance during cancer therapy. Cell Death Differ.

[4] Young MJ, Hsu KC, Lin TE, Chang WC, Hung JJ (2019). The role of ubiquitin-specific peptidases in cancer progression. J Biomed Sci.

[5] Reyes-Turcu FE, Ventii KH, Wilkinson KD (2009). Regulation and cellular roles of ubiquitin-specific deubiquitinating enzymes. Annu Rev Biochem.

[6] Deng L, Meng T, Chen L, Wei W, Wang P (2020). The role of ubiquitination in tumorigenesis and targeted drug discovery. Signal Transduct Target Ther.

[7] Senft D, Qi J, Ronai ZA (2018). Ubiquitin ligases in oncogenic transformation and cancer therapy. Nat Rev Cancer.

[8] Wang YC, Wang SA, Chen PH, Hsu TI, Yang WB, Chuang YP (2016). Variants of ubiquitin-specific peptidase 24 play a crucial role in lung cancer malignancy. Oncogene.

[9] Debnath J, Gammoh N, Ryan KM. Autophagy and autophagy-related pathways in cancer. Nat Rev Mol Cell Biol. 2023;24:1–16. 10.1038/s41580-023-00585-z PMC9980873 36864290

[10] He C, Klionsky DJ (2009). Regulation mechanisms and signaling pathways of autophagy. Annu Rev Genet.

[11] Parzych KR, Klionsky DJ (2014). An overview of autophagy: morphology, mechanism, and regulation. Antioxid Redox Signal.

[12] Lu G, Wang Y, Shi Y, Zhang Z, Huang C, He W (2022). Autophagy in health and disease: from molecular mechanisms to therapeutic target. MedComm (2020).

[13] Lim SM, Mohamad Hanif EA, Chin SF (2021). Is targeting autophagy mechanism in cancer a good approach? The possible double-edge sword effect. Cell Biosci.

[14] Muriach M, Flores-Bellver M, Romero FJ, Barcia JM (2014). Diabetes and the brain: oxidative stress, inflammation, and autophagy. Oxid Med Cell Longev.

[15] Rubinsztein DC, Codogno P, Levine B (2012). Autophagy modulation as a potential therapeutic target for diverse diseases. Nat Rev Drug Discov.

[16] Yin Z, Popelka H, Lei Y, Yang Y, Klionsky DJ (2020). The roles of ubiquitin in mediating autophagy. Cells.

[17] Yun CW, Lee SH (2018). The roles of autophagy in cancer. Int J Mol Sci.

[18] Oh SJ, Lee MS (2022). Role of autophagy in the pathogenesis of diabetes and therapeutic potential of autophagy modulators in the treatment of diabetes and metabolic syndrome. J Korean Med Sci.

[19] Klionsky DJ, Petroni G, Amaravadi RK, Baehrecke EH, Ballabio A, Boya P (2021). Autophagy in major human diseases. EMBO J.

[20] Ghavami S, Shojaei S, Yeganeh B, Ande SR, Jangamreddy JR, Mehrpour M (2014). Autophagy and apoptosis dysfunction in neurodegenerative disorders. Prog Neurobiol.

[21] Chen RH, Chen YH, Huang TY (2019). Ubiquitin-mediated regulation of autophagy. J Biomed Sci.

[22] Kocaturk NM, Gozuacik D (2018). Crosstalk between mammalian autophagy and the ubiquitin-proteasome system. Front Cell Dev Biol.

[23] Nakatogawa H (2013). Two ubiquitin-like conjugation systems that mediate membrane formation during autophagy. Essays Biochem.

[24] Nitta A, Hori K, Tanida I, Igarashi A, Deyama Y, Ueno T (2019). Blocking LC3 lipidation and ATG12 conjugation reactions by ATG7 mutant protein containing C572S. Biochem Biophys Res Commun.

[25] Otomo C, Metlagel Z, Takaesu G, Otomo T (2013). Structure of the human ATG12~ATG5 conjugate required for LC3 lipidation in autophagy. Nat Struct Mol Biol.

[26] Grumati P, Dikic I (2018). Ubiquitin signaling and autophagy. J Biol Chem.

[27] Wang YC, Wu YS, Hung CY, Wang SA, Young MJ, Hsu TI (2018). USP24 induces IL-6 in tumor-associated microenvironment by stabilizing p300 and beta-TrCP and promotes cancer malignancy. Nat Commun.

[28] Punjani A, Rubinstein JL, Fleet DJ, Brubaker MA (2017). cryoSPARC: algorithms for rapid unsupervised cryo-EM structure determination. Nat Methods.

[29] Kimura S, Noda T, Yoshimori T (2007). Dissection of the autophagosome maturation process by a novel reporter protein, tandem fluorescent-tagged LC3. Autophagy.

[30] Karabiyik C, Vicinanza M, Son SM, Rubinsztein DC (2021). Glucose starvation induces autophagy via ULK1-mediated activation of PIKfyve in an AMPK-dependent manner. Dev Cell.

[31] Cui J, Ogasawara Y, Kurata I, Matoba K, Fujioka Y, Noda NN (2022). Targeting the ATG5-ATG16L1 protein-protein interaction with a hydrocarbon-stapled peptide derived from ATG16L1 for autophagy inhibition. J Am Chem Soc.

[32] Wang SA, Wang YC, Chuang YP, Huang YH, Su WC, Chang WC (2017). EGF-mediated inhibition of ubiquitin-specific peptidase 24 expression has a crucial role in tumorigenesis. Oncogene.

[33] Klionsky DJ, Abdel-Aziz AK, Abdelfatah S, Abdellatif M, Abdoli A, Abel S (2021). Guidelines for the use and interpretation of assays for monitoring autophagy (4th edition)(1). Autophagy.

[34] Politi K, Zakowski MF, Fan PD, Schonfeld EA, Pao W, Varmus HE (2006). Lung adenocarcinomas induced in mice by mutant EGF receptors found in human lung cancers respond to a tyrosine kinase inhibitor or to down-regulation of the receptors. Genes Dev.

[35] Thayer JA, Awad O, Hegdekar N, Sarkar C, Tesfay H, Burt C (2020). The PARK10 gene USP24 is a negative regulator of autophagy and ULK1 protein stability. Autophagy.

[36] Guo F, Liu X, Cai H, Le W (2018). Autophagy in neurodegenerative diseases: pathogenesis and therapy. Brain Pathol.

[37] Yuan T, Yan F, Ying M, Cao J, He Q, Zhu H (2018). Inhibition of ubiquitin-specific proteases as a novel anticancer therapeutic strategy. Front Pharmacol.

[38] Mizushima N (2007). Autophagy: process and function. Genes Dev.

[39] Kroemer G, Marino G, Levine B (2010). Autophagy and the integrated stress response. Mol Cell.

[40] Trelford CB, Di, Guglielmo GM (2021). Molecular mechanisms of mammalian autophagy. Biochem J.

[41] Kim JH, Seo D, Kim SJ, Choi DW, Park JS, Ha J (2018). The deubiquitinating enzyme USP20 stabilizes ULK1 and promotes autophagy initiation. EMBO Rep.

[42] Jin S, Tian S, Chen Y, Zhang C, Xie W, Xia X (2016). USP19 modulates autophagy and antiviral immune responses by deubiquitinating Beclin-1. EMBO J.

[43] Liu J, Xia H, Kim M, Xu L, Li Y, Zhang L (2011). Beclin1 controls the levels of p53 by regulating the deubiquitination activity of USP10 and USP13. Cell.

[44] Durcan TM, Tang MY, Perusse JR, Dashti EA, Aguileta MA, McLelland GL (2014). USP8 regulates mitophagy by removing K6-linked ubiquitin conjugates from parkin. EMBO J.

[45] Durcan TM, Fon EA (2015). The three ‘P’s of mitophagy: PARKIN, PINK1, and post-translational modifications. Genes Dev.

[46] Wang Y, Serricchio M, Jauregui M, Shanbhag R, Stoltz T, Di Paolo CT (2015). Deubiquitinating enzymes regulate PARK2-mediated mitophagy. Autophagy.

[47] Magraoui FE, Reidick C, Meyer HE, Platta HW (2015). Autophagy-related deubiquitinating enzymes involved in health and disease. Cells.

[48] Cornelissen T, Haddad D, Wauters F, Van Humbeeck C, Mandemakers W, Koentjoro B (2014). The deubiquitinase USP15 antagonizes Parkin-mediated mitochondrial ubiquitination and mitophagy. Hum Mol Genet.

[49] Yuan J, Luo K, Zhang L, Cheville JC, Lou Z (2010). USP10 regulates p53 localization and stability by deubiquitinating p53. Cell.

[50] Tang D, Chen X, Kang R, Kroemer G (2021). Ferroptosis: molecular mechanisms and health implications. Cell Res.

[51] Liu J, Kuang F, Kroemer G, Klionsky DJ, Kang R, Tang D (2020). Autophagy-dependent ferroptosis: machinery and regulation. Cell Chem Biol.

[52] Zhou B, Liu J, Kang R, Klionsky DJ, Kroemer G, Tang D (2020). Ferroptosis is a type of autophagy-dependent cell death. Semin Cancer Biol.

[53] Wu Y, Zhang S, Gong X, Tam S, Xiao D, Liu S (2020). The epigenetic regulators and metabolic changes in ferroptosis-associated cancer progression. Mol Cancer.

[54] Chen F, Cai X, Kang R, Liu J, Tang D (2023). Autophagy-dependent ferroptosis in cancer. Antioxid Redox Signal.

[55] Wang SA, Young MJ, Jeng WY, Liu CY, Hung JJ (2020). USP24 stabilizes bromodomain containing proteins to promote lung cancer malignancy. Sci Rep.

[56] Li Z, Ji X, Wang D, Liu J, Zhang X (2016). Autophagic flux is highly active in early mitosis and differentially regulated throughout the cell cycle. Oncotarget.

[57] Ni Z, He J, Wu Y, Hu C, Dai X, Yan X (2018). AKT-mediated phosphorylation of ATG4B impairs mitochondrial activity and enhances the Warburg effect in hepatocellular carcinoma cells. Autophagy.

[58] Taherbhoy AM, Tait SW, Kaiser SE, Williams AH, Deng A, Nourse A (2011). Atg8 transfer from Atg7 to Atg3: a distinctive E1-E2 architecture and mechanism in the autophagy pathway. Mol Cell.

[59] Almacellas E, Mauvezin C (2022). Emerging roles of mitotic autophagy. J Cell Sci.

[60] Matsui A, Kamada Y, Matsuura A (2013). The role of autophagy in genome stability through suppression of abnormal mitosis under starvation. PLoS Genet.

[61] Vessoni AT, Filippi-Chiela EC, Menck CF, Lenz G (2013). Autophagy and genomic integrity. Cell Death Differ.

[62] Li Z, Zhang X (2017). Kinases involved in both autophagy and mitosis. Int J Mol Sci.

[63] Patel H, Wu ZX, Chen Y, Bo L, Chen ZS (2021). Drug resistance: from bacteria to cancer. Mol Biomed.

[64] Chang H, Zou Z (2020). Targeting autophagy to overcome drug resistance: further developments. J Hematol Oncol.

[65] Long W, Zhang L, Wang Y, Xie H, Wang L, Yu H (2022). Research progress and prospects of autophagy in the mechanism of multidrug resistance in tumors. J Oncol.

[66] Denton D, Kumar S (2019). Autophagy-dependent cell death. Cell Death Differ.

[67] Noguchi M, Hirata N, Tanaka T, Suizu F, Nakajima H, Chiorini JA (2020). Autophagy as a modulator of cell death machinery. Cell Death Dis.

[68] Montironi C, Munoz-Pinedo C, Eldering E (2021). Hematopoietic versus solid cancers and T cell dysfunction: looking for similarities and distinctions. Cancers.

[69] Han Y, Liu D, Li L (2020). PD-1/PD-L1 pathway: current researches in cancer. Am J Cancer Res.

[70] Cha JH, Chan LC, Li CW, Hsu JL, Hung MC (2019). Mechanisms controlling PD-L1 expression in cancer. Mol Cell.

[71] Lei Q, Wang D, Sun K, Wang L, Zhang Y (2020). Resistance mechanisms of Anti-PD1/PDL1 therapy in solid tumors. Front Cell Dev Biol.

[72] Gao L, Chen Y (2021). Autophagy controls programmed death-ligand 1 expression on cancer cells (Review). Biomed Rep.

