# Supplementary material for: USP24-i-101 targeting of USP24 activates autophagy to inhibit drug resistance acquired during cancer therapy
Source: Cell Death Differ. 2024 Mar 15;31(5):574–91. doi: 10.1038/s41418-024-01277-7 (PMC11093971; doi:10.1038/s41418-024-01277-7)
Supplement: Supplementary file 1 — SUPPLEMENTARY FIGURES [file 41418_2024_1277_MOESM1_ESM.docx]

**SUPPLEMENTARY FIGURES**

**
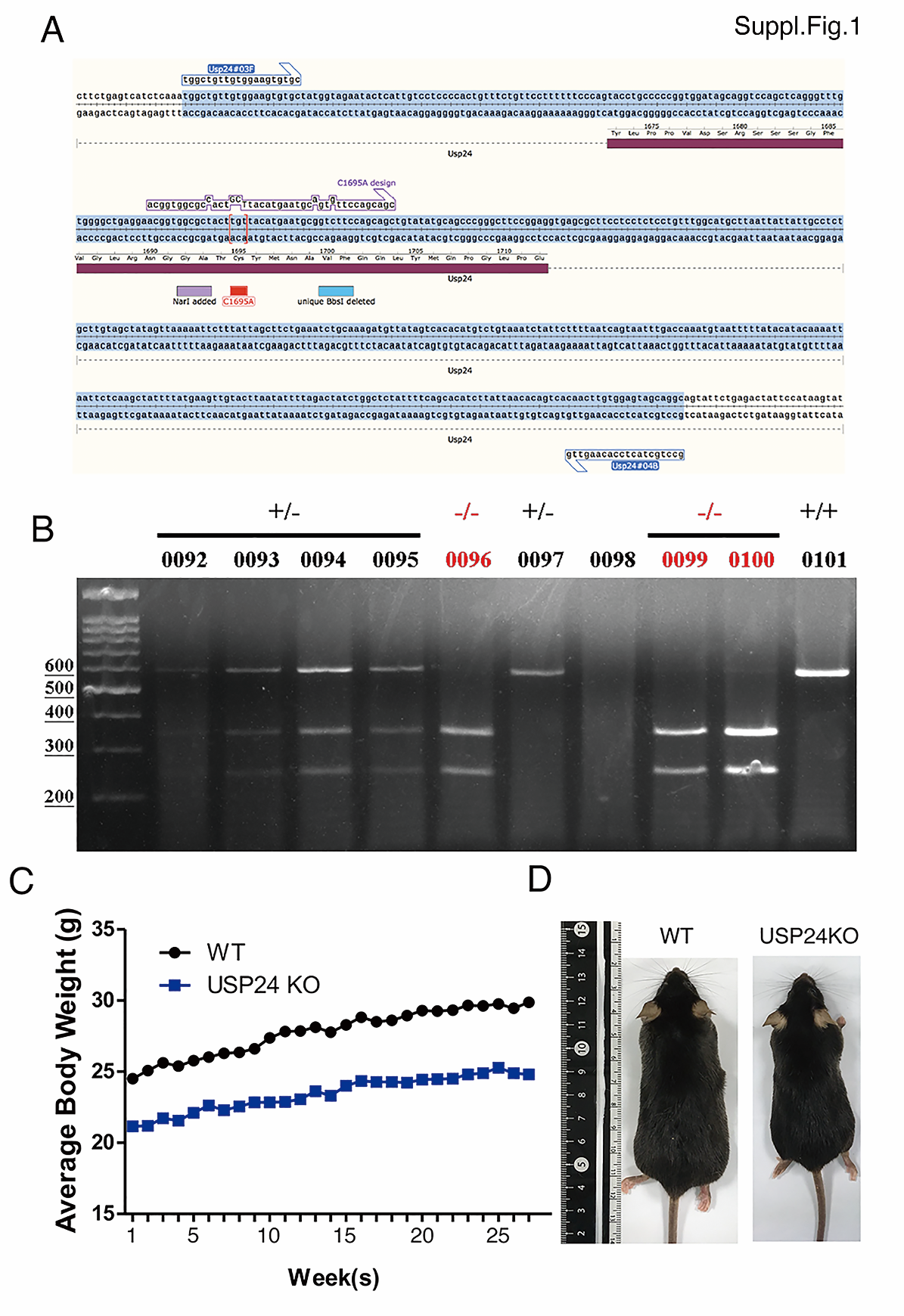
**

**Suppl. Fig. 1.** Functional knockout of USP24 was constructed by CRISPR-Cas9. (A). The *USP24* gene was edited to mutate 1695^th^ amino acid, Cysteine, to Alanine (C1695A) by CRISPR-Cas9 and constructed to be functional knockout of USP24, *USP24^C1695A^*, in B6 mice. A new restriction site, NarI, was added for genotyping (B). The body weight (C) and size (D) of wildtype and *USP24^C1695A^* mice were show here.


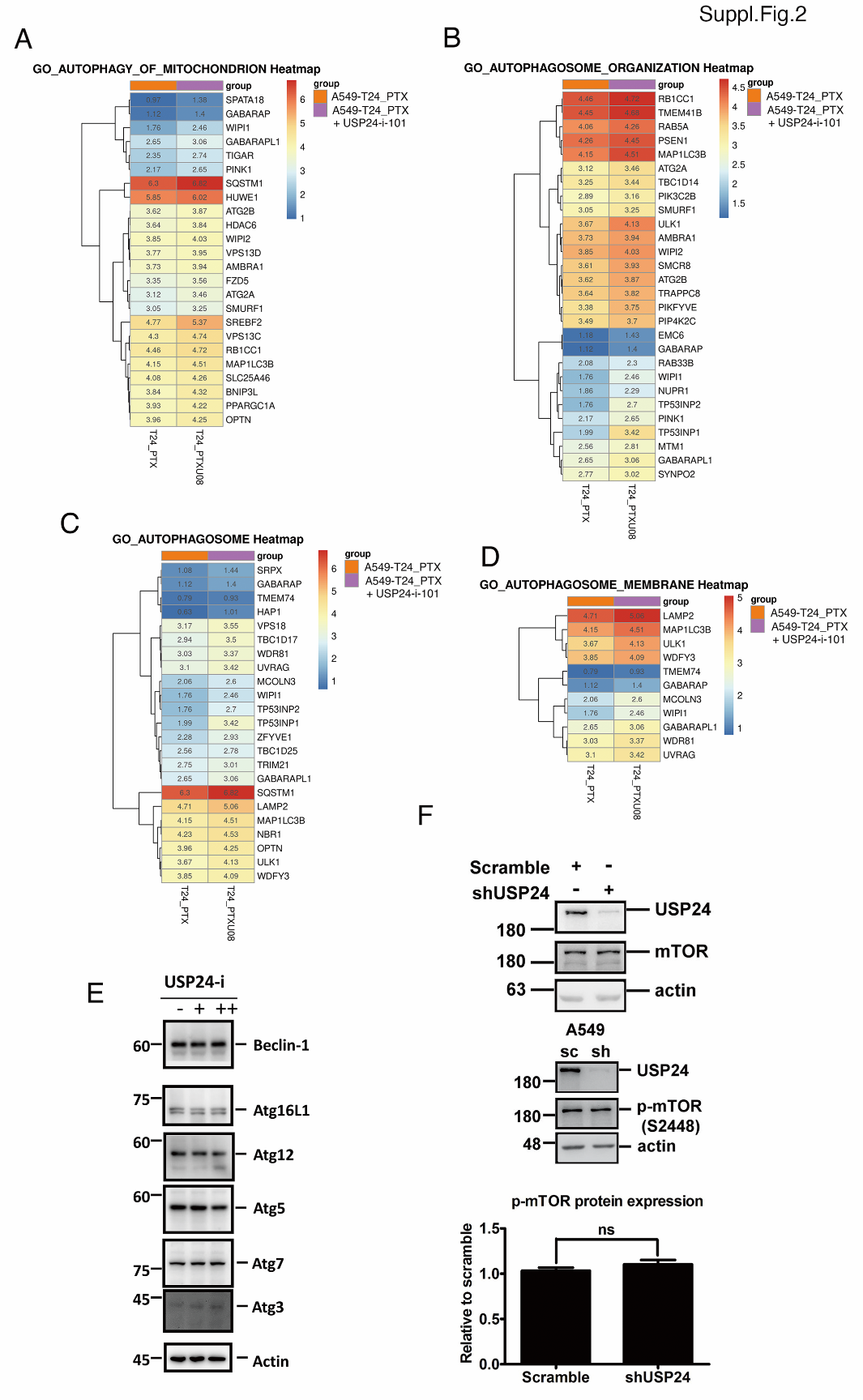


**Suppl. Fig. 2.** Targeting USP24 induces autophagy related genes expression. (A-D). A549 drug resistant cell line (A549-T24) was treated with Taxol and 5 μM USP24-i-101. The mRNA was isolated to study the global genes expression by RNA-seq. All the autophagy related genes were show here. (E). A549-T24 cells were treated with 5 μM USP24-i-101 for 24 h, and the levels of autophagy related proteins were studied by IB. (F). USP24 was knocked down in A549 cells, and the levels of mTOR, p-mTOR and USP24 were studied by IB. After three independent experiments, the level of p-mTOR was quantitated, and statistical analysis was performed by a t test, ns: nonspecific.


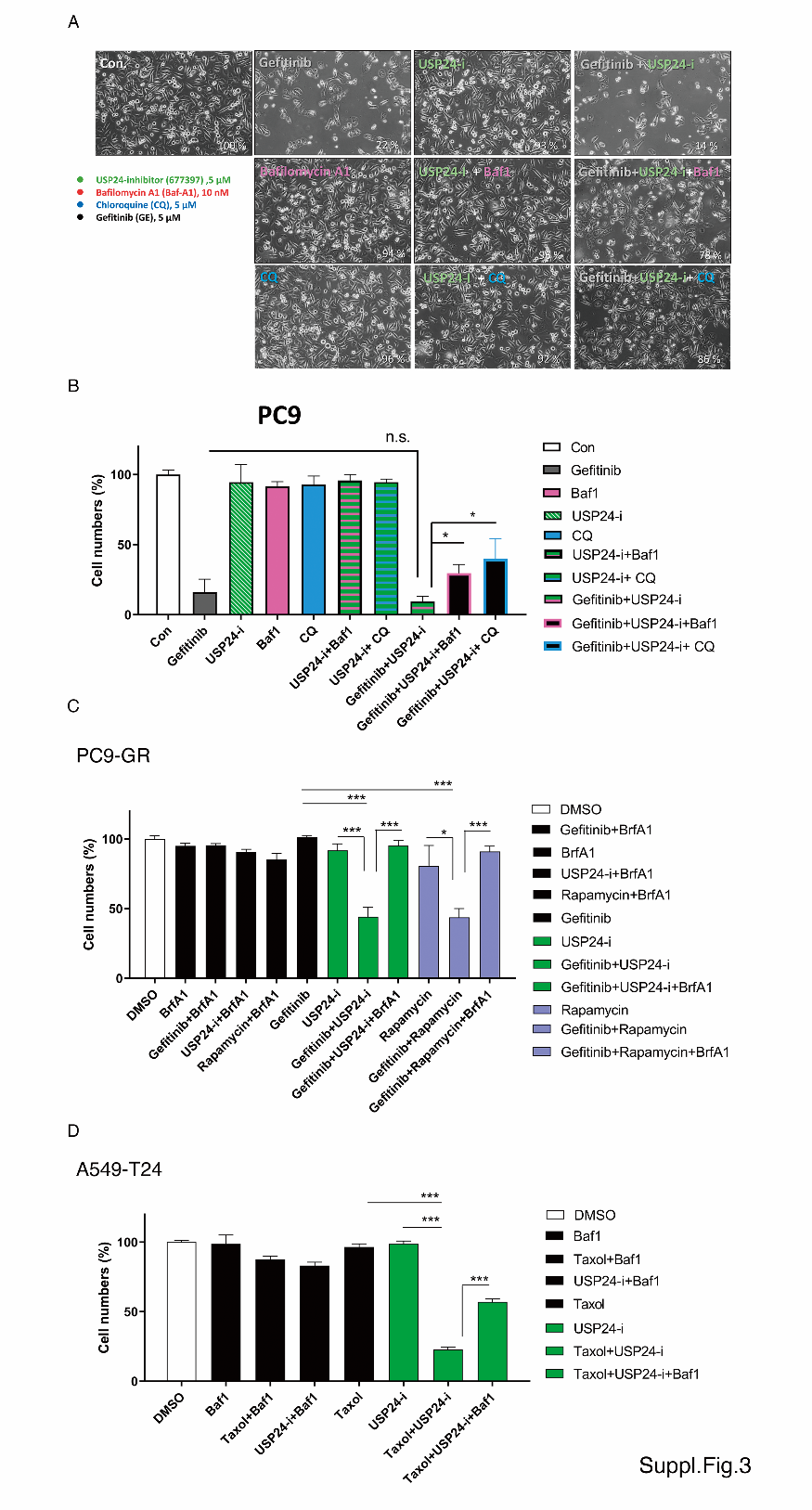


**Suppl. Fig. 3.** Targeting USP24-induced autophagy is critical for blocking gefitinib-induced drug resistance. PC9 cells were treated with 5 μM gefitinib and 5 μM USP24-i-101 with or without 5 μM chloroquine (CQ) or 10 μM bafilomycin A1 (Baf-A1) treatment for 24 h. The morphology (A) and cell number (B) were show here and statistical analysis was performed by a t test, *p<0.05; n.s. = nonspecific. USP24-i and rapamycin induced autophagy are required for blocking gefitinib- and Taxol-induced drug resistance in lung cancer cells. PC9-GR (C) and A549-T24 (D) were treated with 5 μM gefitinib or Taxol respectively with or without 10 nM Baf-A1, 5 μM USP24-i or 2.5 μM rapamycin treatment for 24 h, cell number were counted, and statistical analysis was performed by a t test, *p<0.05, ***p<0.005.


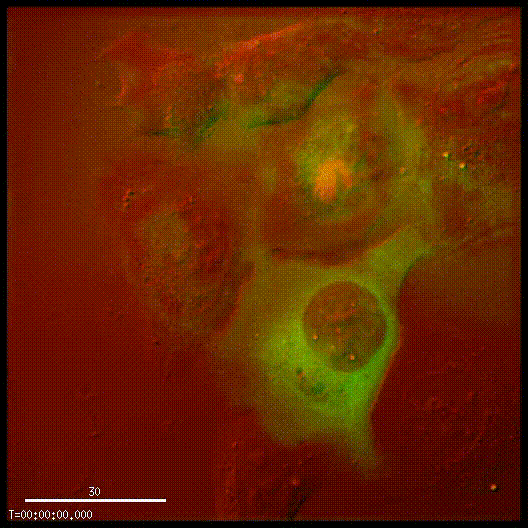


**Suppl. Fig. 4.** Overexpression of GFP-USP24 induces the unequal segregation during mitosis. GFP-USP24 was overexpression in A549 cells for 24 h, and then recorded the cells with real time immunofluorescence microscope for 36 h.


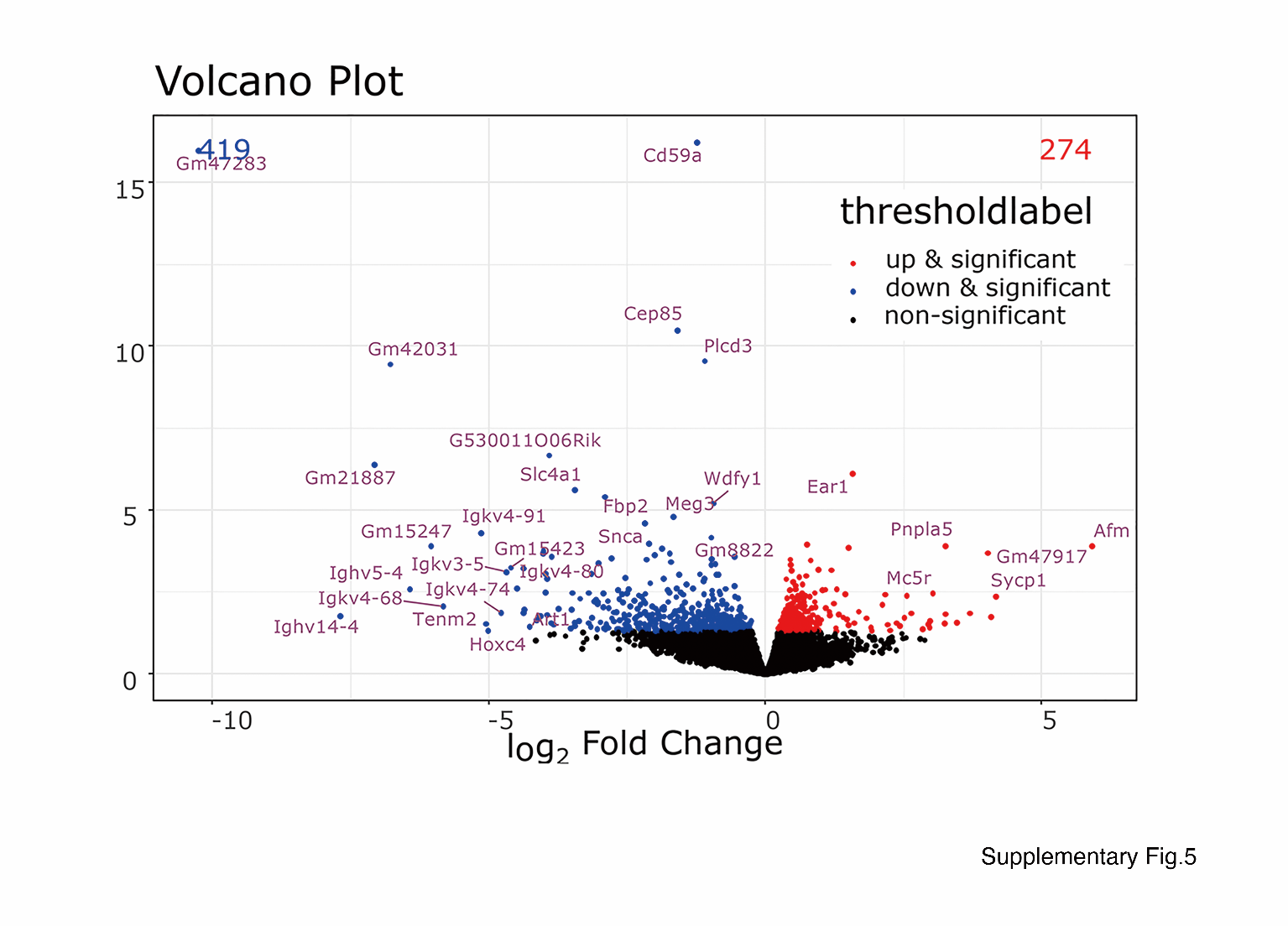


**Suppl. Fig. 5.** Volcano plot analysis of USP24 regulates gene expression involved in lung cancer progression and drug resistance during drug resistance in vivo. Total RNA specimens were isolated from the lungs of gefitinib-induced *EGFR^L858R^*USP24^WT^* and *EGFR^L858R^*USP24^C1695A^* drug resistant mice to study the gene expression profile by RNA-Seq (*EGFR^L858R^*USP24^C1695A^/ EGFR^L858R^*USP24^WT^*).


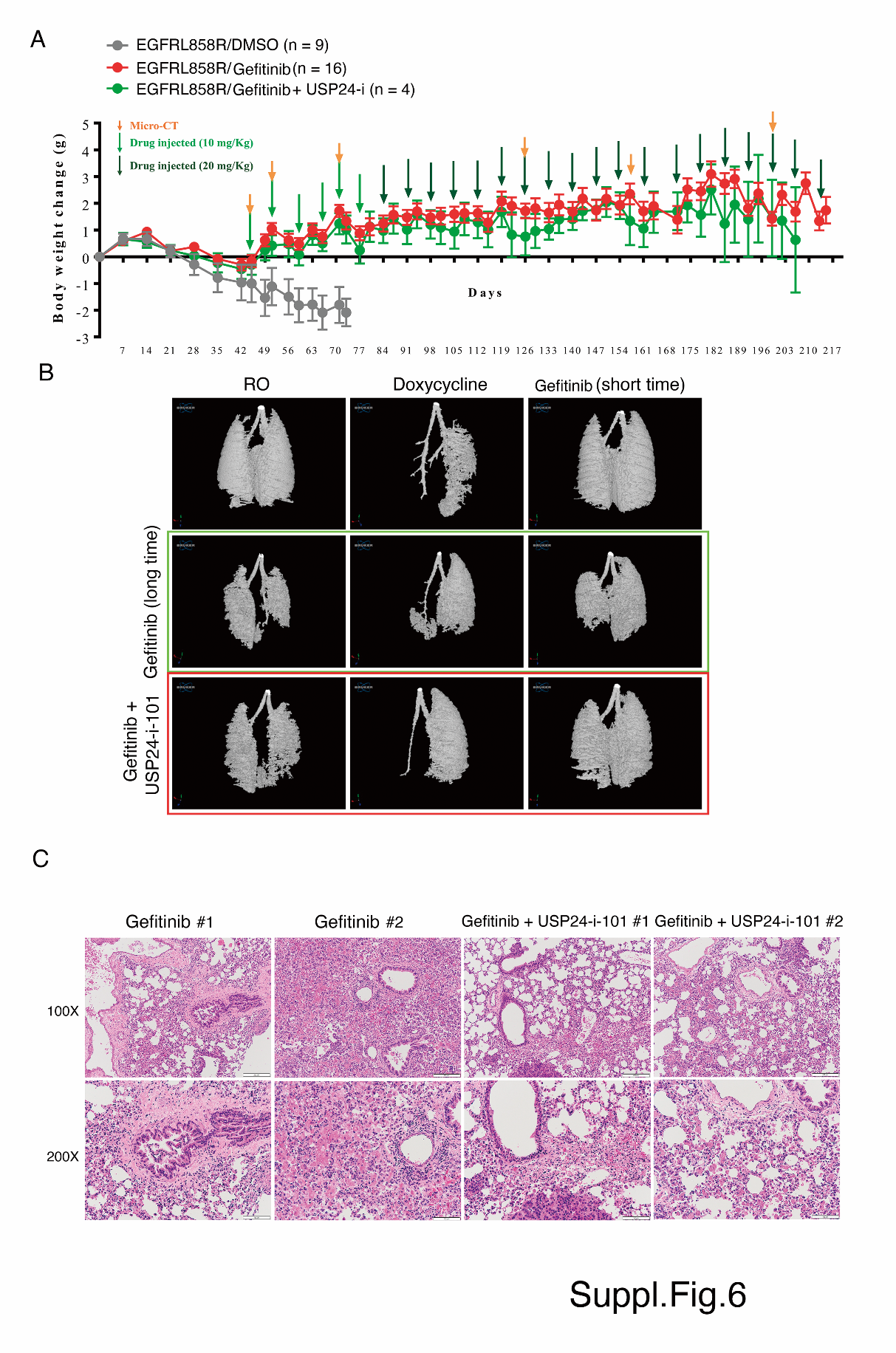


**Suppl. Fig. 6.** Targeting USP24 in *EGFR^L858R^* lung cancer drug resistant mice. (A). *EGFR^L858R^*USP24^WT^* mice were treated with 10 mg/l doxycycline in the drinking water for 6 weeks, and then treated with gefitinib (20 mg/kg) and USP24-i-101 (10 mg/kg) for 217 days. Tumor growth *in vivo* was studied by body weight every week (A) and micro-CT (B). After sacrifice, the pathology in the lung organs was studied by H&E staining, and the tumor area in the lung tissues was quantitated (C).
