# Supplementary material for: USP24-i-101 targeting of USP24 activates autophagy to inhibit drug resistance acquired during cancer therapy
Source: Cell Death Differ. 2024 Mar 15;31(5):574–91. doi: 10.1038/s41418-024-01277-7 (PMC11093971; doi:10.1038/s41418-024-01277-7)
Supplement: Supplementary file 3 — Supplementary- All Raw Western Files [file 41418_2024_1277_MOESM3_ESM.pdf]

Raw data of Western blot

Fig1.A(a)

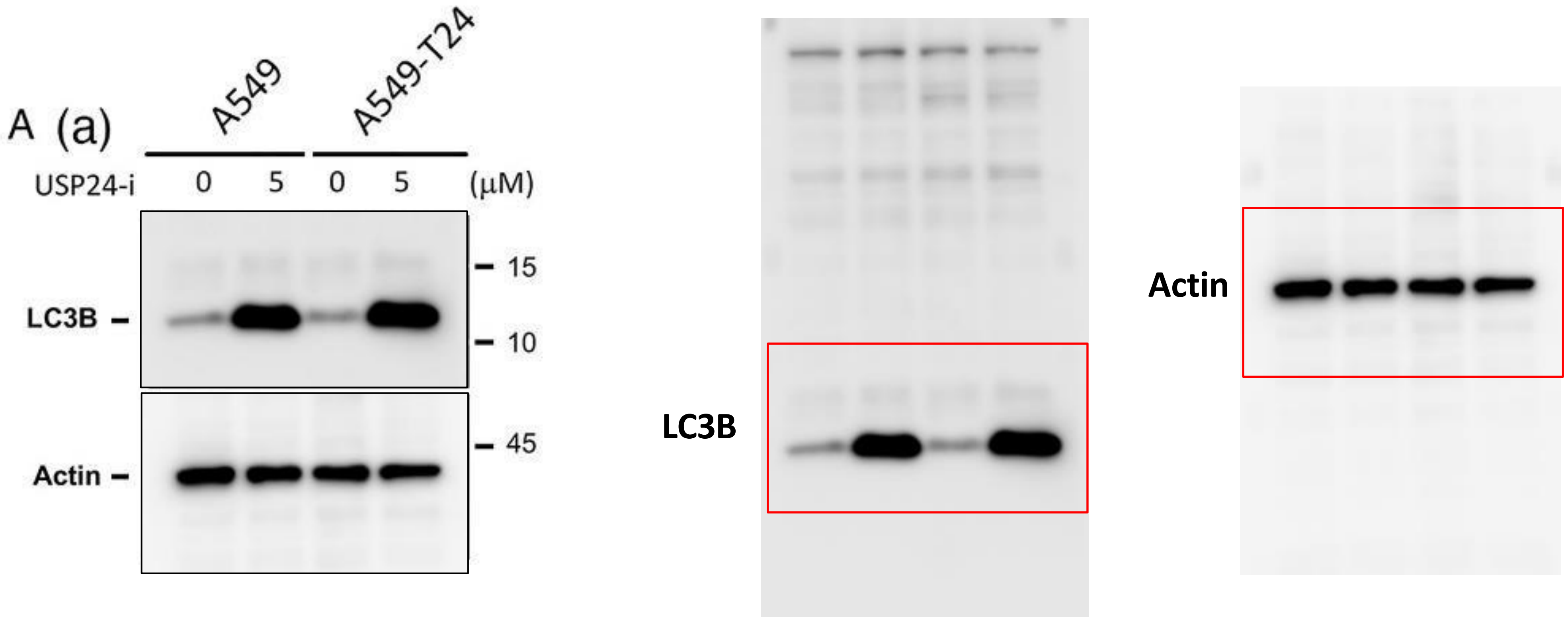

Fig1.A(b)

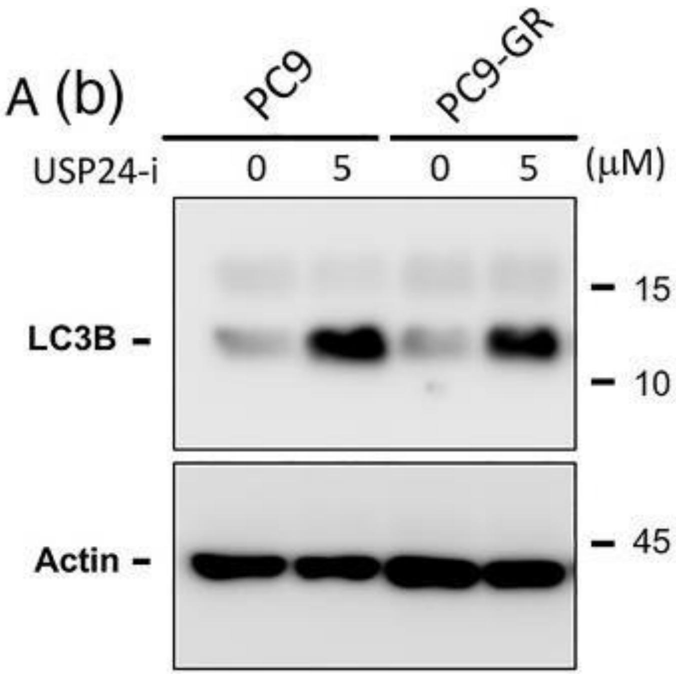

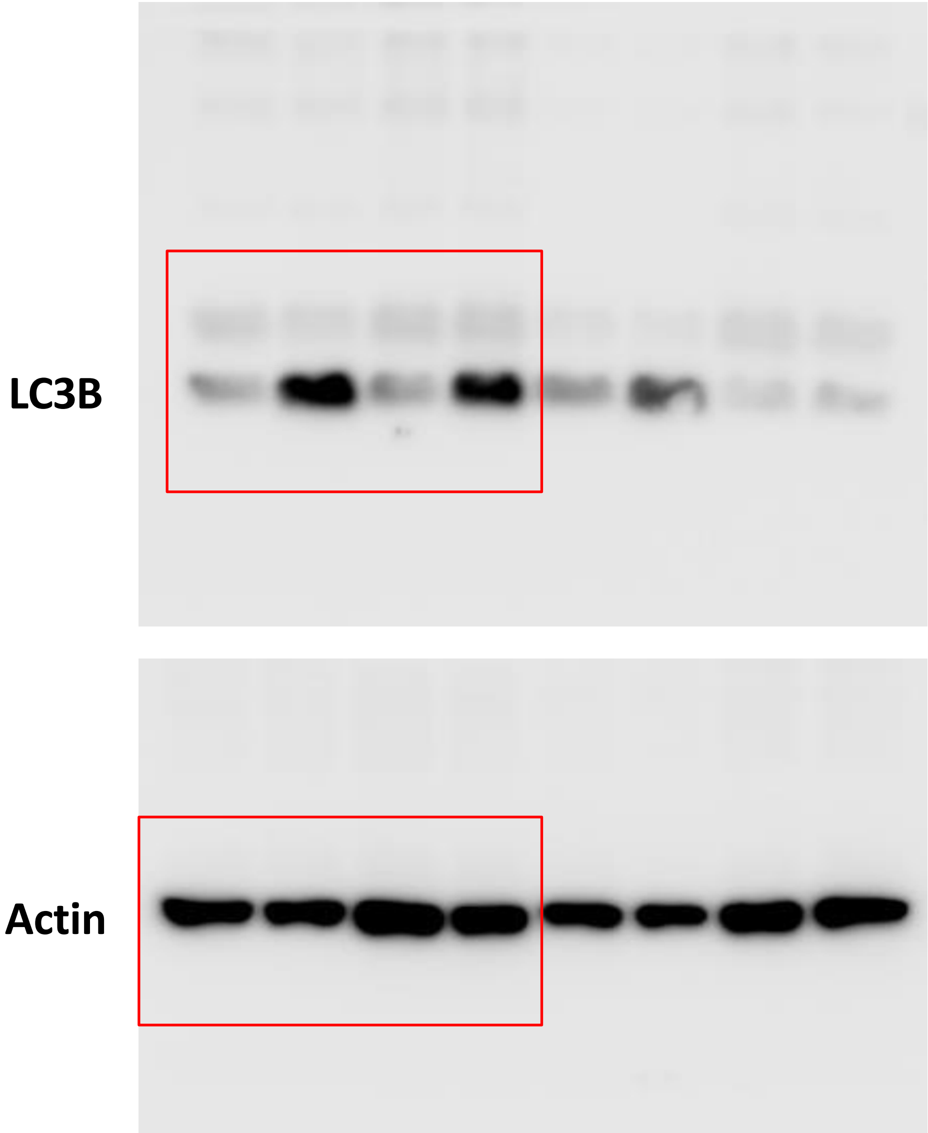

Fig1.A(c)

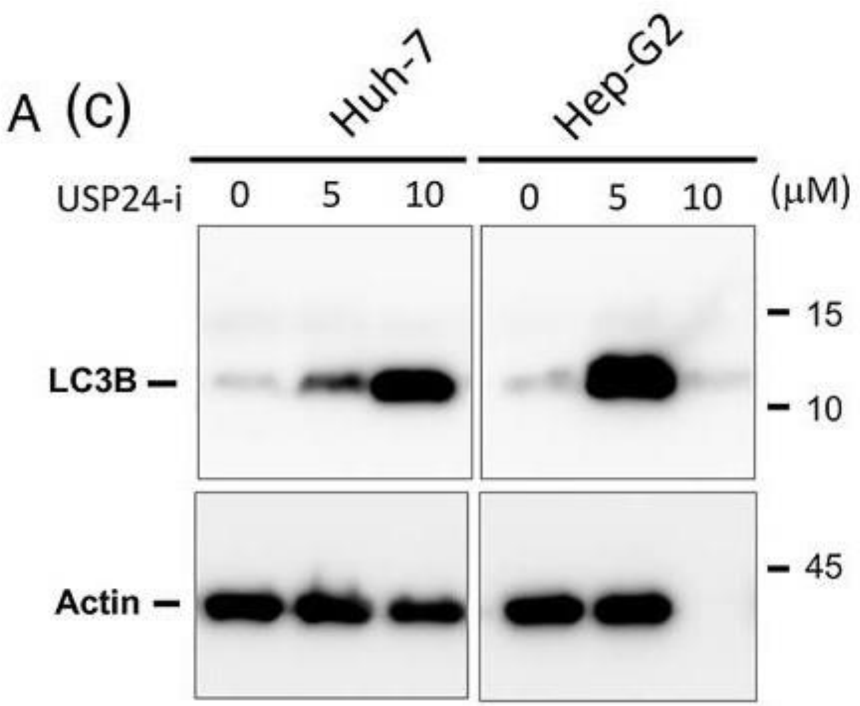
A (c)
Huh-7
Hep-G2
USP24-i 0 5 10 0 5 10 (μM)
LC3B
Actin
15
10
45

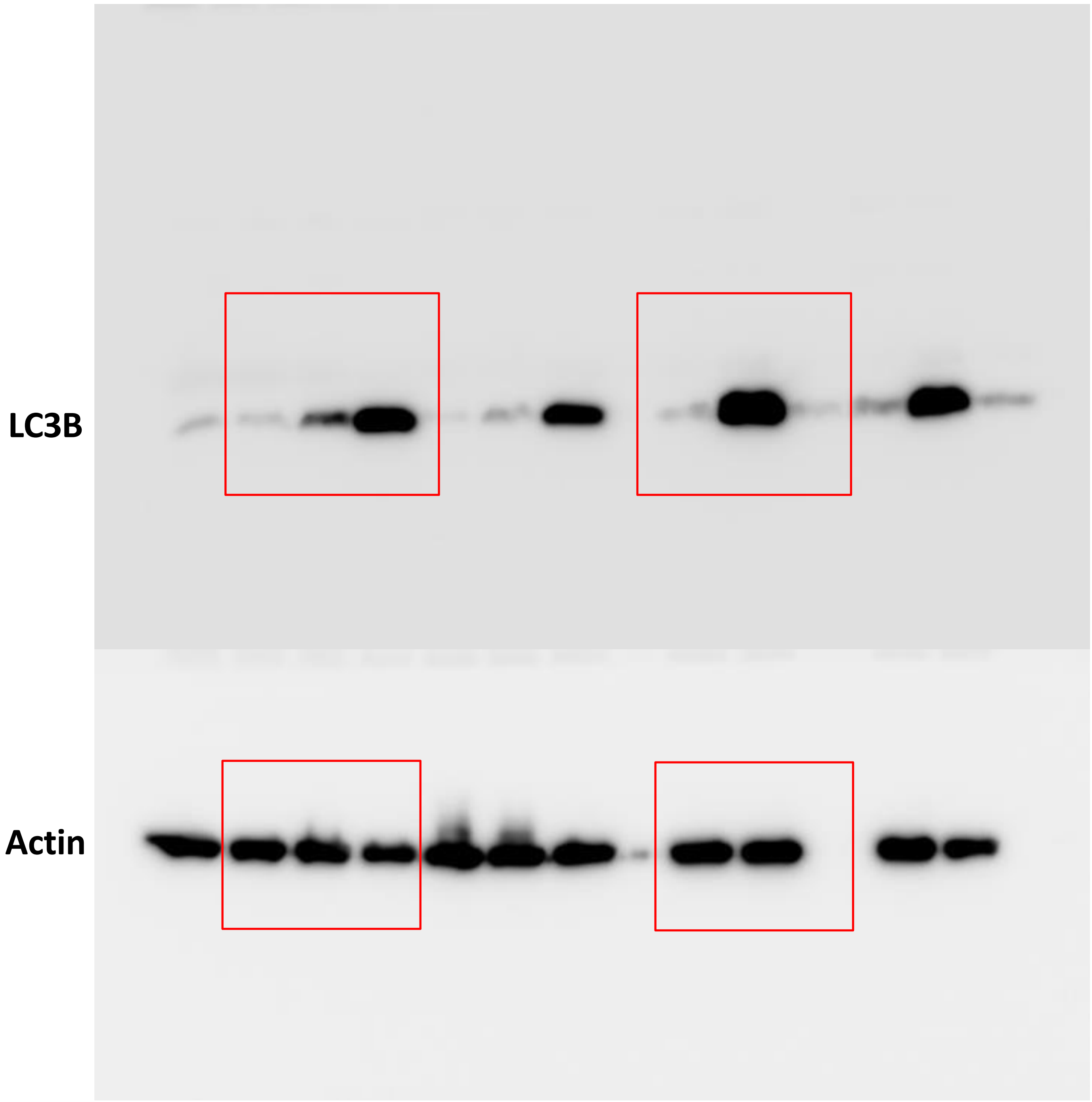
LC3B
Actin

Fig1.A(d)

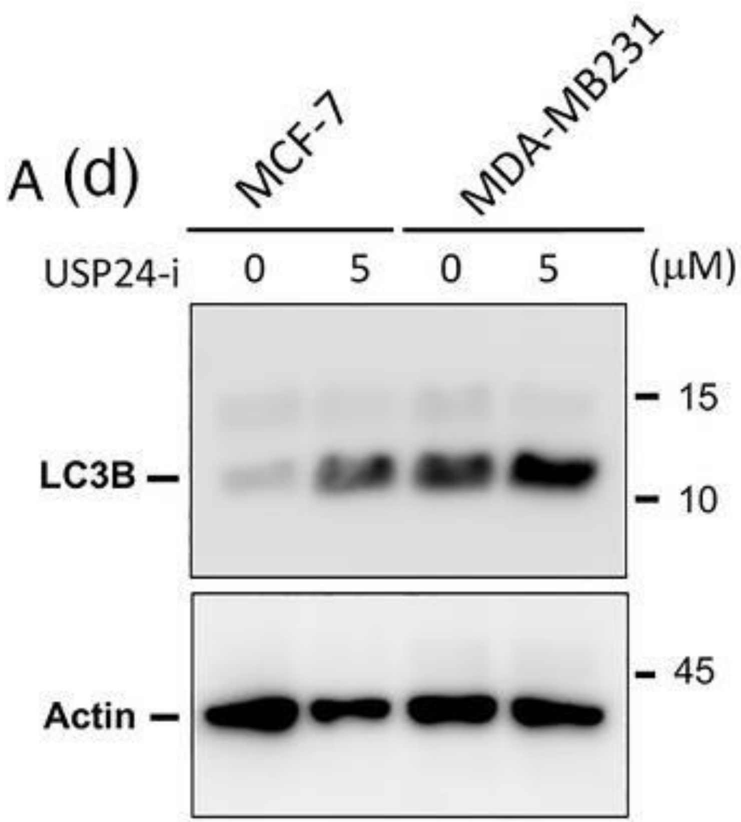
A (d)
MCF-7
MDA-MB231
USP24-i
0
5
0
5
(μM)
LC3B
Actin
15
10
45

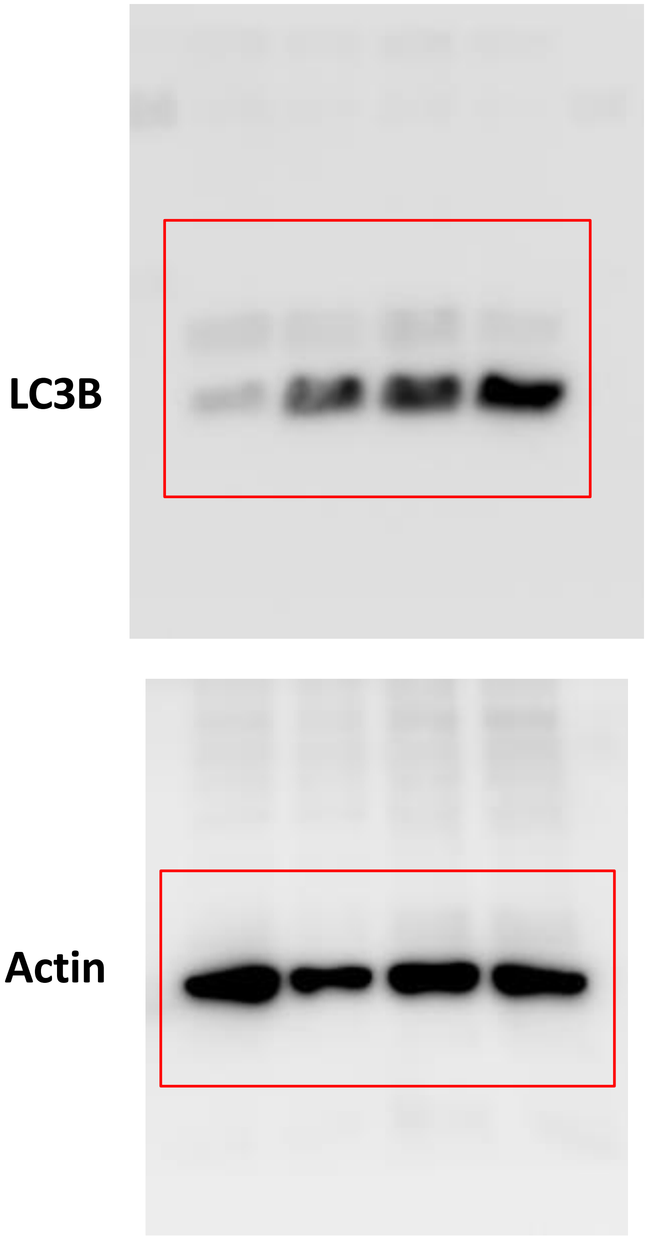
LC3B
Actin

Fig1.B(a)

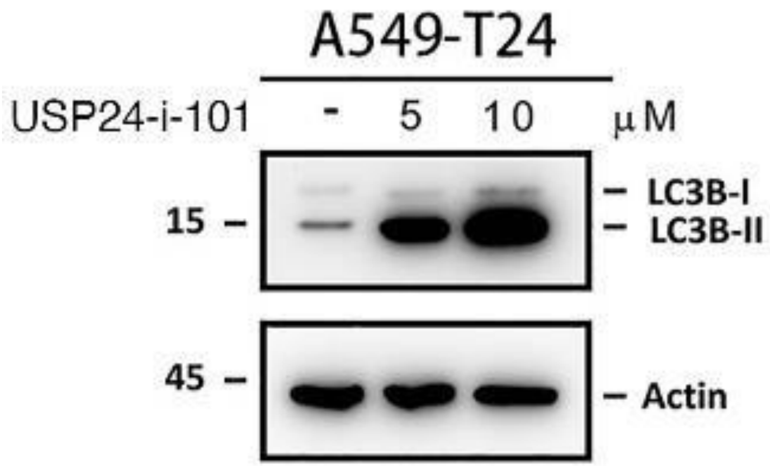
A549-T24
USP24-i-101
-
5
10
μM
15
45
LC3B-I
LC3B-II
Actin

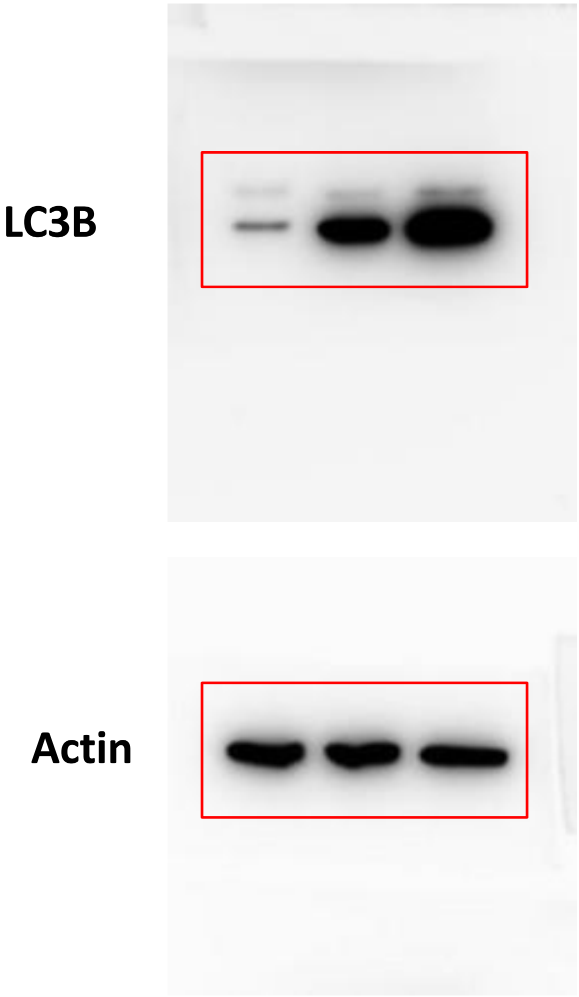
LC3B
Actin

Fig1.C(a)

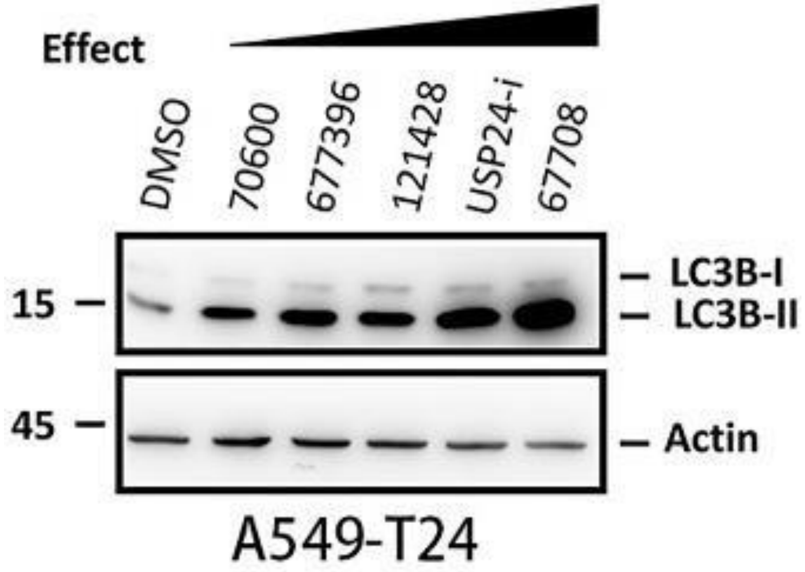
Effect
DMSO
70600
677396
121428
USP24-i
67708
15
45
LC3B-I
LC3B-II
Actin
A549-T24

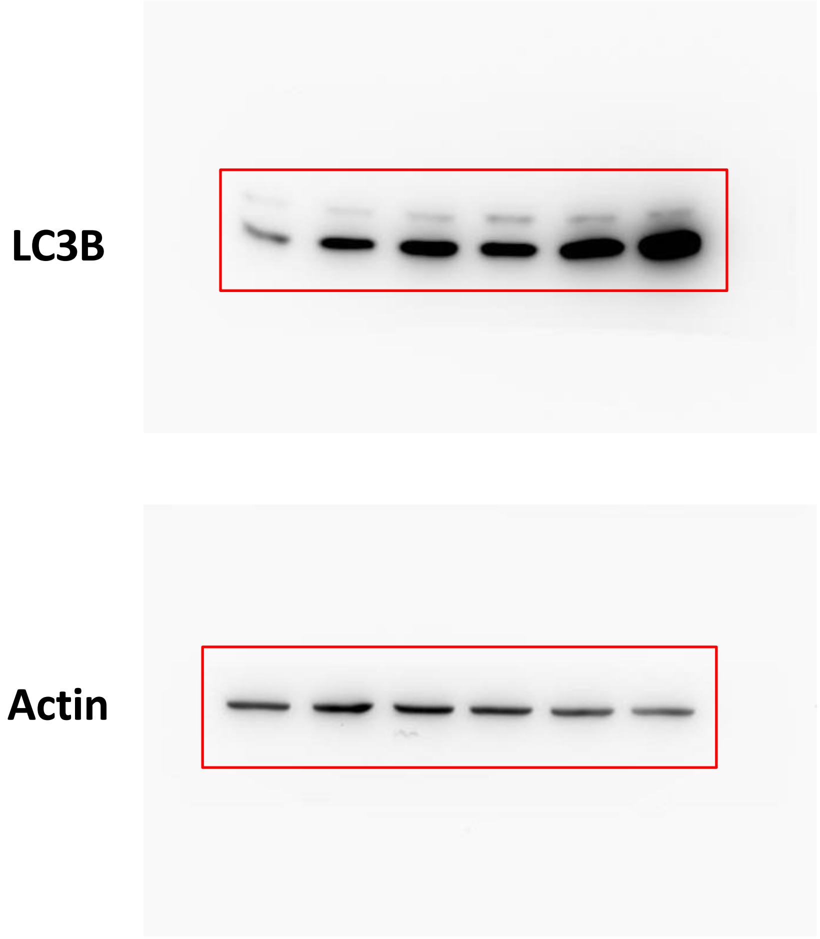
LC3B
Actin

Fig1.G(a)

❶

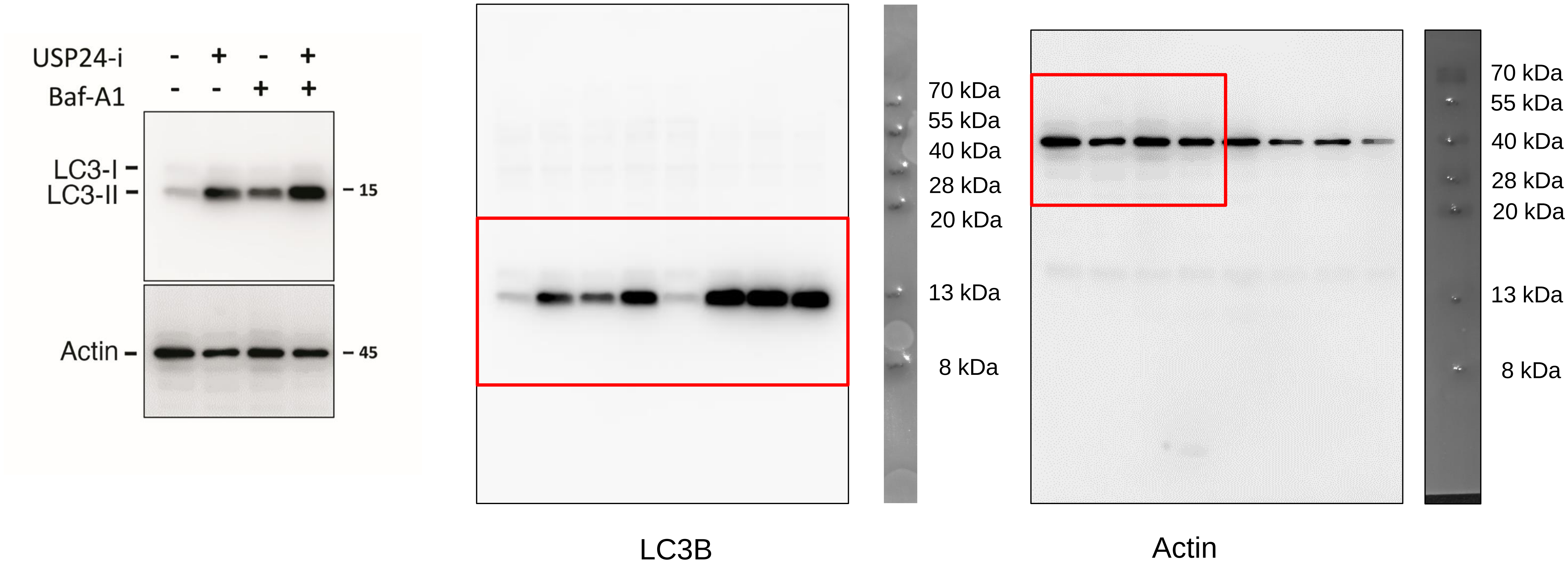
USP24-i - + - +
Baf-A1 - - + +
LC3-I
LC3-II
- 15
Actin
- 45
70 kDa
55 kDa
40 kDa
28 kDa
20 kDa
13 kDa
8 kDa
70 kDa
55 kDa
40 kDa
28 kDa
20 kDa
13 kDa
8 kDa
LC3B
Actin

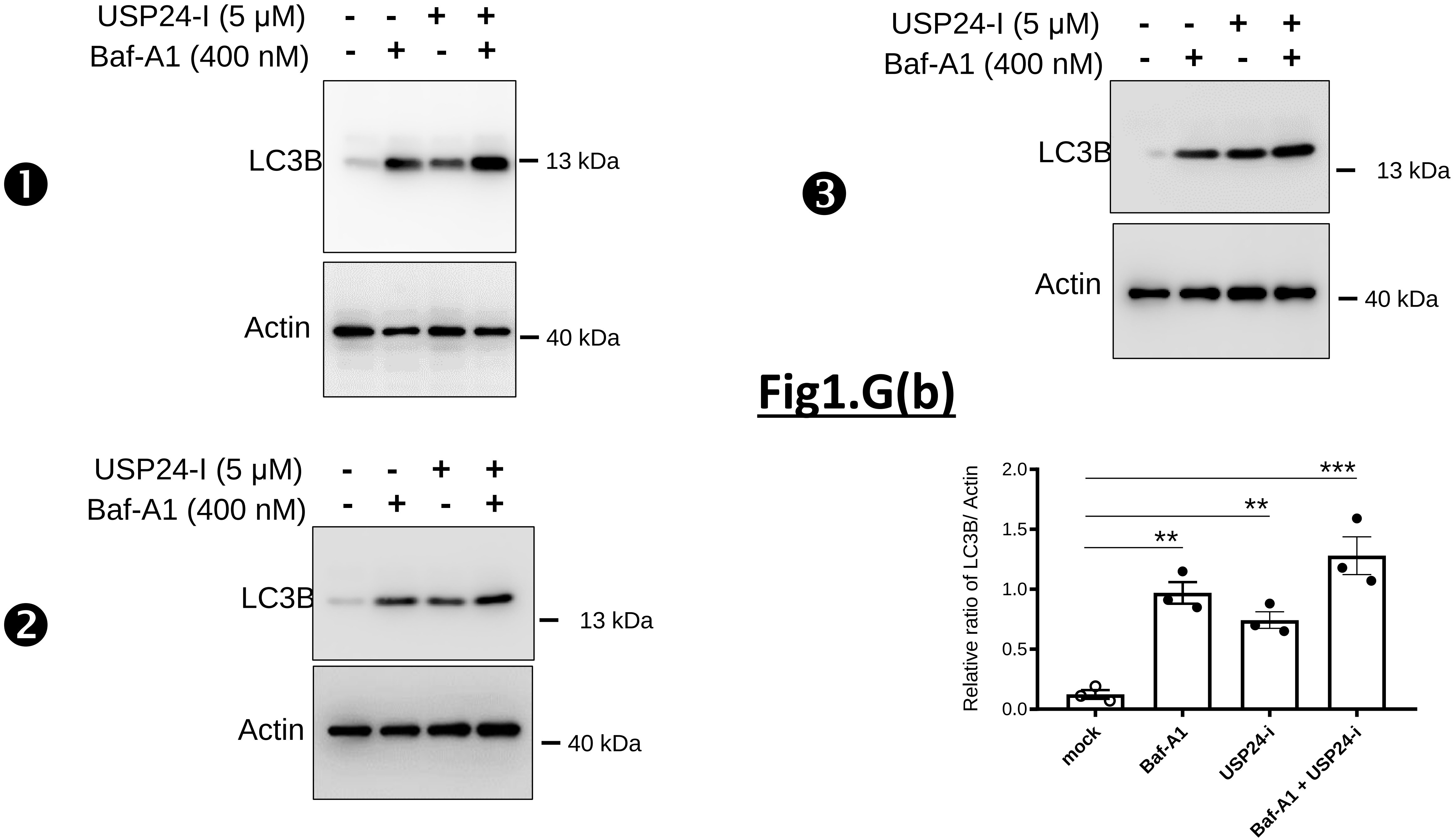

❶
USP24-I (5 μM) - - + +
Baf-A1 (400 nM) - + - +
LC3B
13 kDa
Actin
40 kDa
❷
USP24-I (5 μM) - - + +
Baf-A1 (400 nM) - + - +
LC3B
13 kDa
Actin
40 kDa
❸
USP24-I (5 μM) - - + +
Baf-A1 (400 nM) - + - +
LC3B
13 kDa
Actin
40 kDa
Relative ratio of LC3B/ Actin
2.0
1.5
1.0
0.5
0.0
***
**
**
mock
Baf-A1
USP24-i
Baf-A1 + USP24-i

Fig1.G(b)

❷

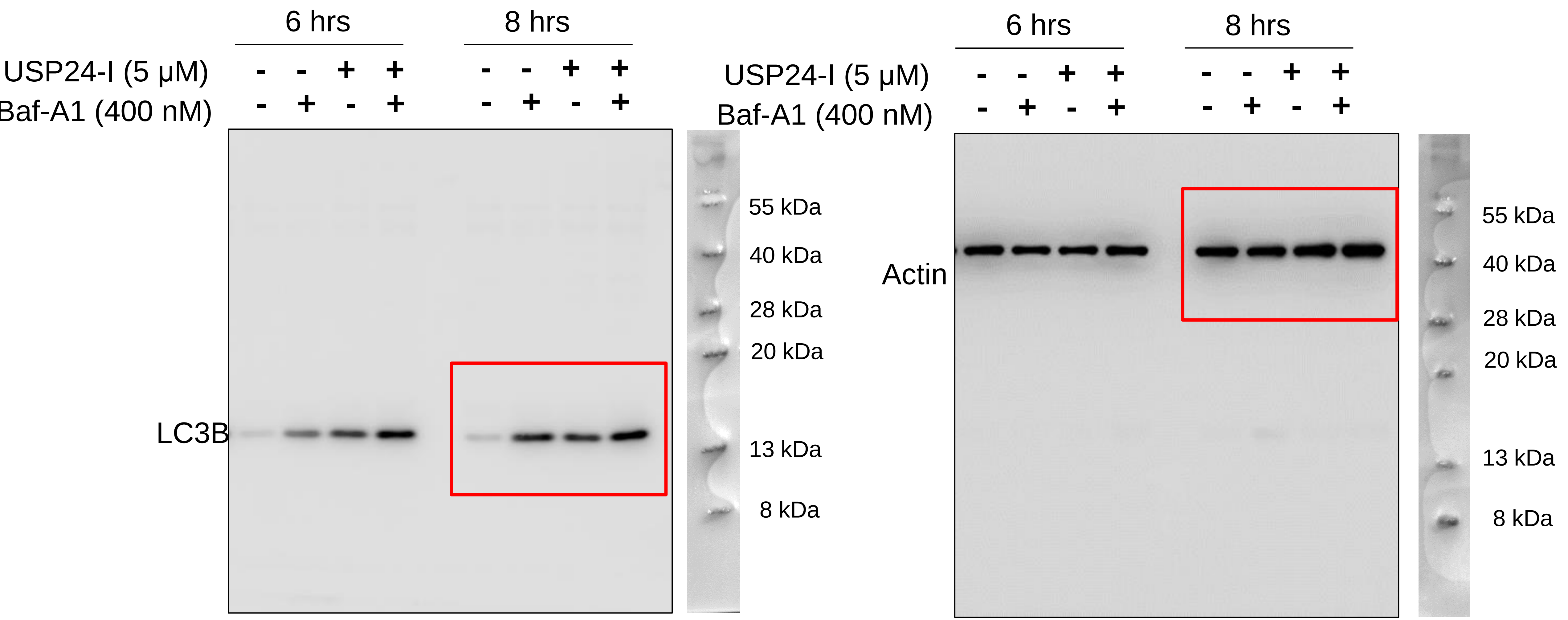
6 hrs
8 hrs
USP24-I (5 μM) - - + + - - + +
Baf-A1 (400 nM) - + - + - + - +
LC3B
55 kDa
40 kDa
28 kDa
20 kDa
13 kDa
8 kDa
6 hrs
8 hrs
USP24-I (5 μM) - - + + - - + +
Baf-A1 (400 nM) - + - + - + - +
Actin
55 kDa
40 kDa
28 kDa
20 kDa
13 kDa
8 kDa

❸

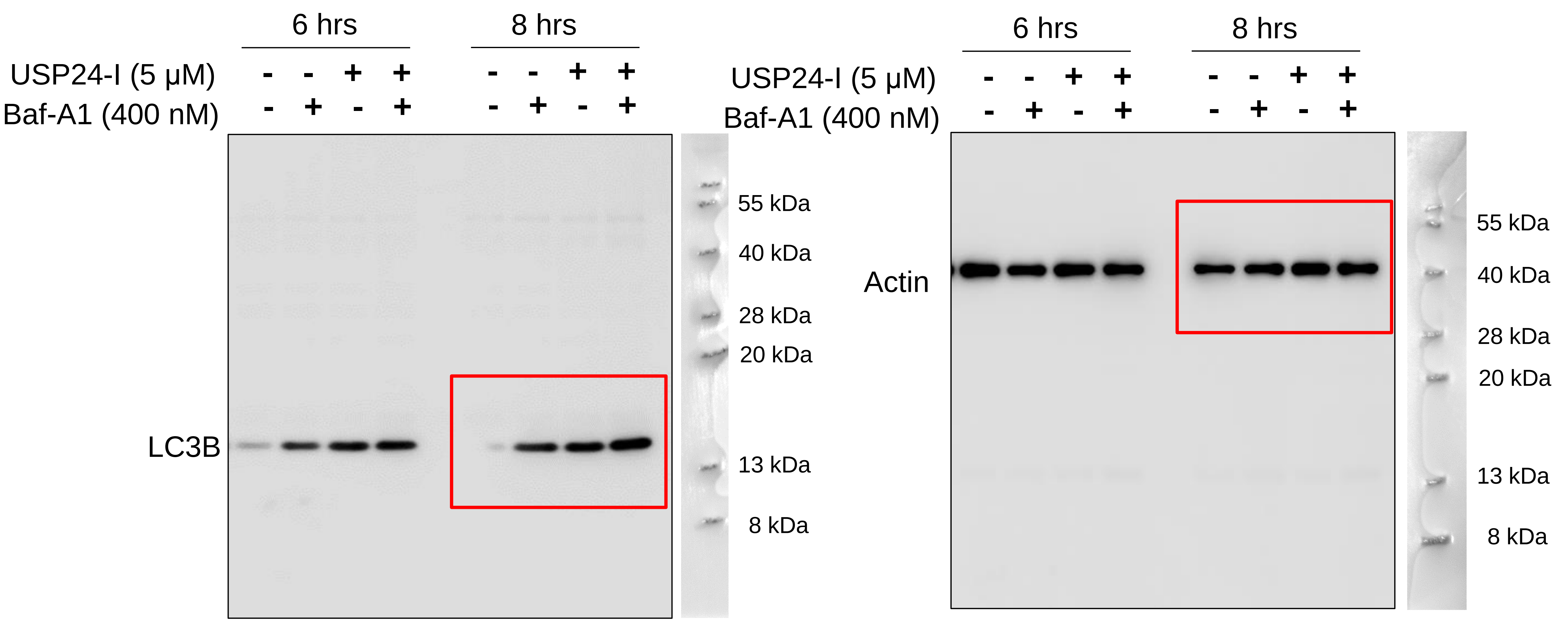
6 hrs
8 hrs
USP24-I (5 μM) - - + + - - + +
Baf-A1 (400 nM) - + - + - + - +
LC3B
55 kDa
40 kDa
28 kDa
20 kDa
13 kDa
8 kDa
6 hrs
8 hrs
USP24-I (5 μM) - - + + - - + +
Baf-A1 (400 nM) - + - + - + - +
Actin
55 kDa
40 kDa
28 kDa
20 kDa
13 kDa
8 kDa

Fig1.H

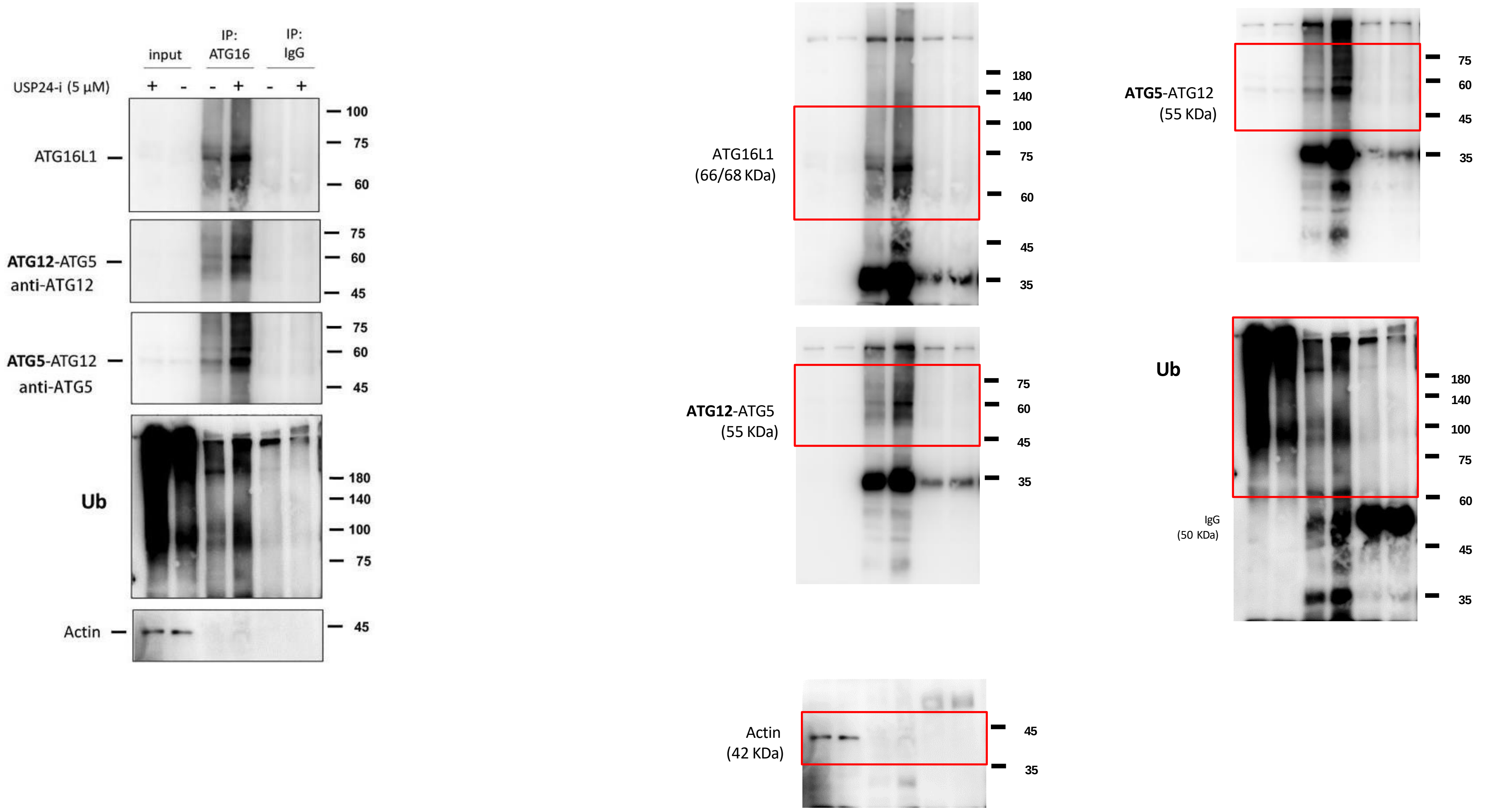
input
IP: ATG16
IP: IgG
USP24-i (5 μM)
+ - - + - +
ATG16L1
ATG12-ATG5
anti-ATG12
ATG5-ATG12
anti-ATG5
Ub
Actin
100
75
60
45
180
140
35
ATG16L1
(66/68 KDa)
ATG12-ATG5
(55 KDa)
Actin
(42 KDa)
ATG5-ATG12
(55 KDa)
Ub
IgG
(50 KDa)

Fig1.I(a)

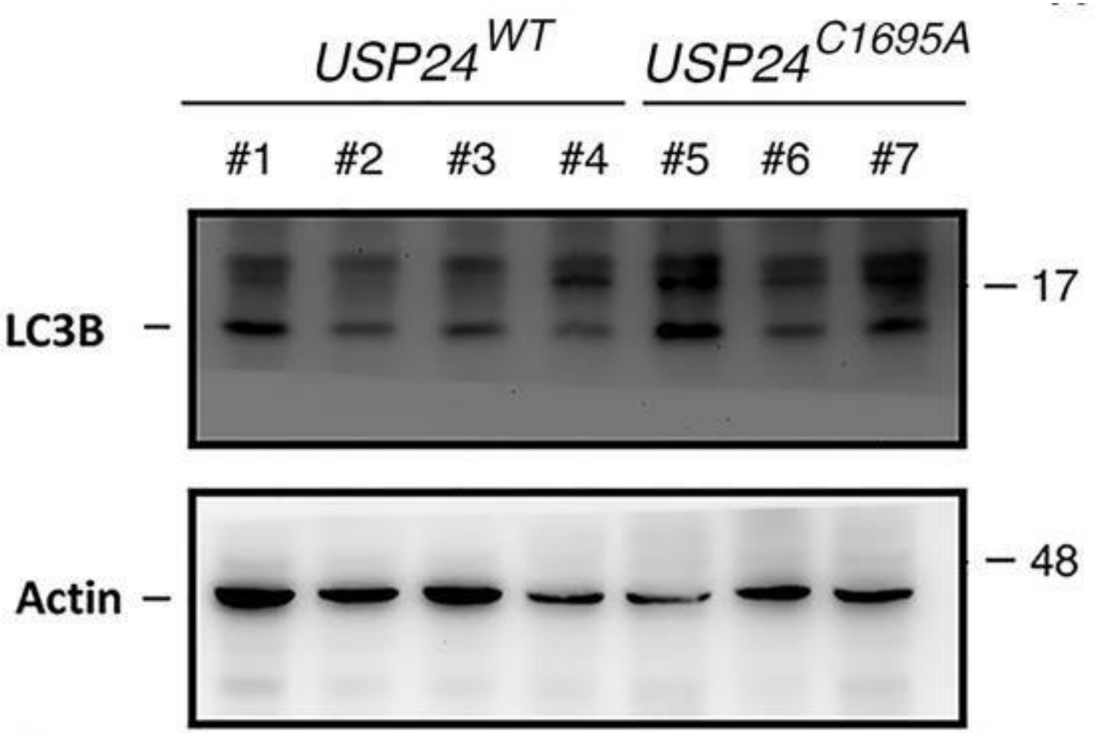

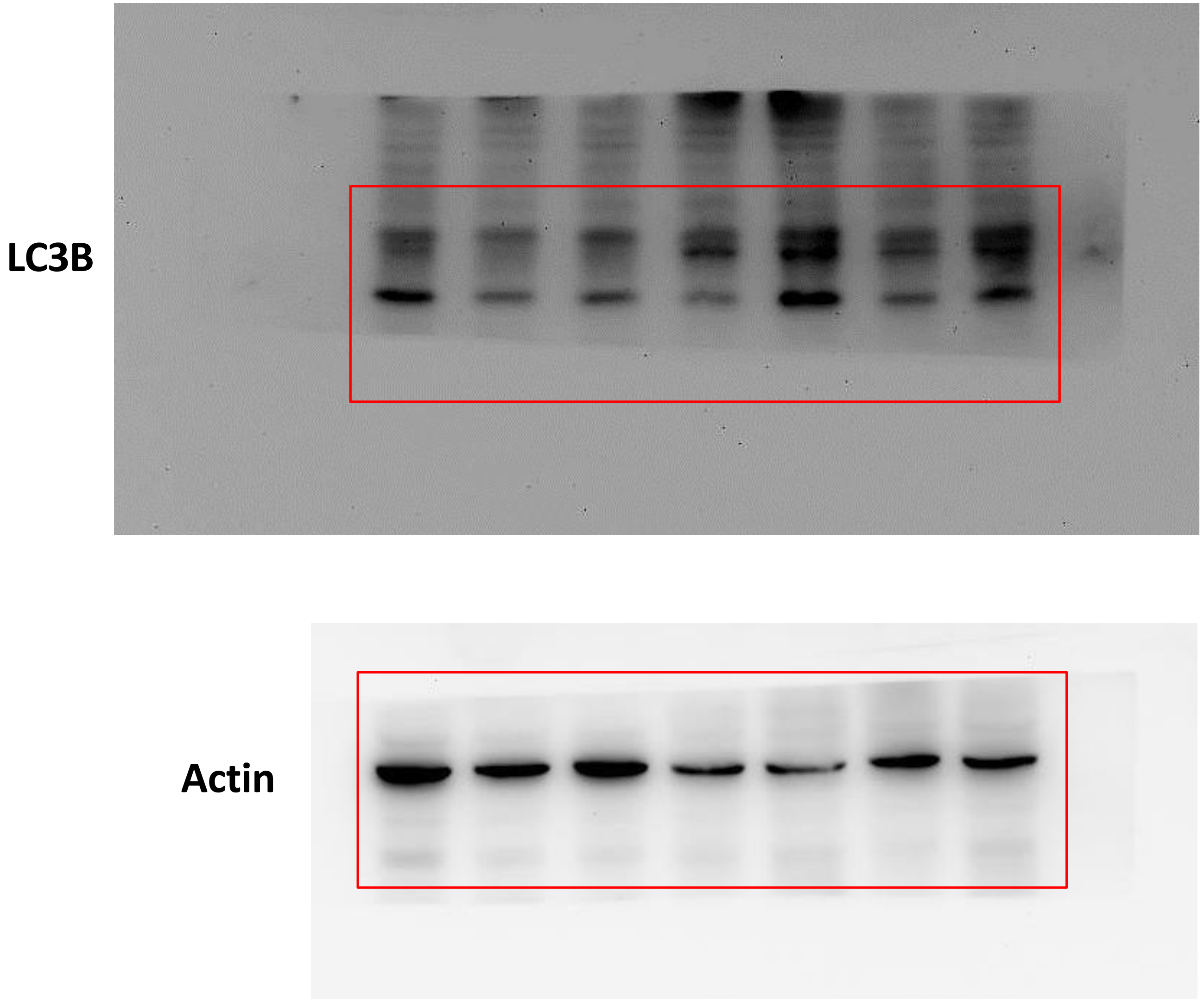

Fig1.J(a)

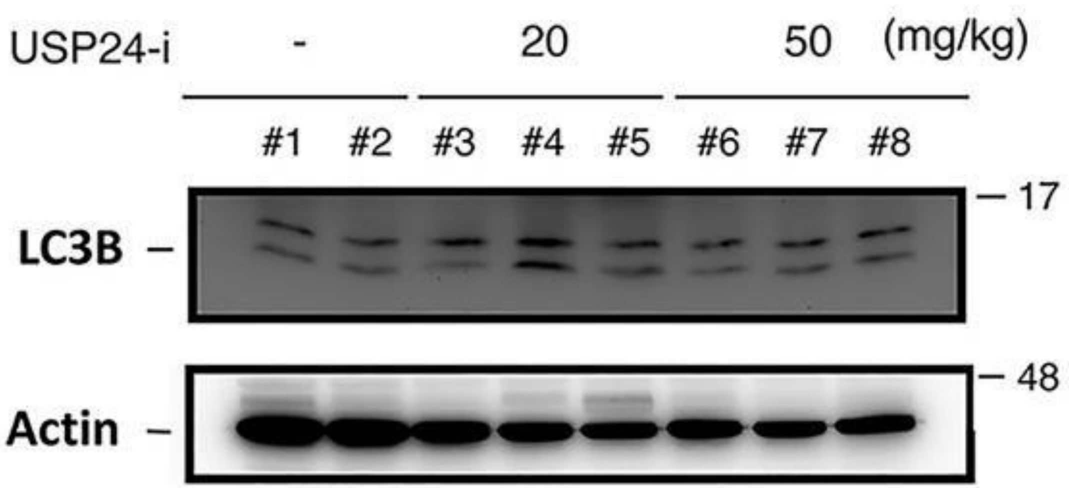

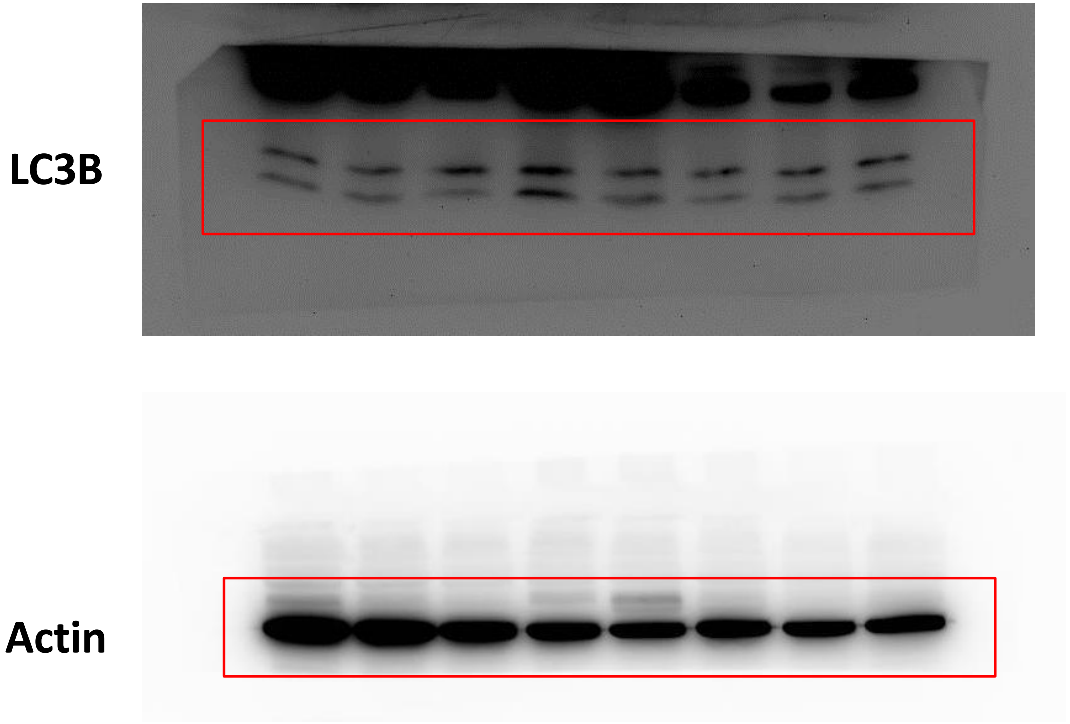

Fig2.A(a)

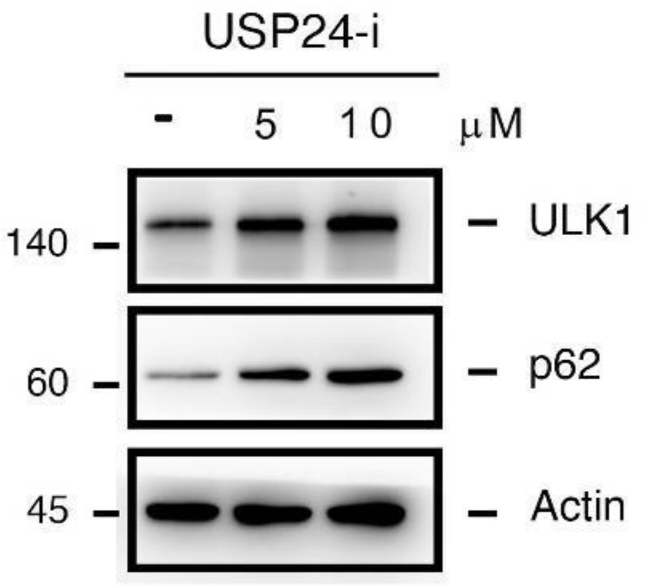

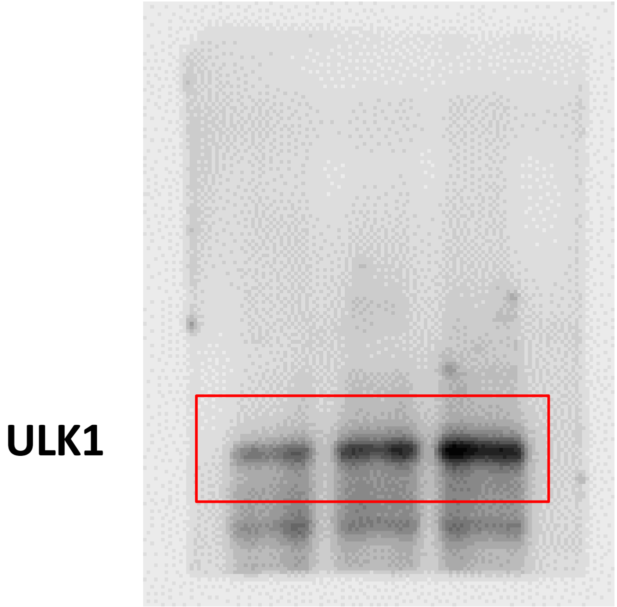

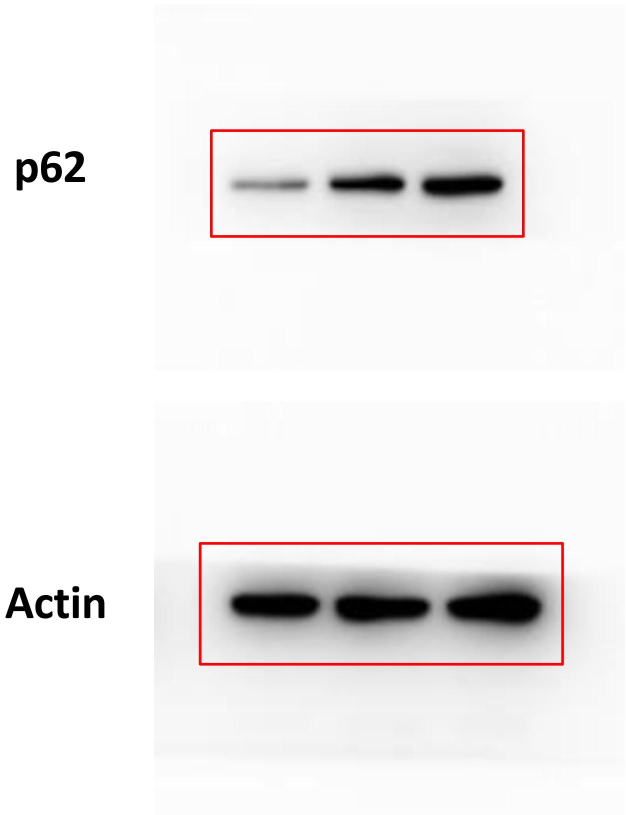

Fig2.C(a)

A549-T24

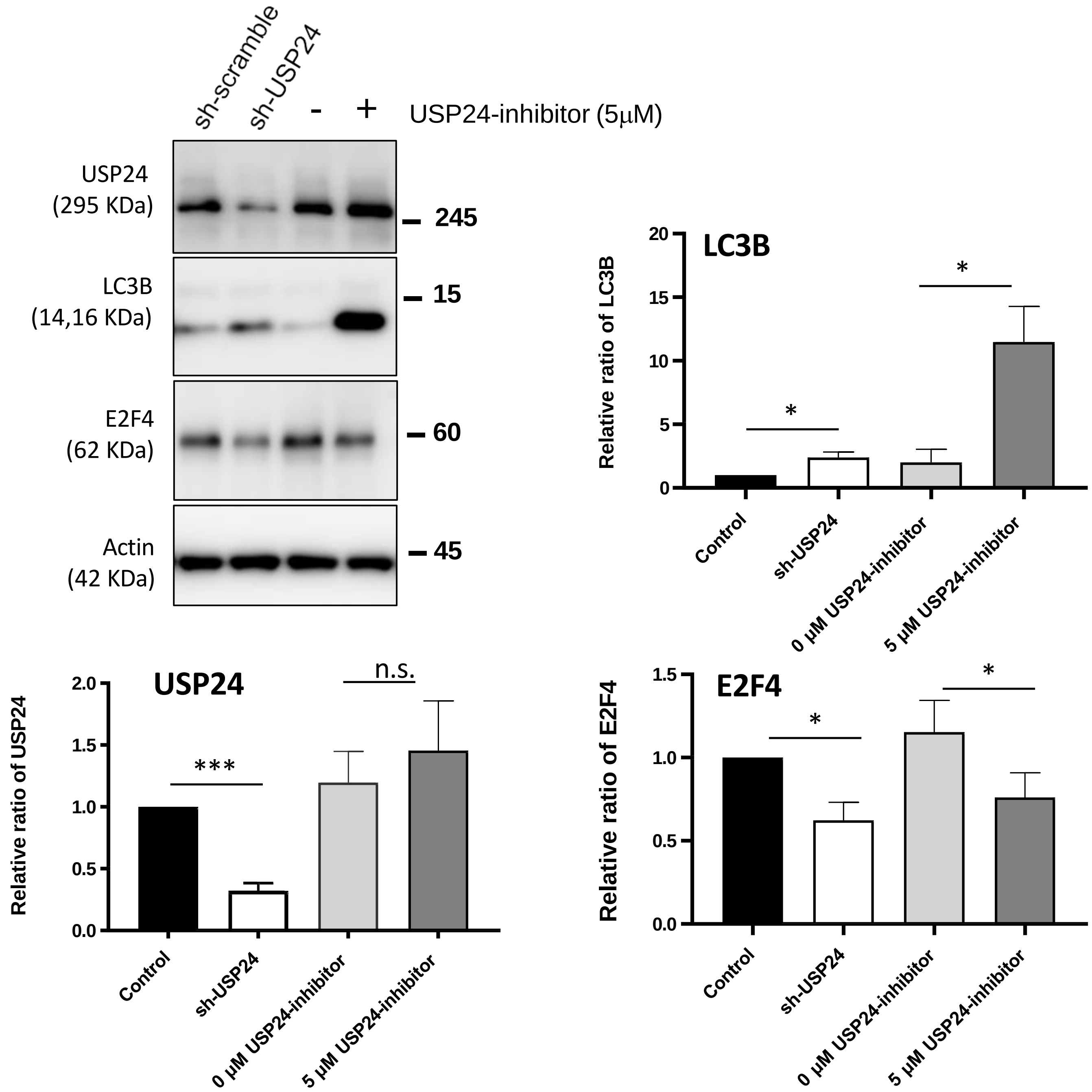

Fig2.C(a)

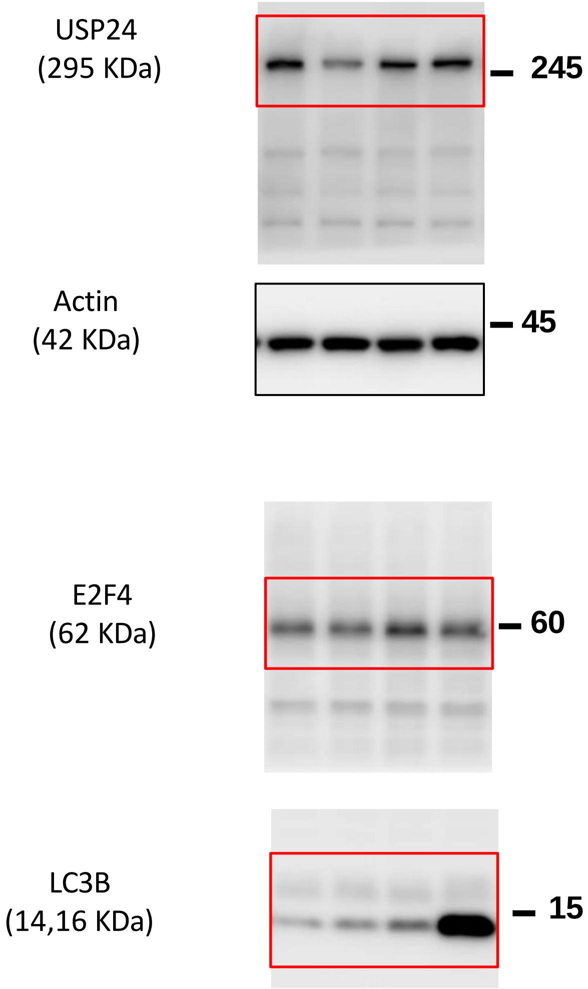
USP24
(295 KDa)
245
Actin
(42 KDa)
45
E2F4
(62 KDa)
60
LC3B
(14,16 KDa)
15

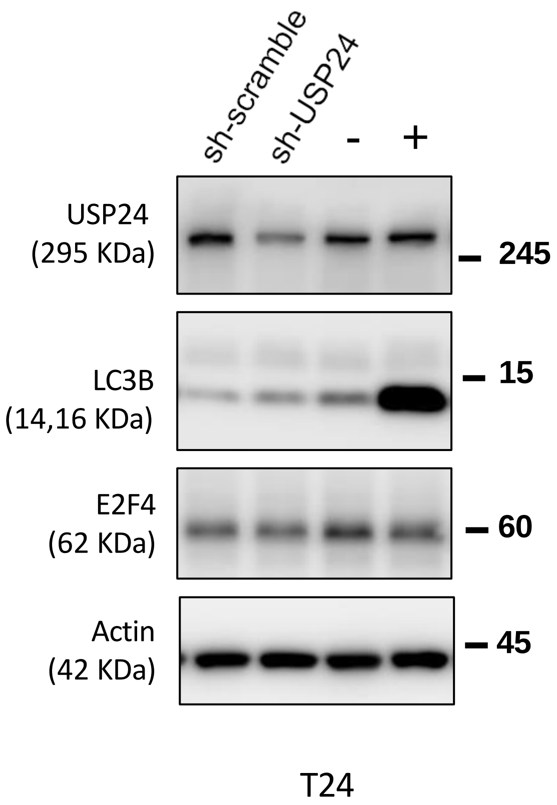
sh-scramble
sh-USP24
-
+
USP24
(295 KDa)
245
LC3B
(14,16 KDa)
15
E2F4
(62 KDa)
60
Actin
(42 KDa)
45
T24

1

Fig2.C(a)

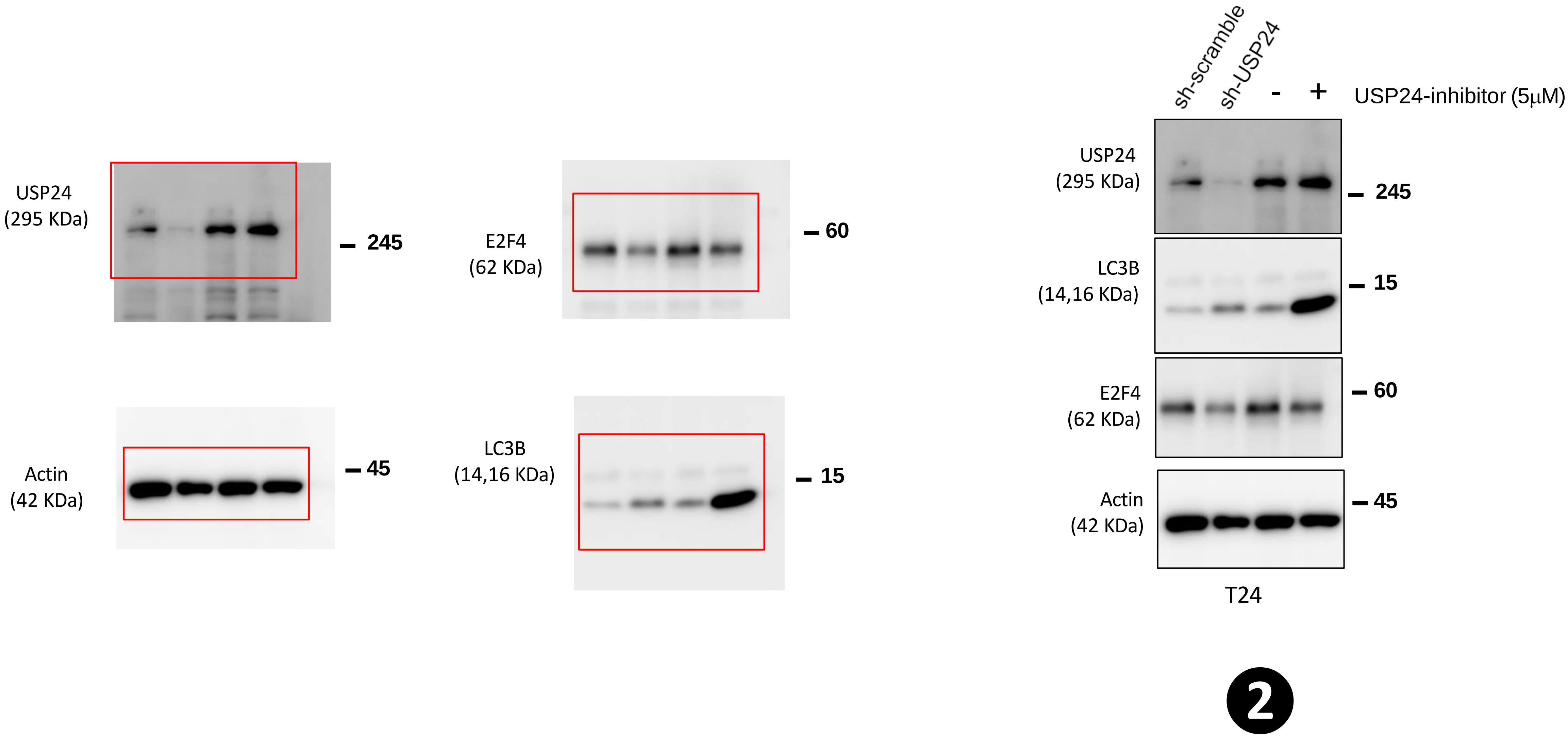
USP24
(295 KDa)
245
E2F4
(62 KDa)
60
Actin
(42 KDa)
45
LC3B
(14,16 KDa)
15
sh-scramble
sh-USP24
-
+
USP24-inhibitor (5µM)
USP24
(295 KDa)
245
LC3B
(14,16 KDa)
15
E2F4
(62 KDa)
60
Actin
(42 KDa)
45
T24
2

Fig2.C(a)

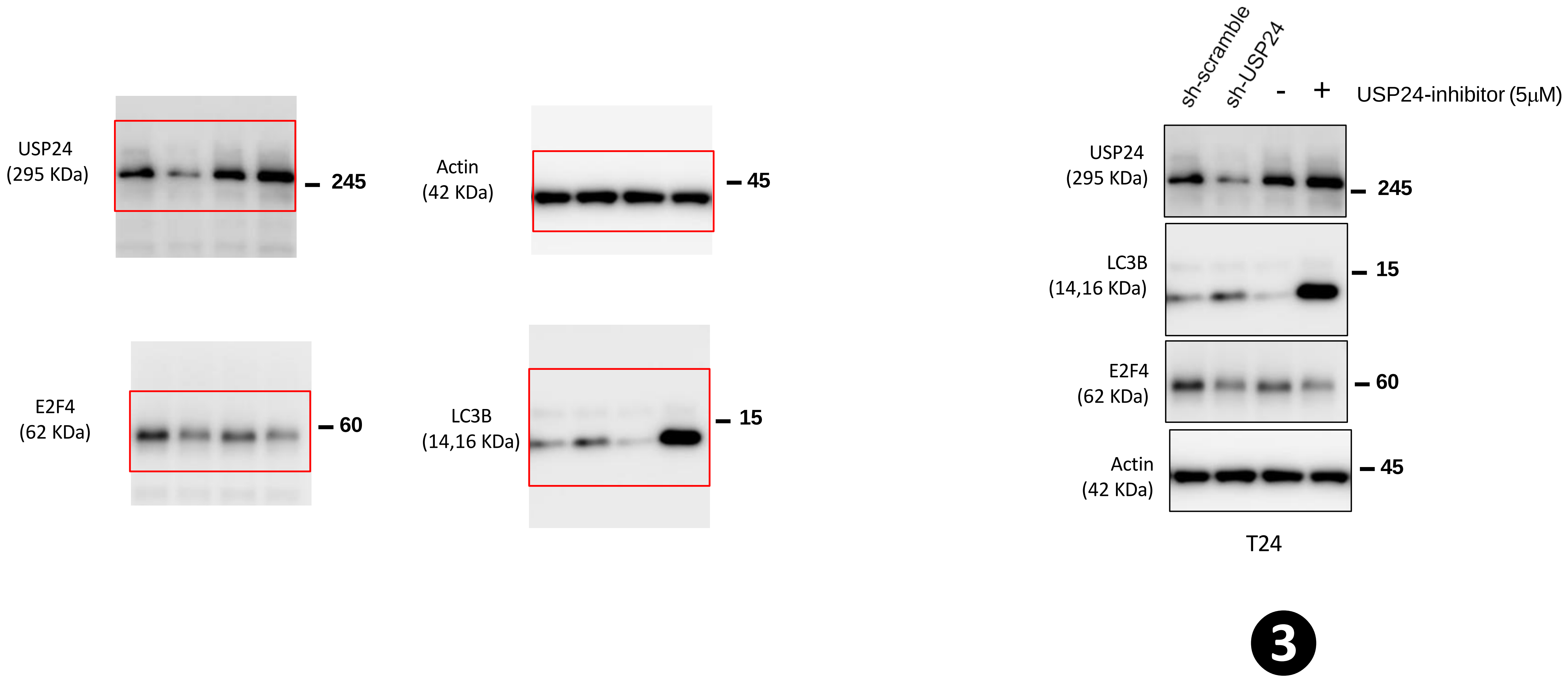
USP24
(295 KDa)
245
Actin
(42 KDa)
45
E2F4
(62 KDa)
60
LC3B
(14,16 KDa)
15
sh-scramble
sh-USP24
-
+
USP24-inhibitor (5μM)
USP24
(295 KDa)
245
LC3B
(14,16 KDa)
15
E2F4
(62 KDa)
60
Actin
(42 KDa)
45
T24
3

Fig2.D(a)

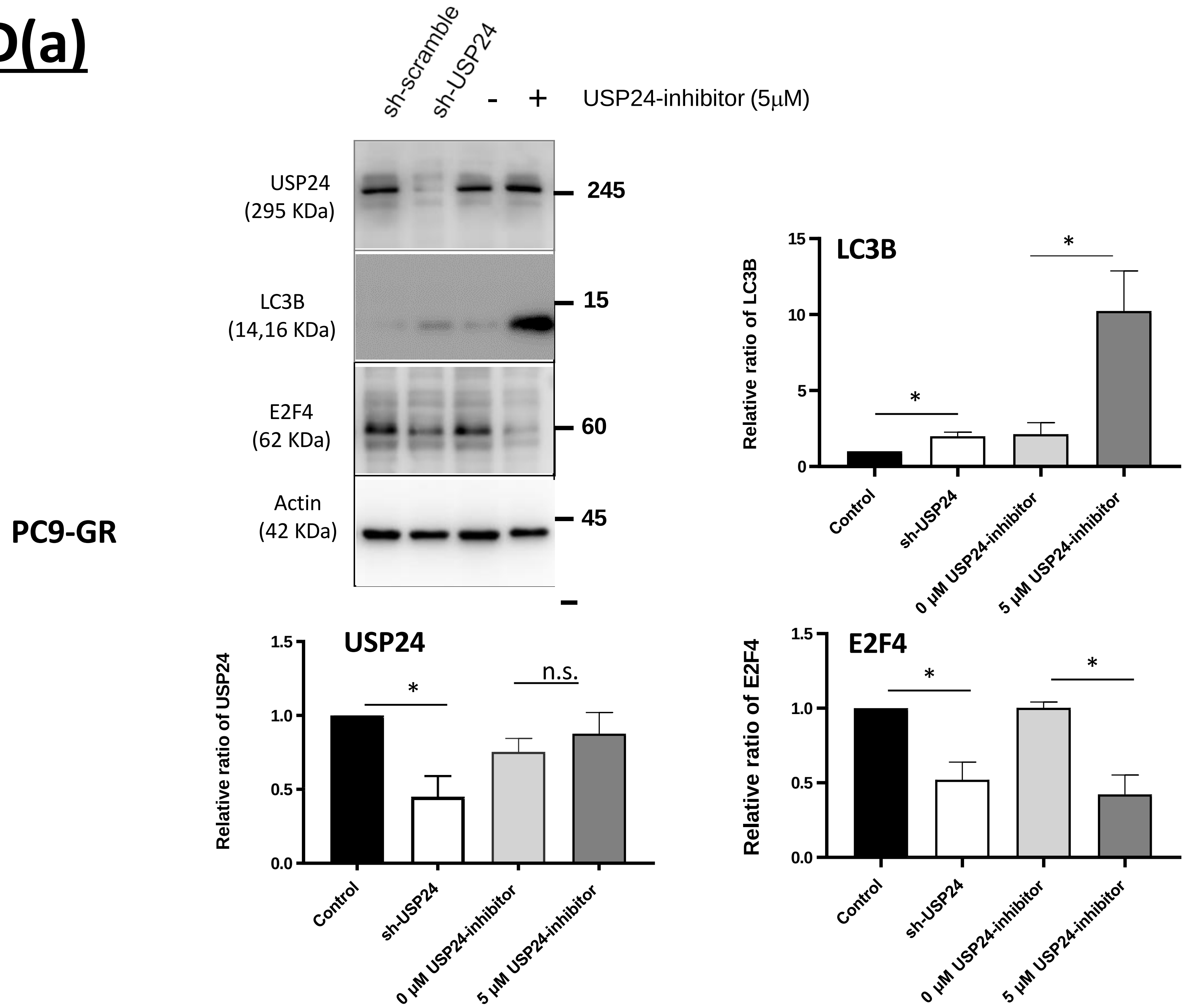
PC9-GR
sh-scramble
sh-USP24
- + USP24-inhibitor (5μM)
USP24
(295 KDa)
LC3B
(14,16 KDa)
E2F4
(62 KDa)
Actin
(42 KDa)
245
15
60
45
LC3B
Relative ratio of LC3B
15
10
5
0
*
*
Control
sh-USP24
0 μM USP24-inhibitor
5 μM USP24-inhibitor
USP24
Relative ratio of USP24
1.5
1.0
0.5
0.0
*
n.s.
Control
sh-USP24
0 μM USP24-inhibitor
5 μM USP24-inhibitor
E2F4
Relative ratio of E2F4
1.5
1.0
0.5
0.0
*
*
Control
sh-USP24
0 μM USP24-inhibitor
5 μM USP24-inhibitor

Fig2.D(a)

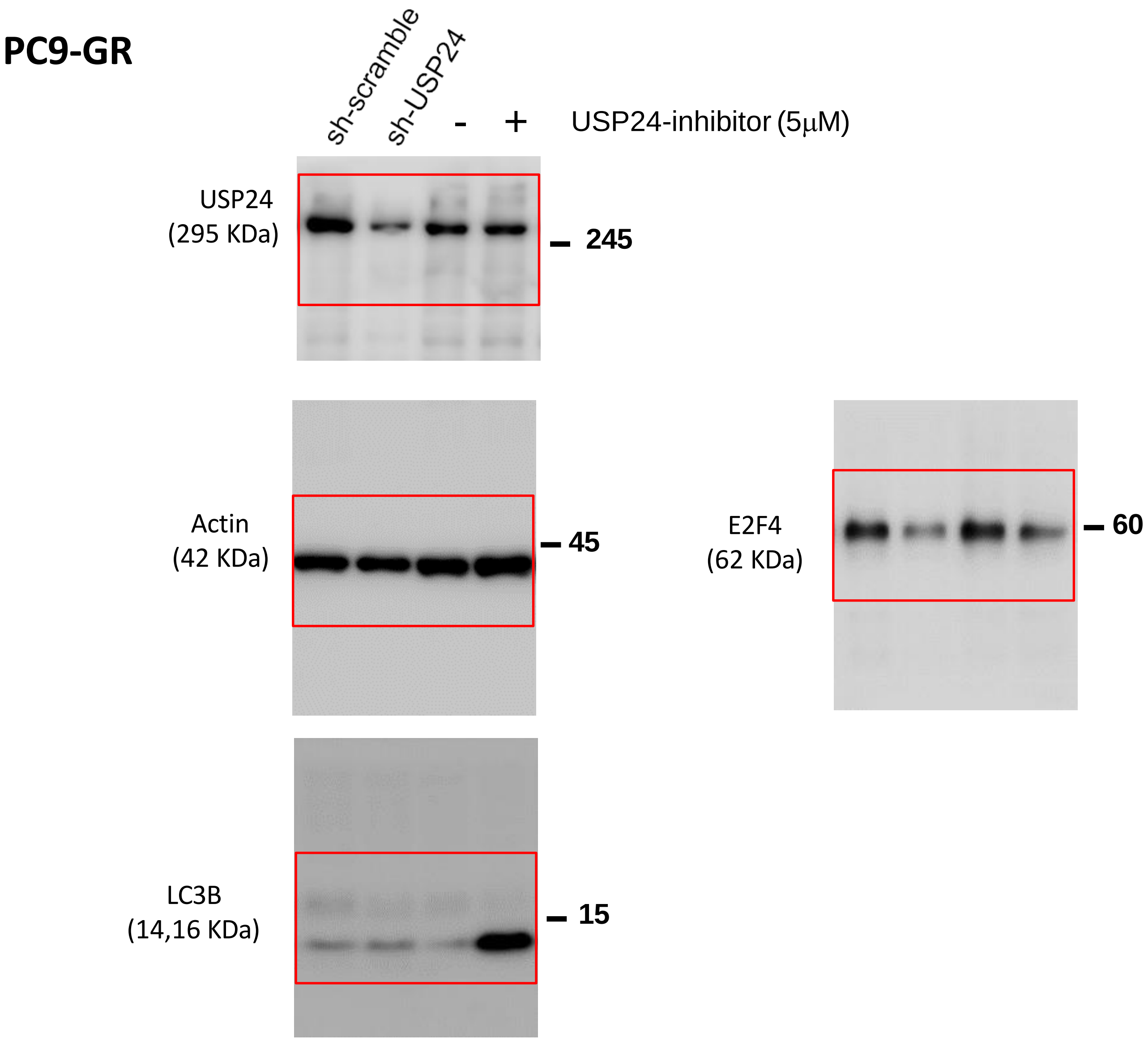
PC9-GR
sh-scramble
sh-USP24
-
+
USP24-inhibitor (5µM)
USP24
(295 KDa)
245
Actin
(42 KDa)
45
E2F4
(62 KDa)
60
LC3B
(14,16 KDa)
15

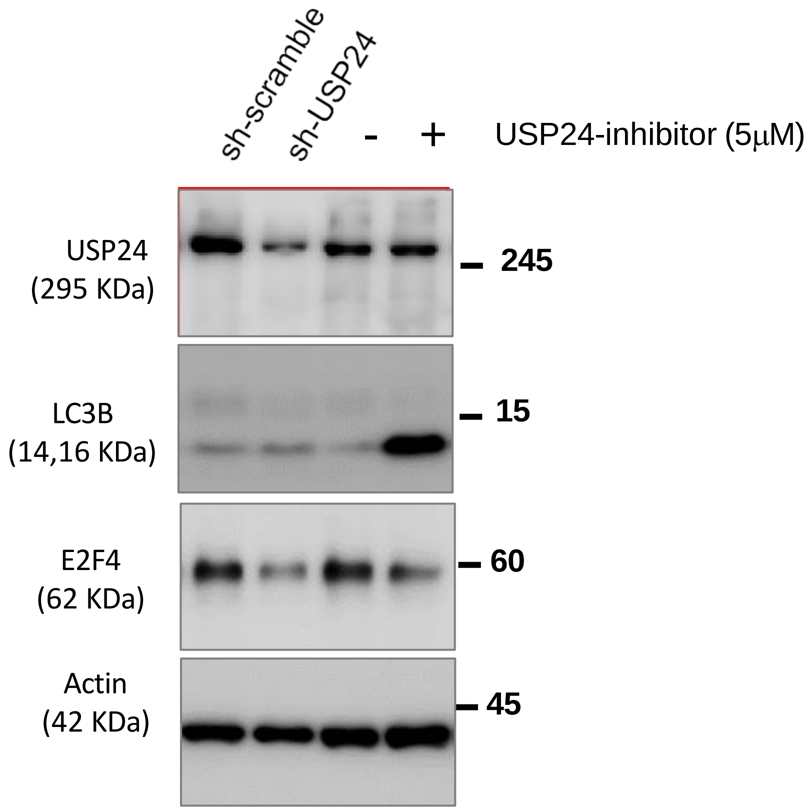
sh-scramble
sh-USP24
-
+
USP24-inhibitor (5µM)
USP24
(295 KDa)
245
LC3B
(14,16 KDa)
15
E2F4
(62 KDa)
60
Actin
(42 KDa)
45

1

Fig2.D(a)

PC9-GR

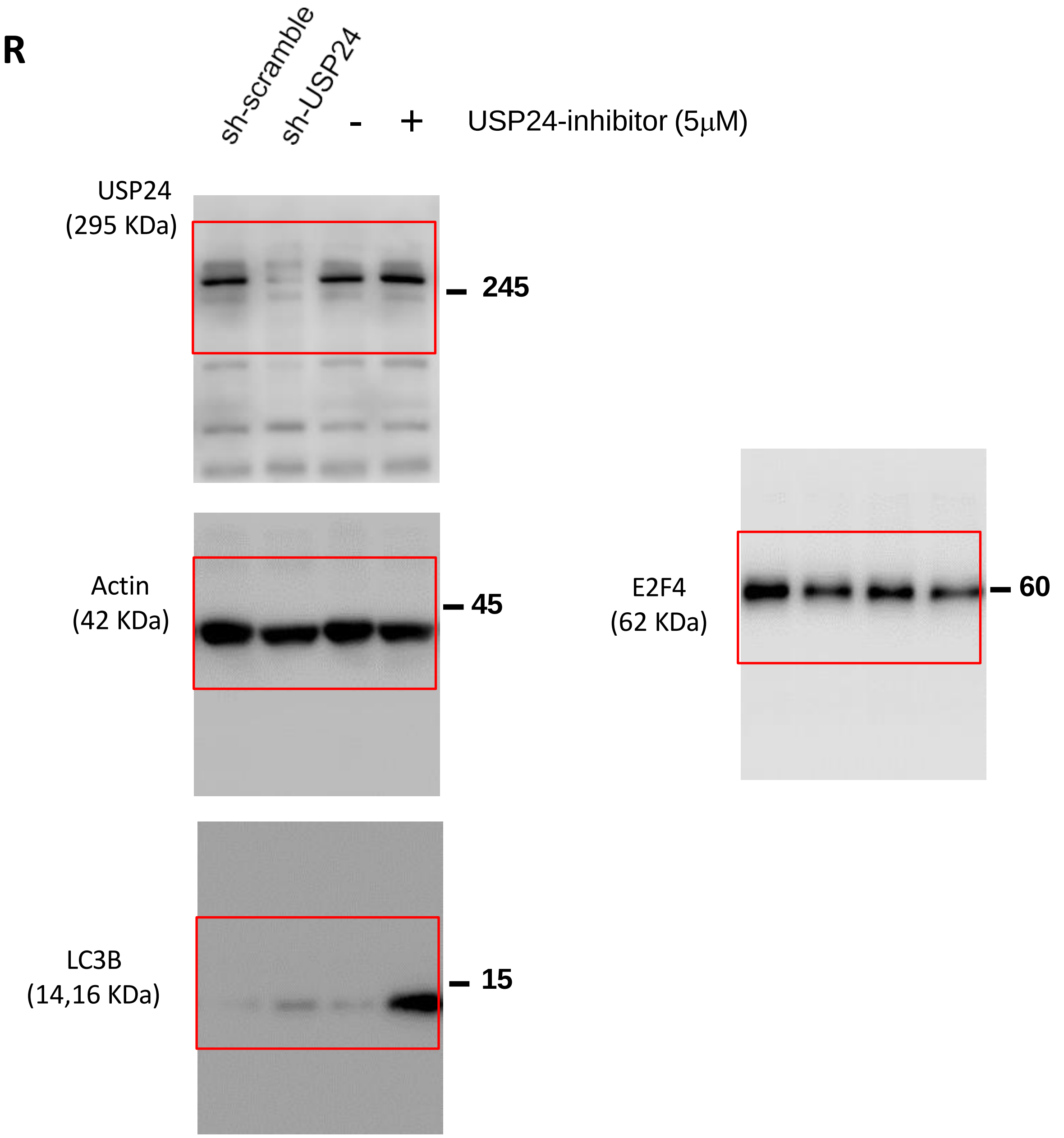

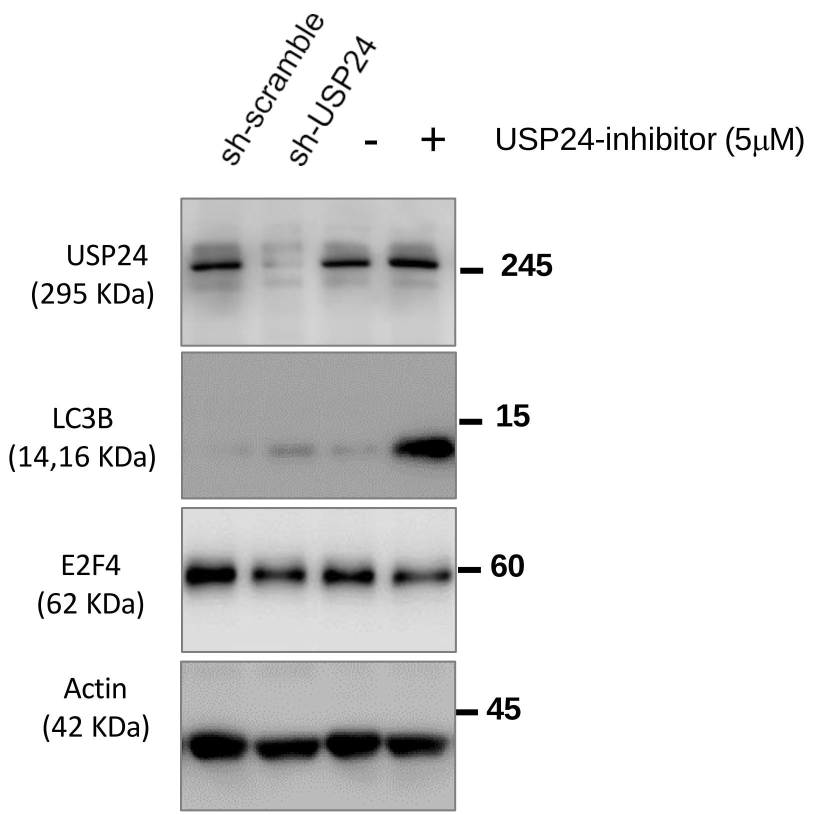

2

Fig2.D(a)

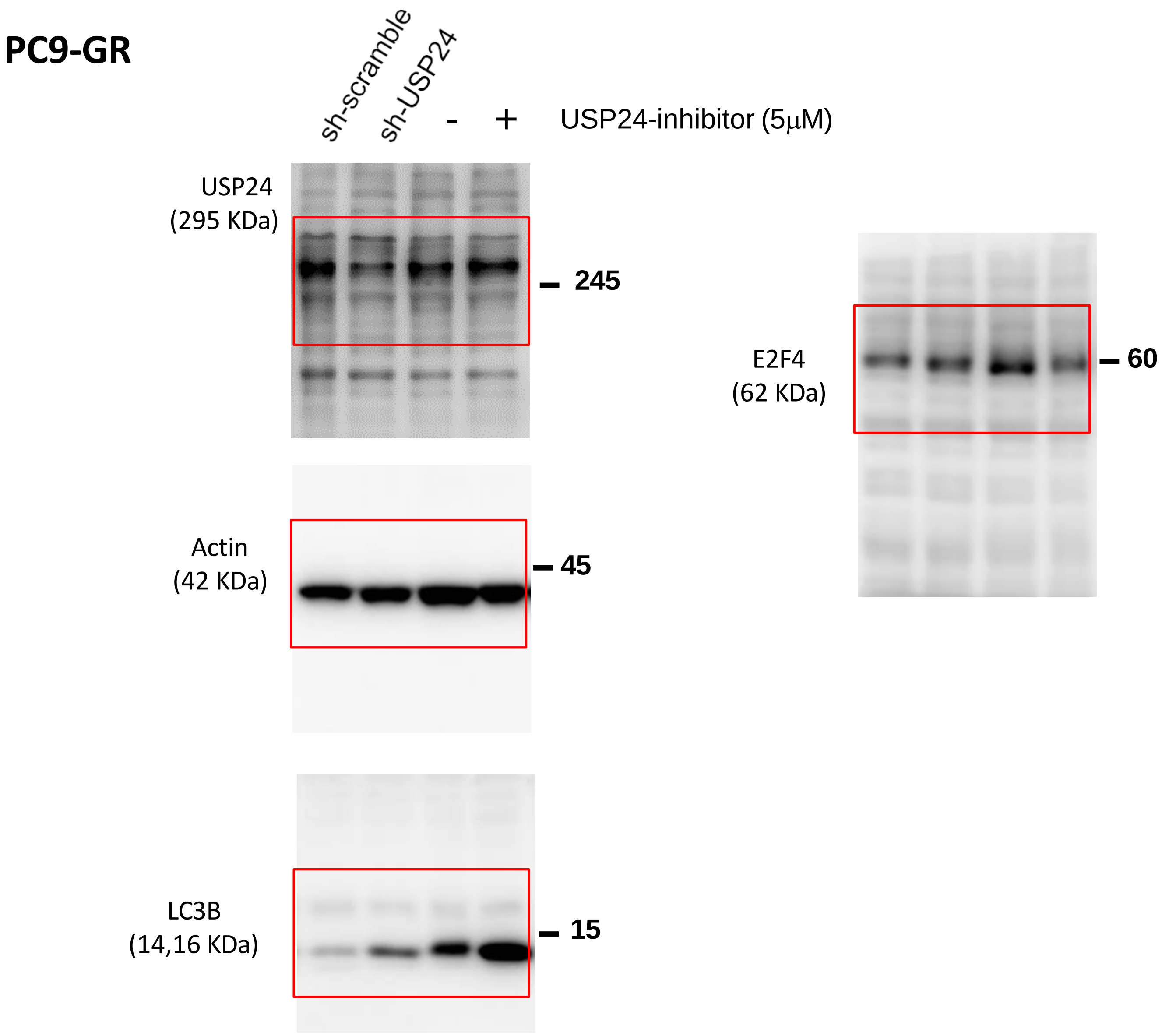
PC9-GR
sh-scramble
sh-USP24
-
+
USP24-inhibitor (5μM)
USP24
(295 KDa)
245
Actin
(42 KDa)
45
LC3B
(14,16 KDa)
15
E2F4
(62 KDa)
60

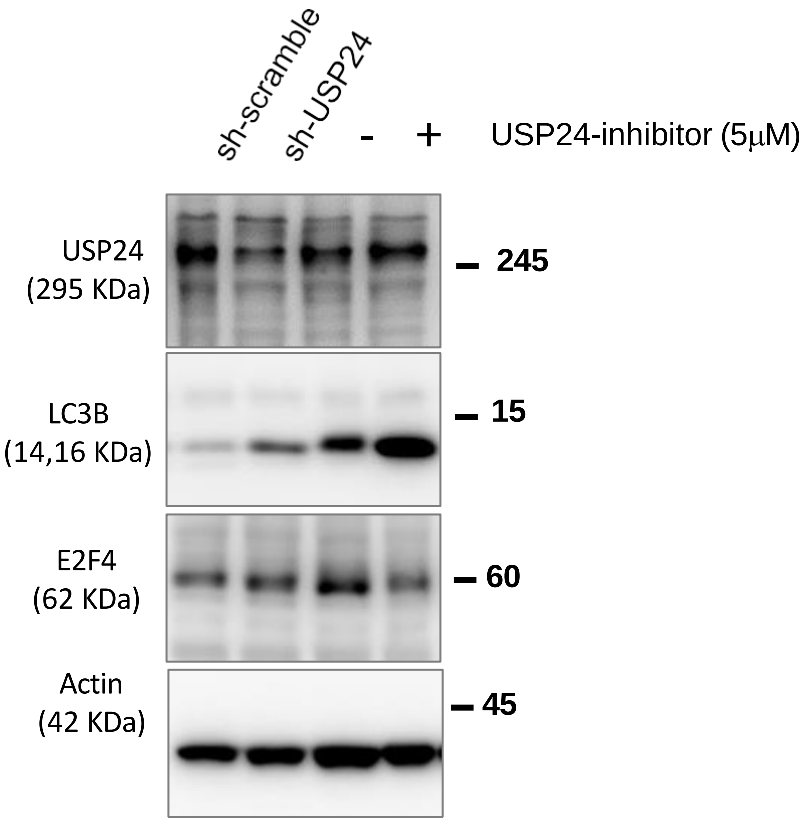
sh-scramble
sh-USP24
-
+
USP24-inhibitor (5μM)
USP24
(295 KDa)
245
LC3B
(14,16 KDa)
15
E2F4
(62 KDa)
60
Actin
(42 KDa)
45

3

Fig2.D(a)

PC9-GR

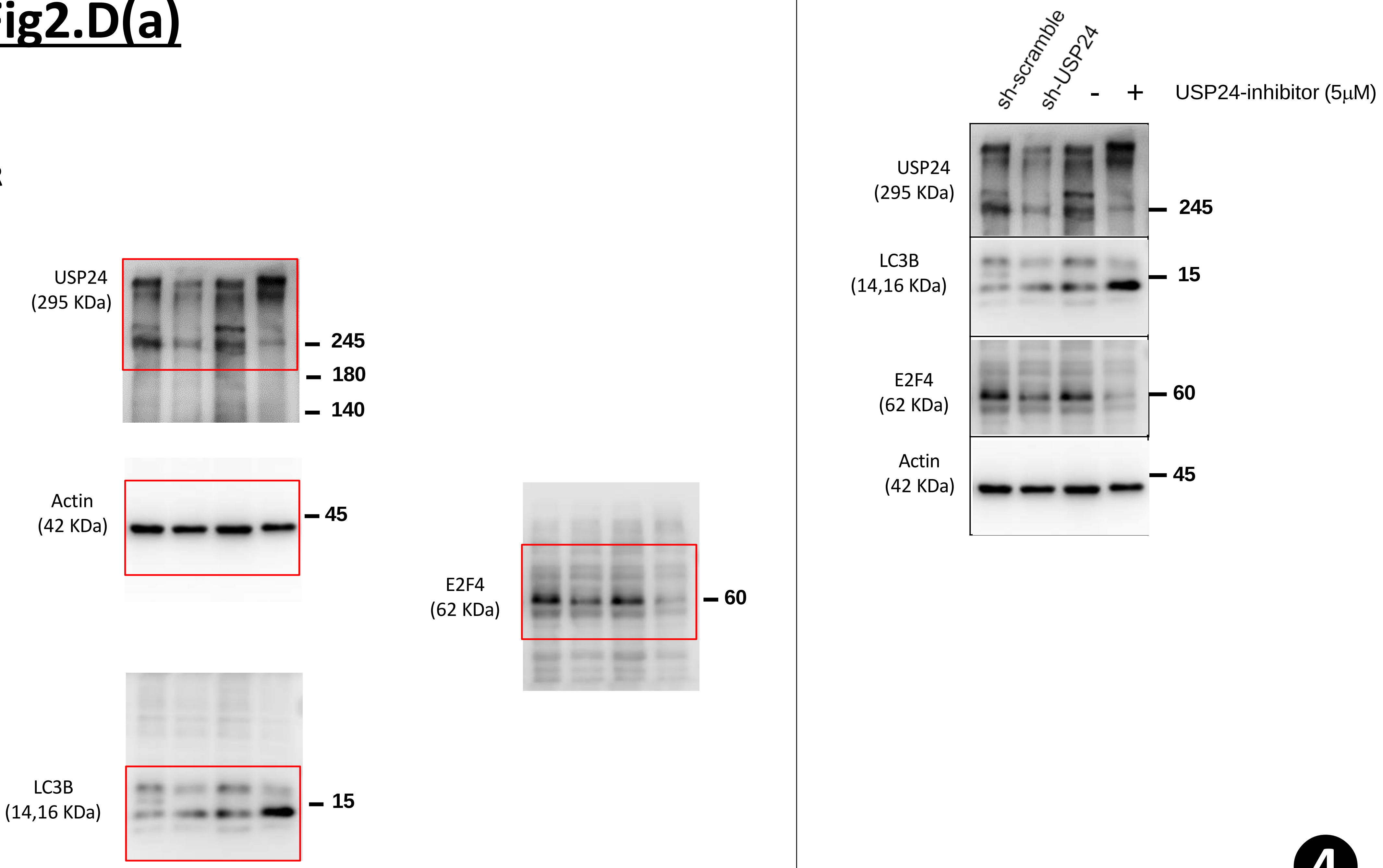

4

Fig2.E(a)

PC9-GR

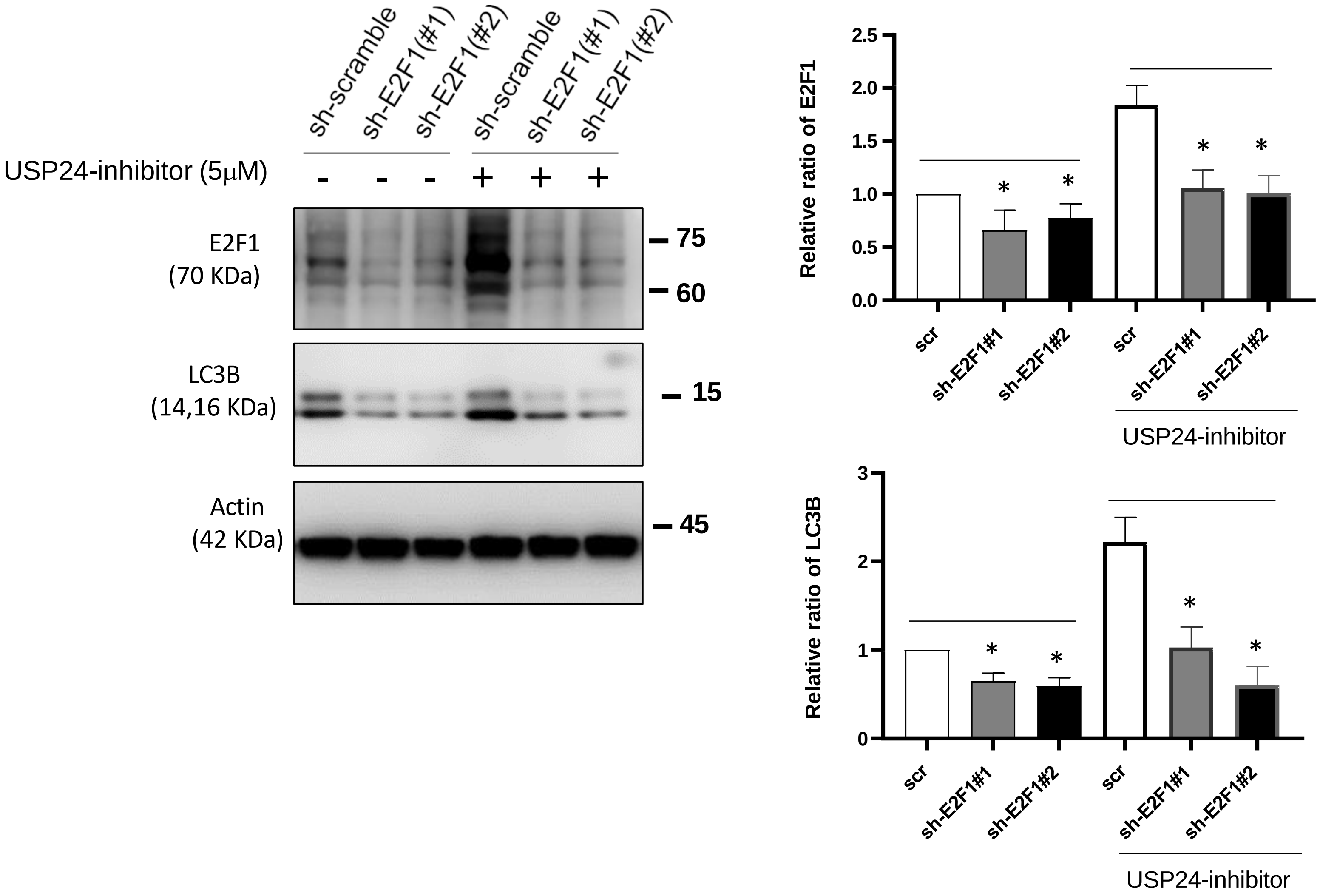

Fig2.E(a)

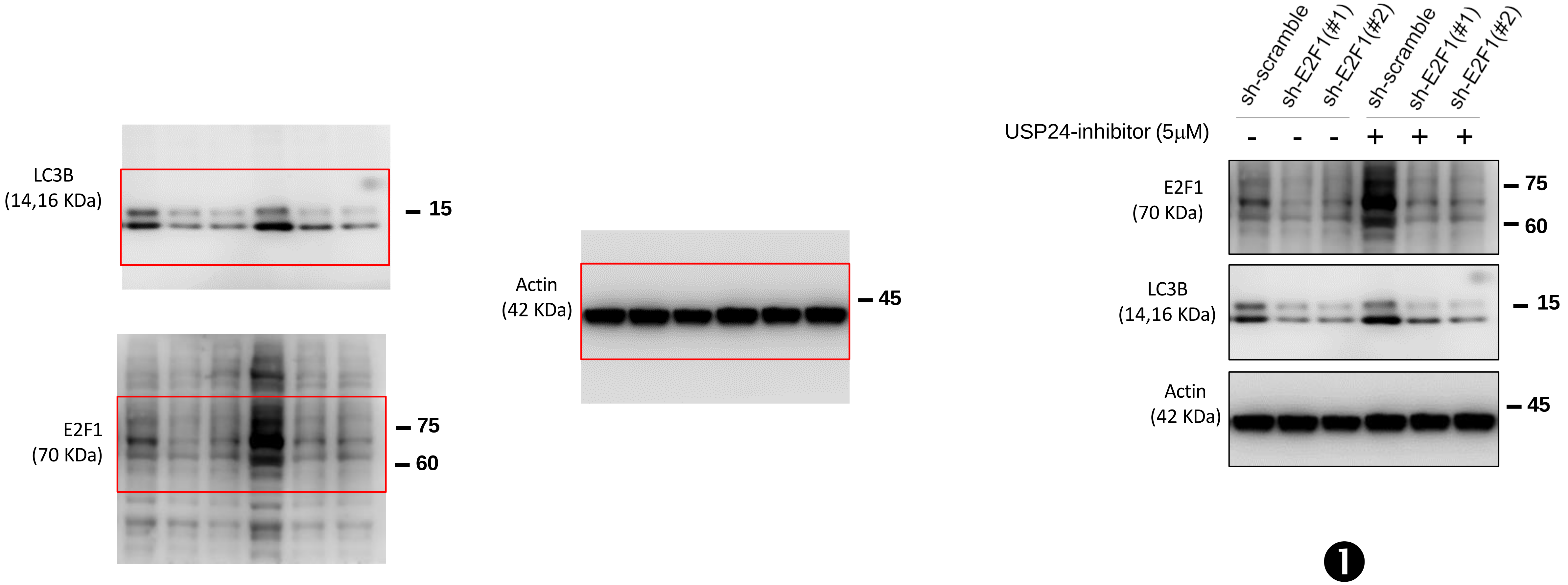
LC3B
(14,16 KDa)
15
E2F1
(70 KDa)
75
60
Actin
(42 KDa)
45
sh-scramble
sh-E2F1(#1)
sh-E2F1(#2)
sh-scramble
sh-E2F1(#1)
sh-E2F1(#2)
USP24-inhibitor (5μM)
- - - + + +
E2F1
(70 KDa)
75
60
LC3B
(14,16 KDa)
15
Actin
(42 KDa)
45
❶

Fig2.E(a)

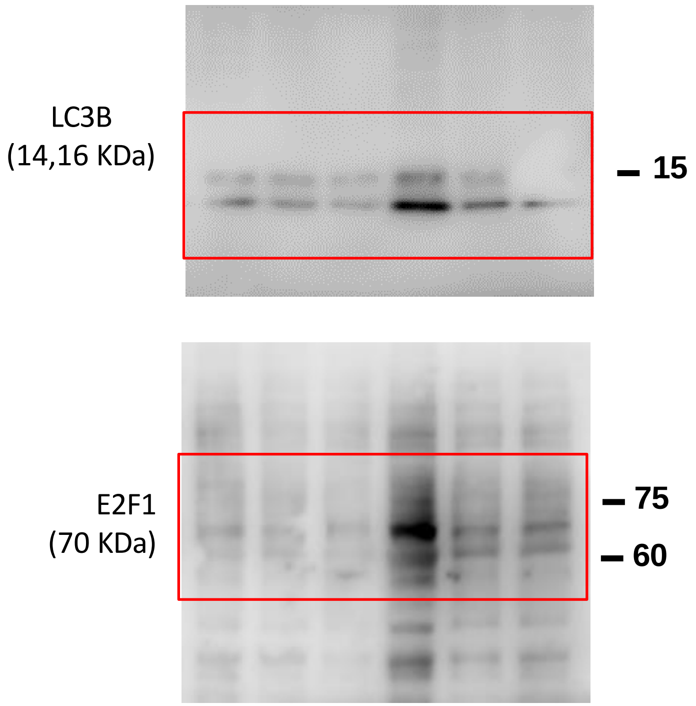
LC3B
(14,16 KDa)
15
E2F1
(70 KDa)
75
60

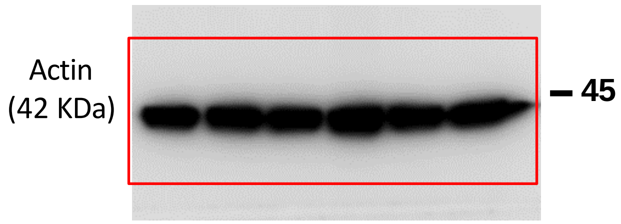
Actin
(42 KDa)
45

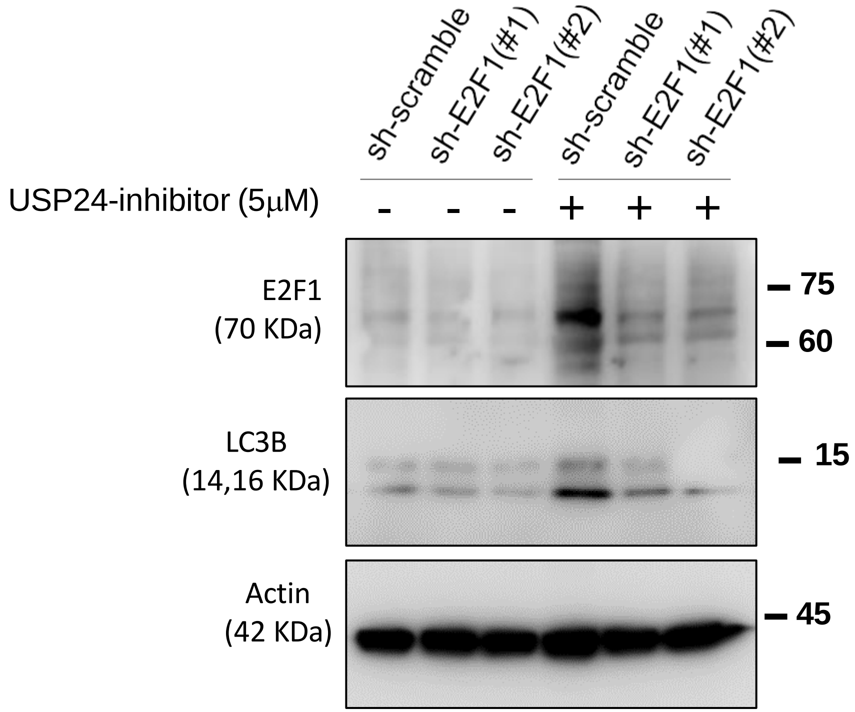
sh-scramble
sh-E2F1(#1)
sh-E2F1(#2)
sh-scramble
sh-E2F1(#1)
sh-E2F1(#2)
USP24-inhibitor (5μM)
- - - + + +
E2F1
(70 KDa)
75
60
LC3B
(14,16 KDa)
15
Actin
(42 KDa)
45

❷

Fig2.E(a)

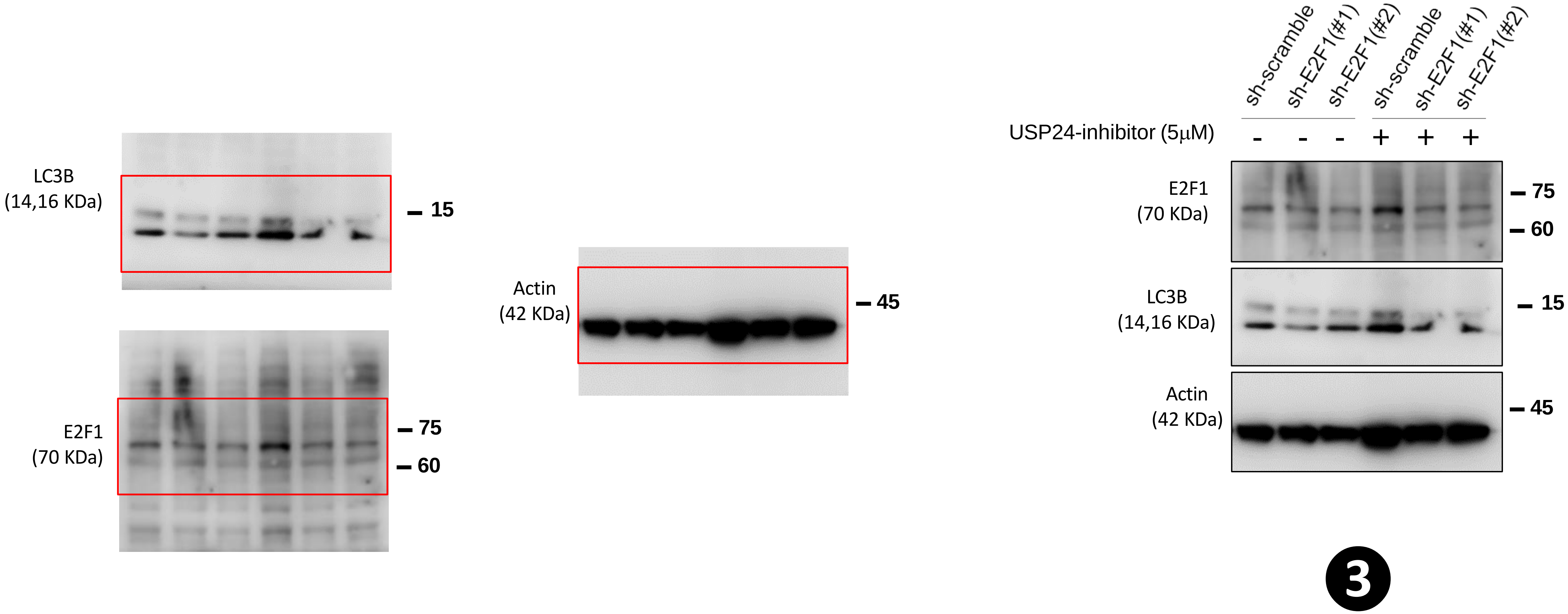

LC3B
(14,16 KDa)
15
E2F1
(70 KDa)
75
60
Actin
(42 KDa)
45
sh-scramble
sh-E2F1(#1)
sh-E2F1(#2)
sh-scramble
sh-E2F1(#1)
sh-E2F1(#2)
USP24-inhibitor (5μM)
- - - + + +
E2F1
(70 KDa)
75
60
LC3B
(14,16 KDa)
15
Actin
(42 KDa)
45
3

Fig2.F

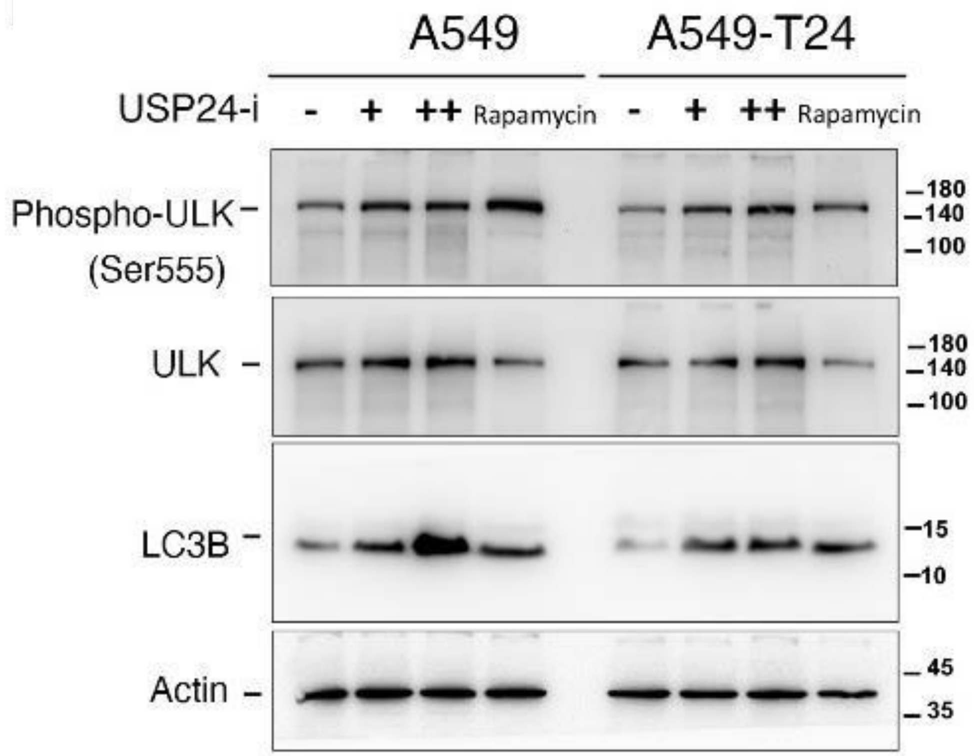
A549
A549-T24
USP24-i - + ++ Rapamycin - + ++ Rapamycin
Phospho-ULK
(Ser555)
ULK
LC3B
Actin
180
140
100
180
140
100
15
10
45
35

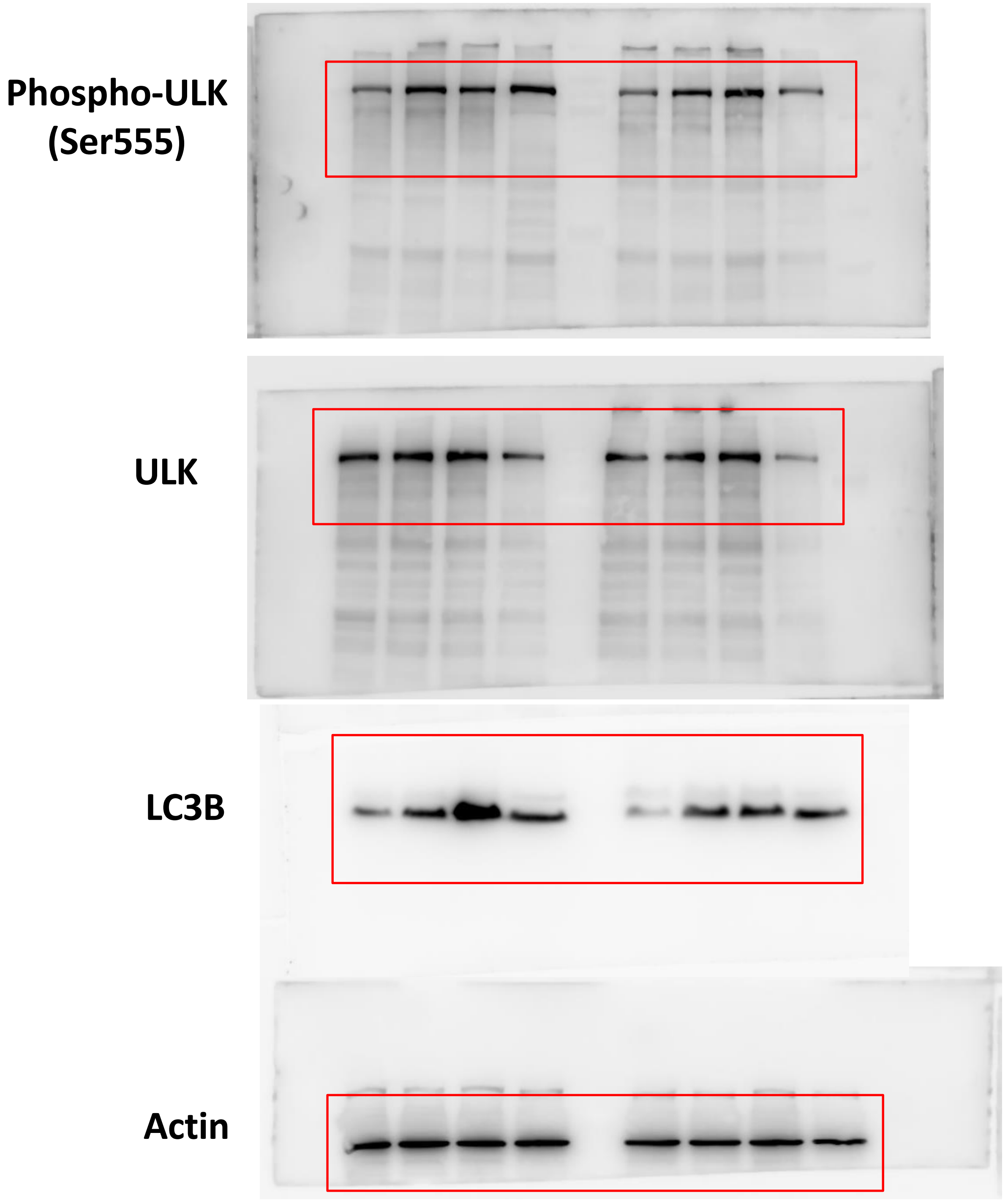
Phospho-ULK
(Ser555)
ULK
LC3B
Actin

Fig3.B

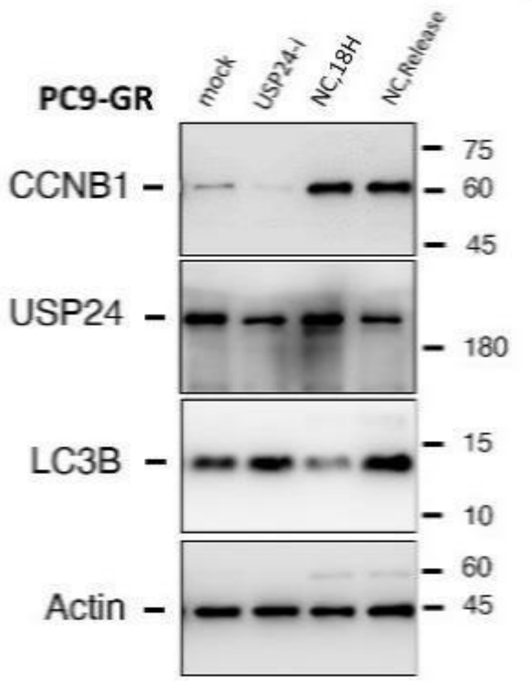
PC9-GR
mock
USP24-i
NC,18H
NC,Release
CCNB1
USP24
LC3B
Actin
75
60
45
180
15
10
60
45

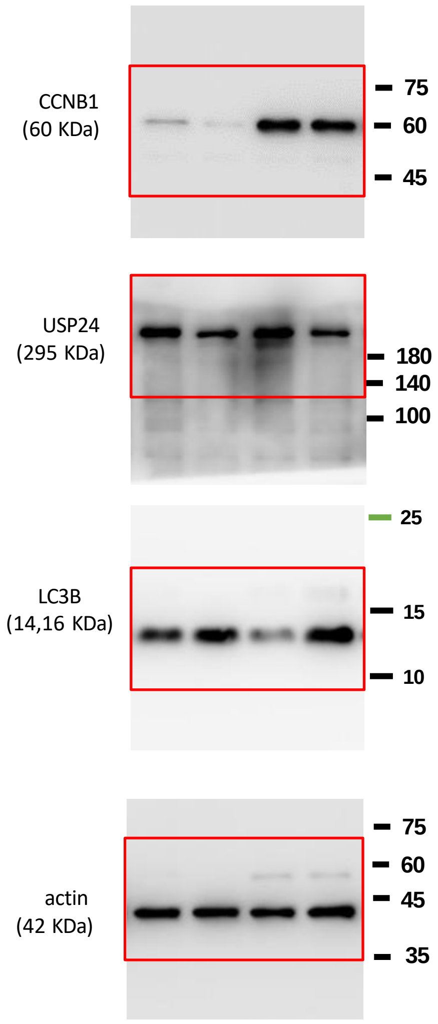
CCNB1
(60 KDa)
75
60
45
USP24
(295 KDa)
180
140
100
25
LC3B
(14,16 KDa)
15
10
actin
(42 KDa)
75
60
45
35

Fig3.C

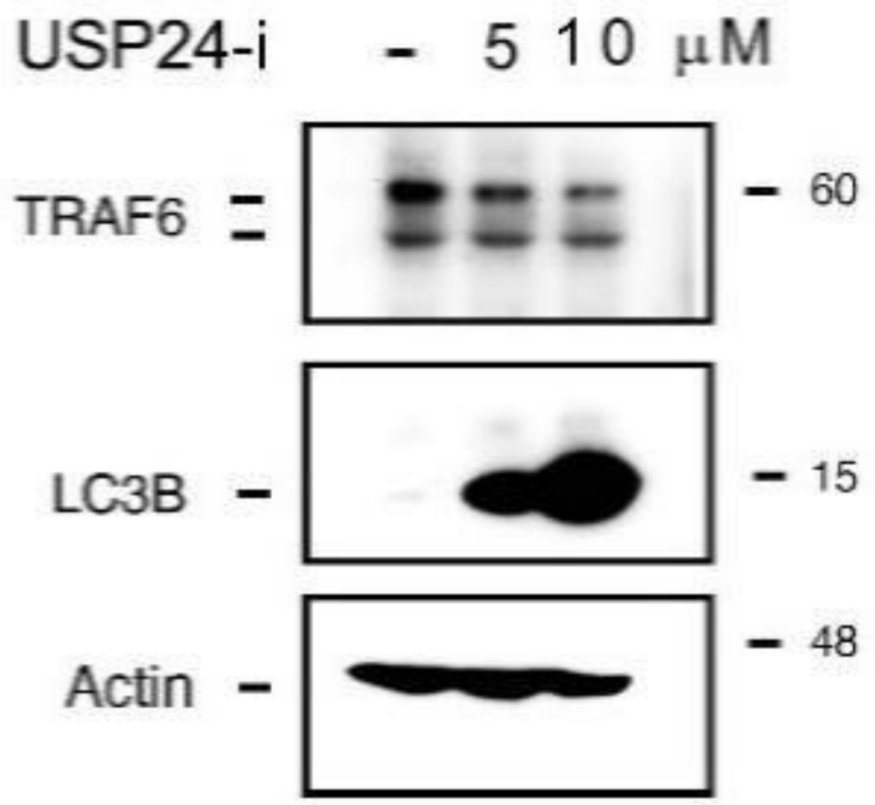

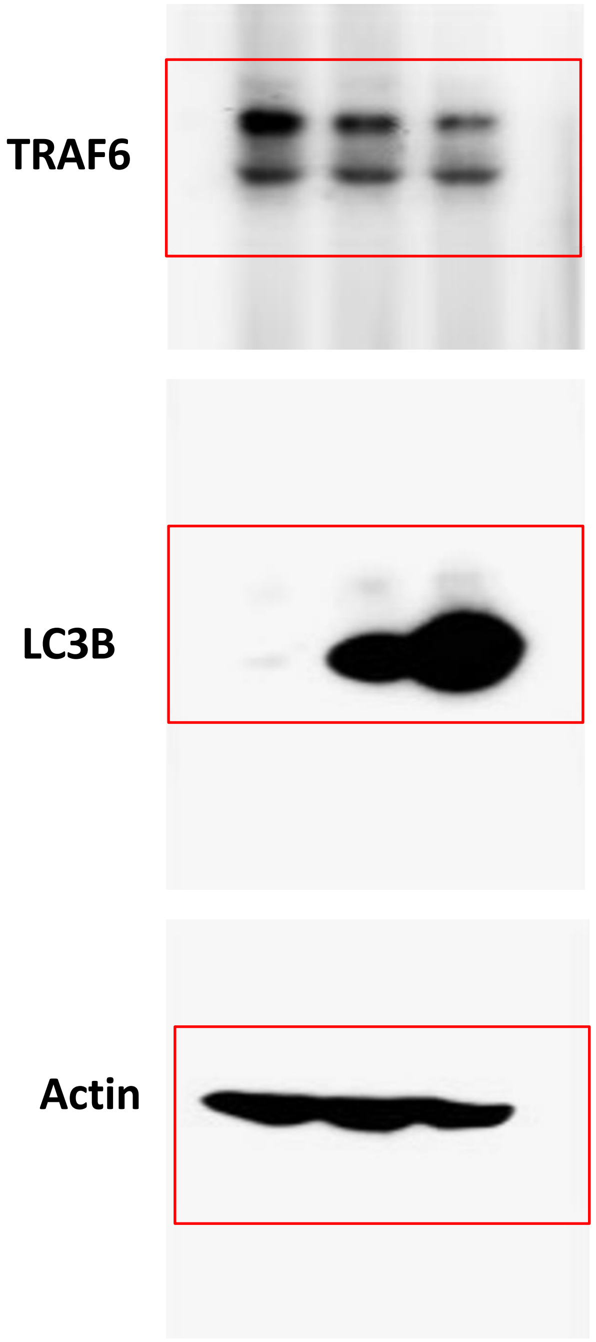

Fig3.D(a)

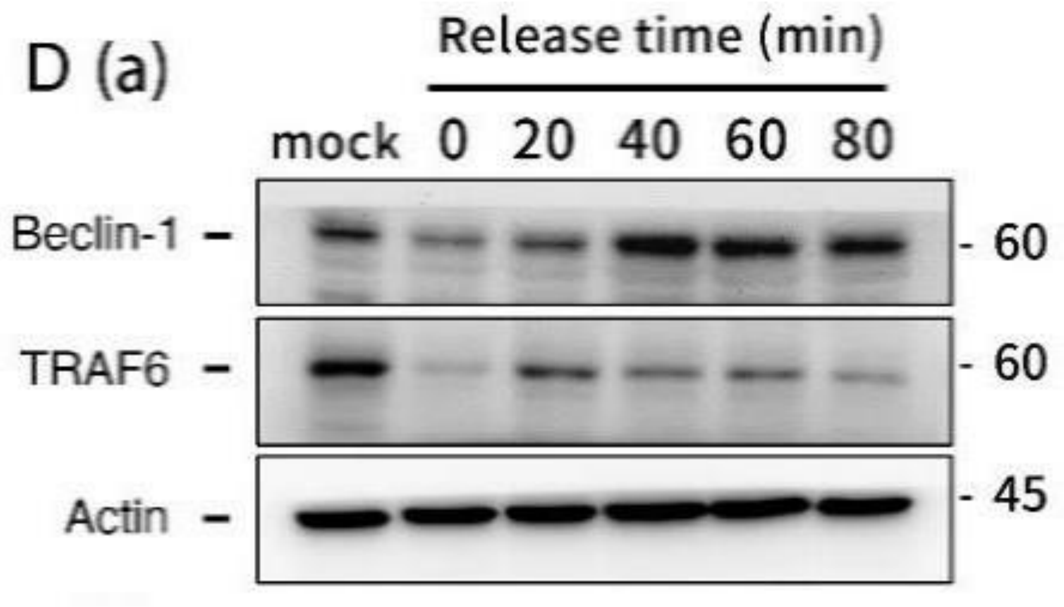

D (a)
Release time (min)
mock 0 20 40 60 80
Beclin-1
TRAF6
Actin
60
60
45

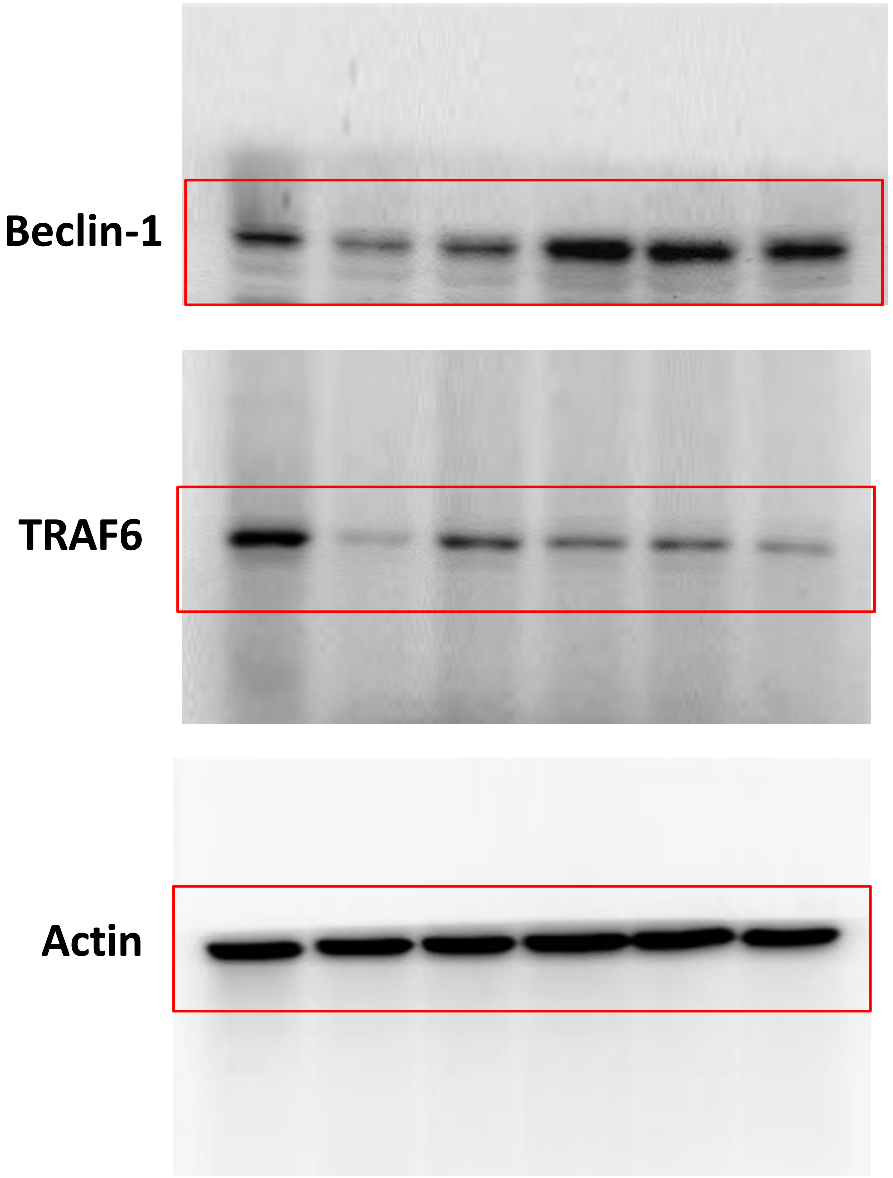

Beclin-1
TRAF6
Actin

Fig3.E(a)

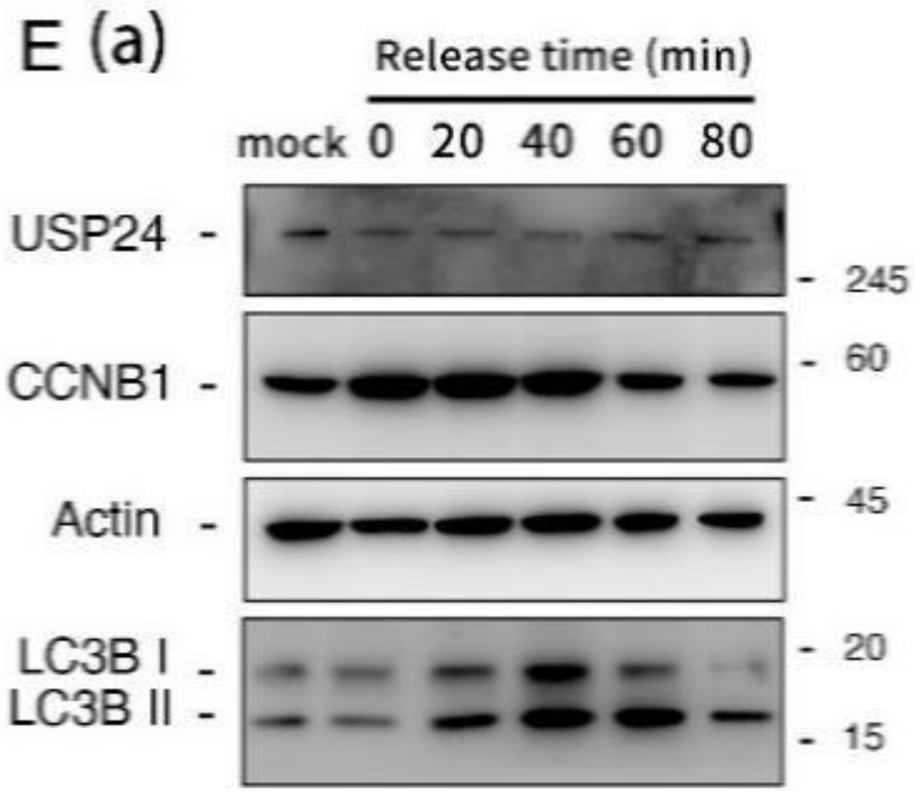

E (a)
Release time (min)
mock 0 20 40 60 80
USP24
CCNB1
Actin
LC3B I
LC3B II
245
60
45
20
15

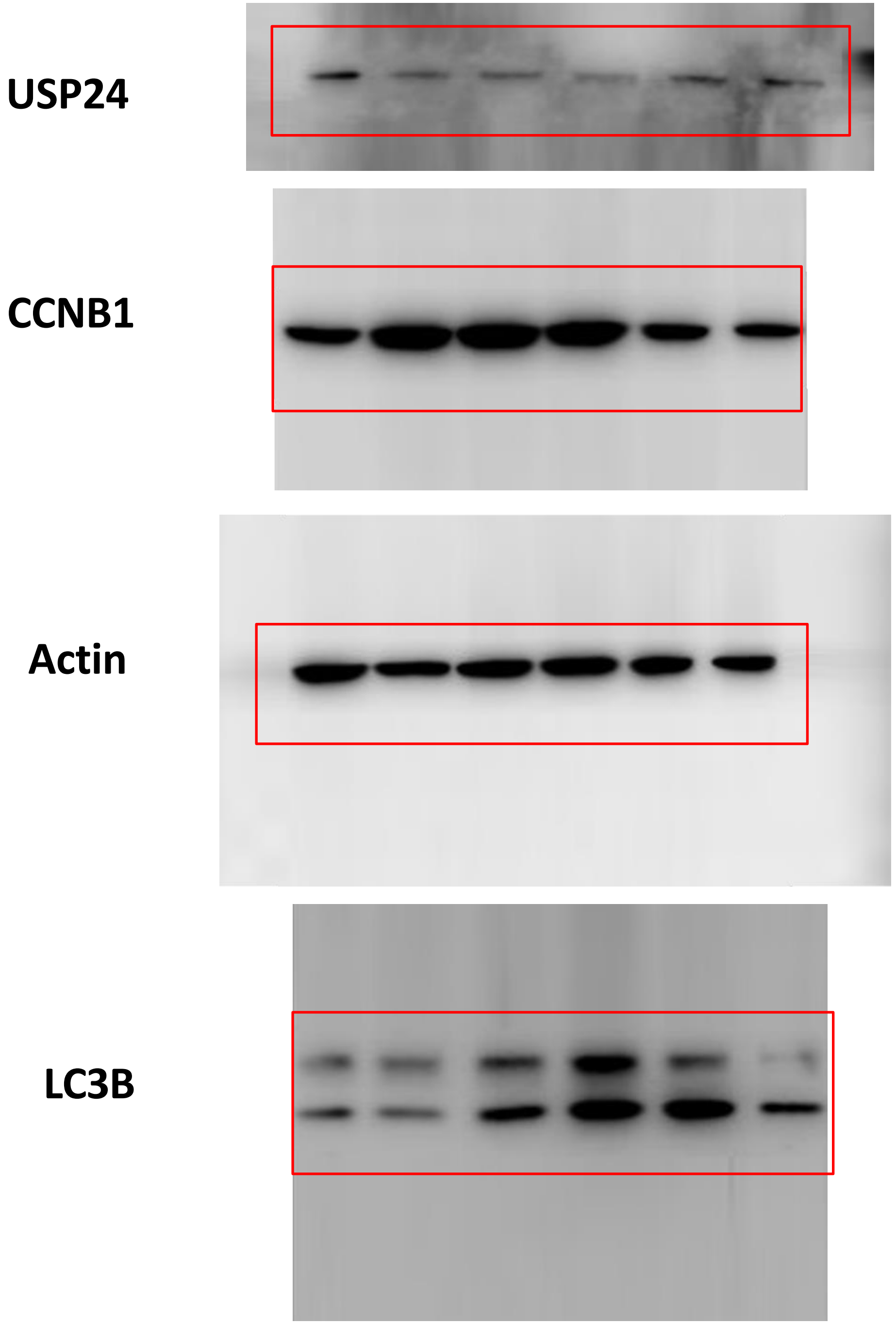

USP24
CCNB1
Actin
LC3B

Fig3.E(b)

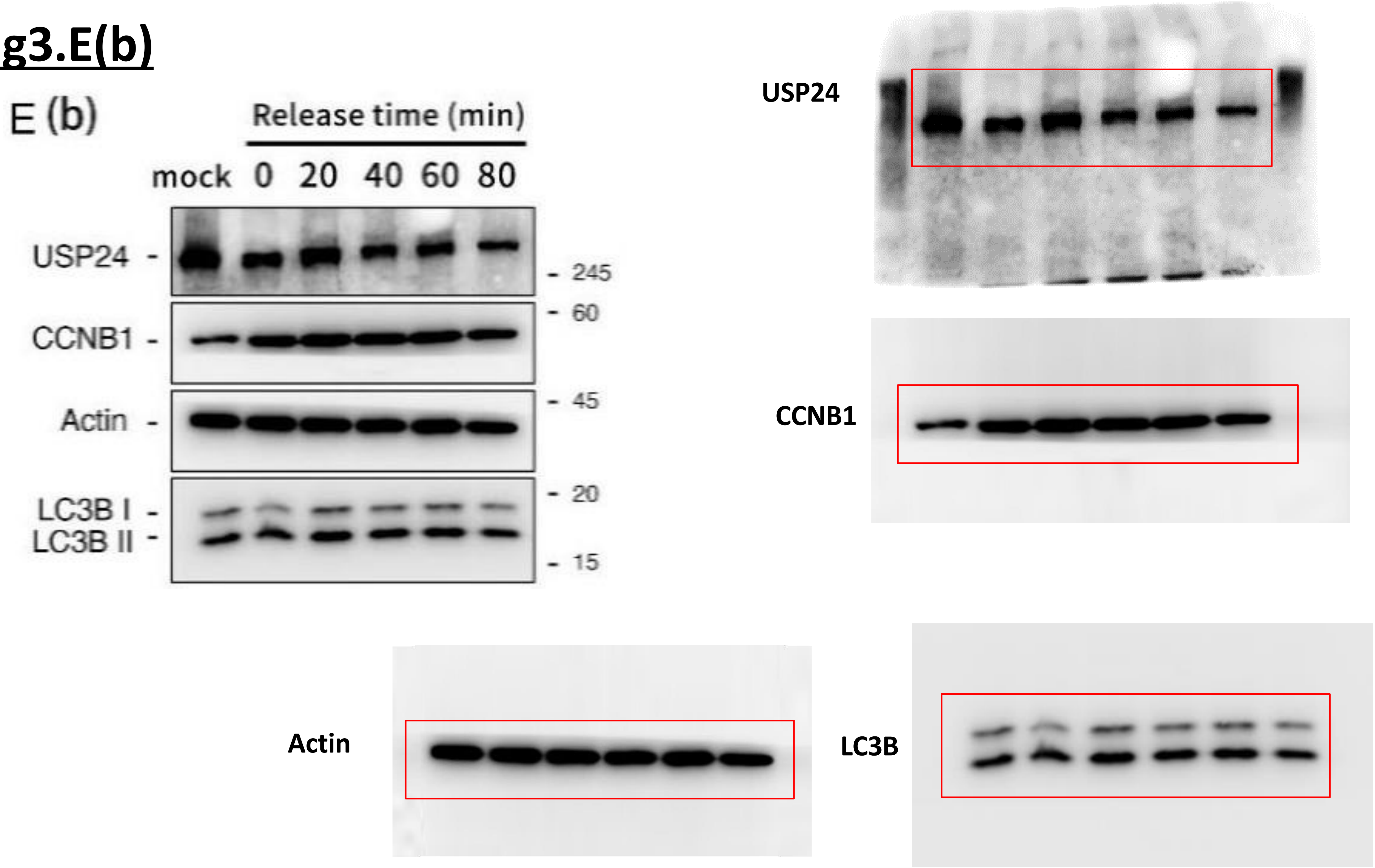
E (b)
Release time (min)
mock
0
20
40
60
80
USP24
CCNB1
Actin
LC3B I
LC3B II
245
60
45
20
15
USP24
CCNB1
Actin
LC3B

Fig3.F

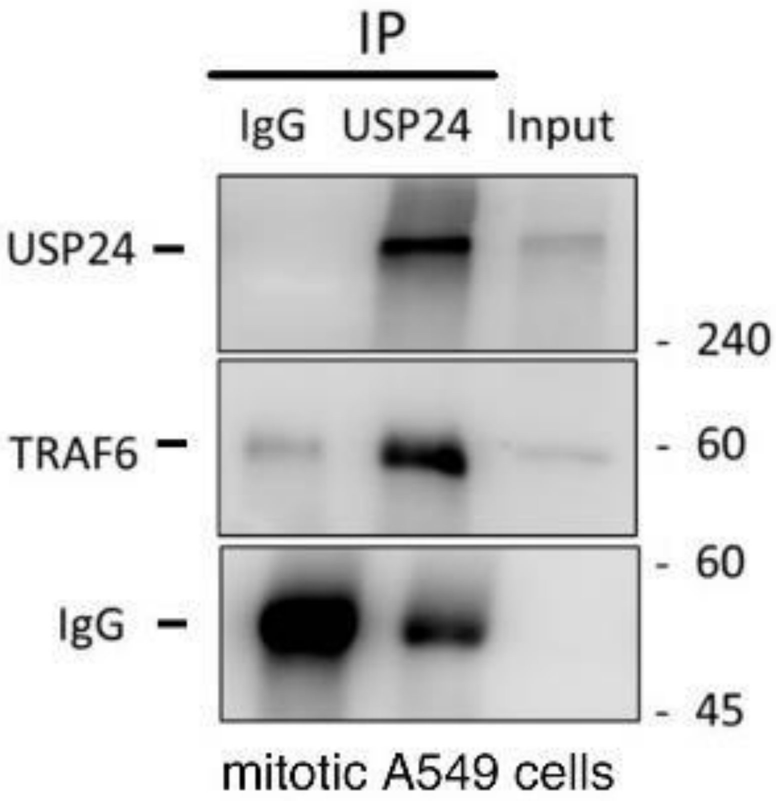

IP
IgG
USP24
Input
USP24
TRAF6
IgG
240
60
60
45
mitotic A549 cells

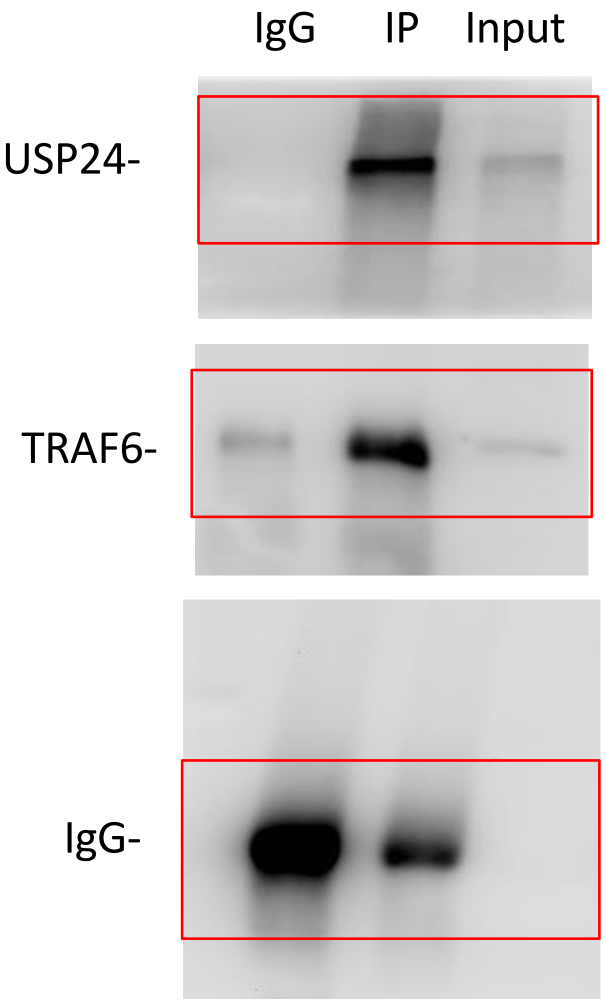

IgG
IP
Input
USP24-
TRAF6-
IgG-

Fig.3.G(a)

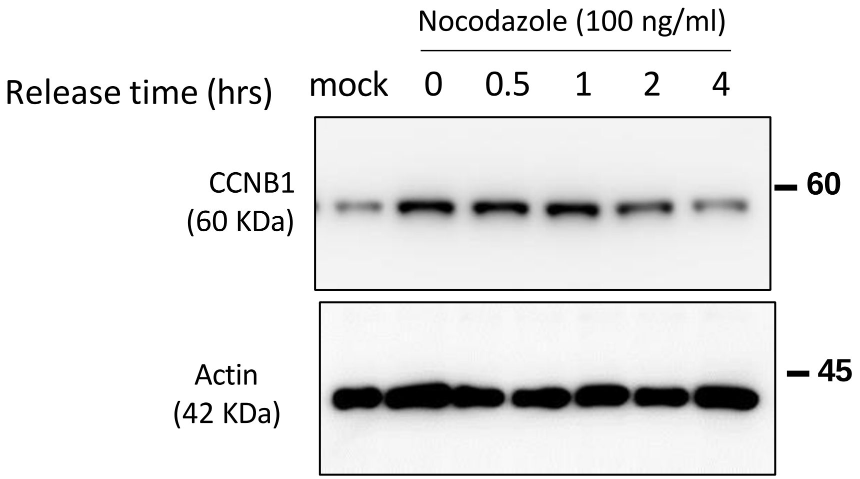
Nocodazole (100 ng/ml)
Release time (hrs)
mock
0
0.5
1
2
4
CCNB1
(60 KDa)
60
Actin
(42 KDa)
45

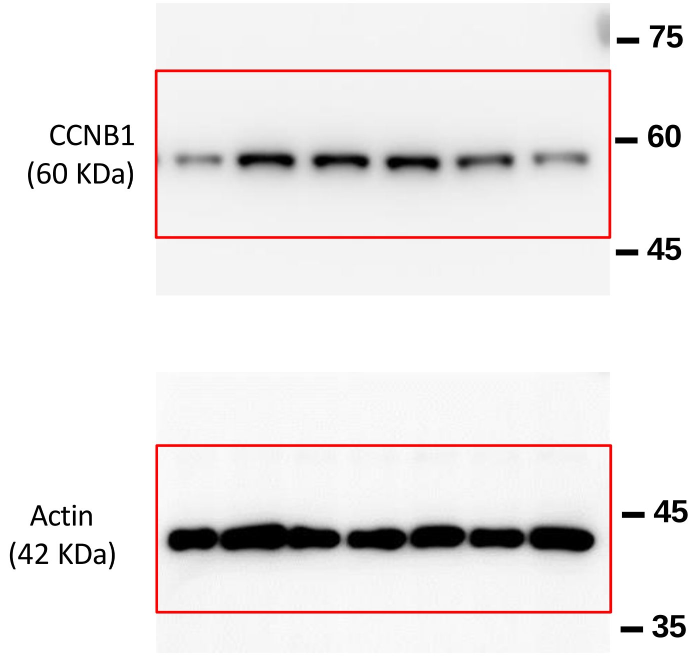
75
CCNB1
(60 KDa)
60
45
Actin
(42 KDa)
45
35

Fig.3.G(b)

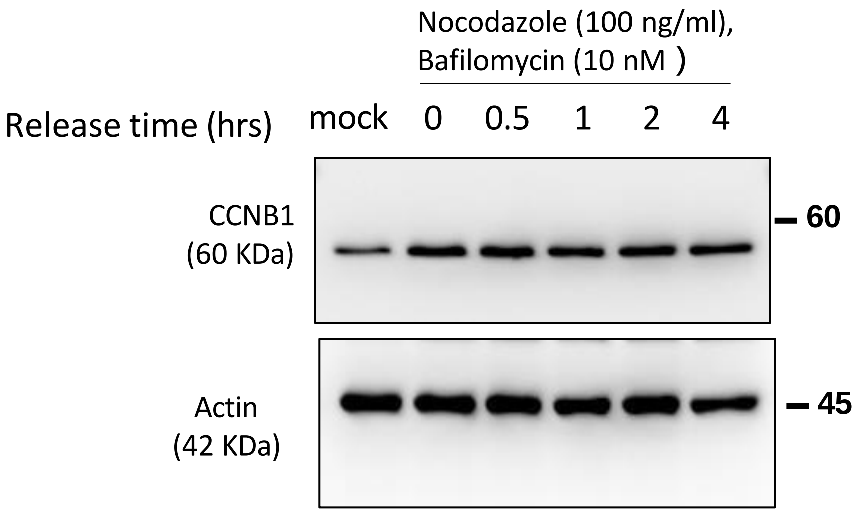

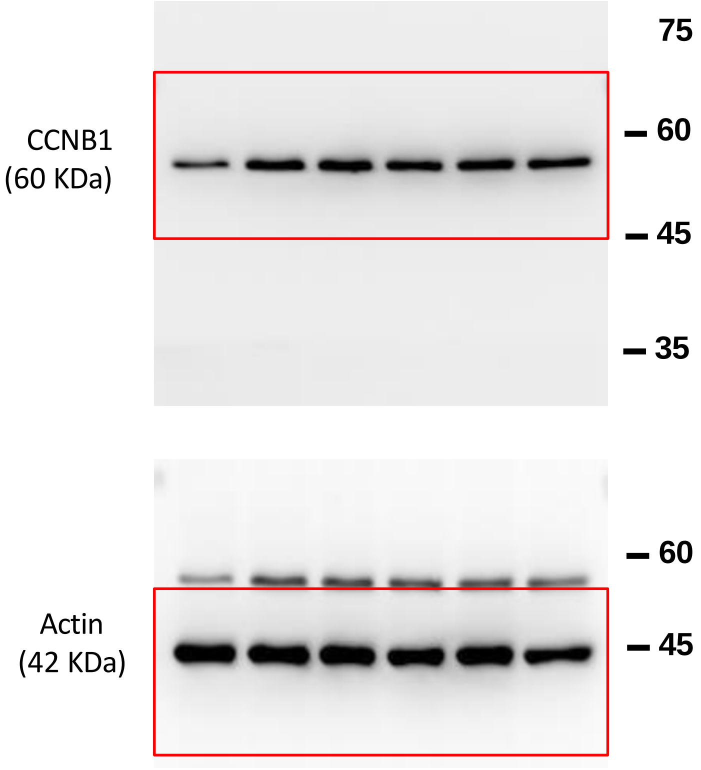

Bafilomycin : autophagy inhibitor

Fig.3.H

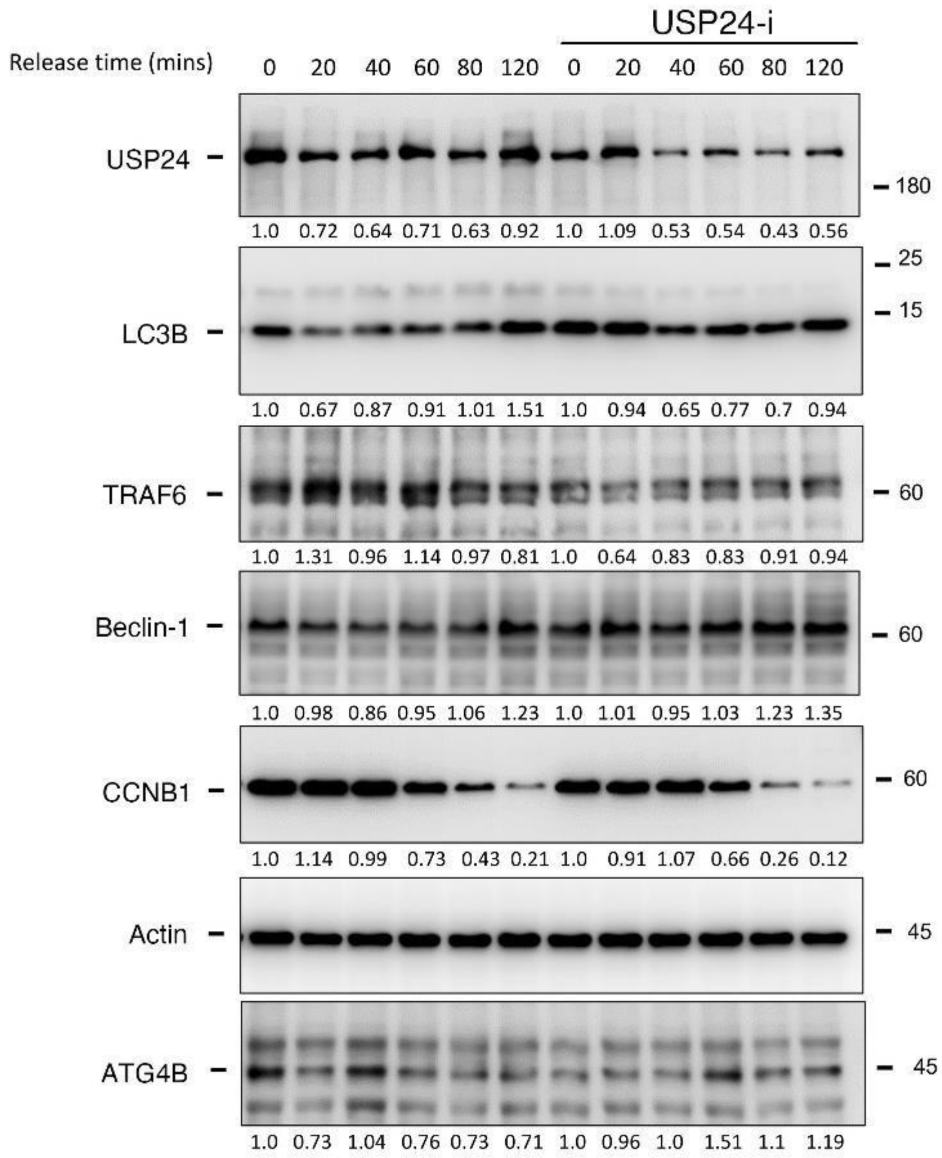

USP24-i
Release time (mins)
0 20 40 60 80 120 0 20 40 60 80 120
USP24
180
1.0 0.72 0.64 0.71 0.63 0.92 1.0 1.09 0.53 0.54 0.43 0.56
25
15
LC3B
1.0 0.67 0.87 0.91 1.01 1.51 1.0 0.94 0.65 0.77 0.7 0.94
TRAF6
60
1.0 1.31 0.96 1.14 0.97 0.81 1.0 0.64 0.83 0.83 0.91 0.94
Beclin-1
60
1.0 0.98 0.86 0.95 1.06 1.23 1.0 1.01 0.95 1.03 1.23 1.35
CCNB1
60
1.0 1.14 0.99 0.73 0.43 0.21 1.0 0.91 1.07 0.66 0.26 0.12
Actin
45
ATG4B
45
1.0 0.73 1.04 0.76 0.73 0.71 1.0 0.96 1.0 1.51 1.1 1.19

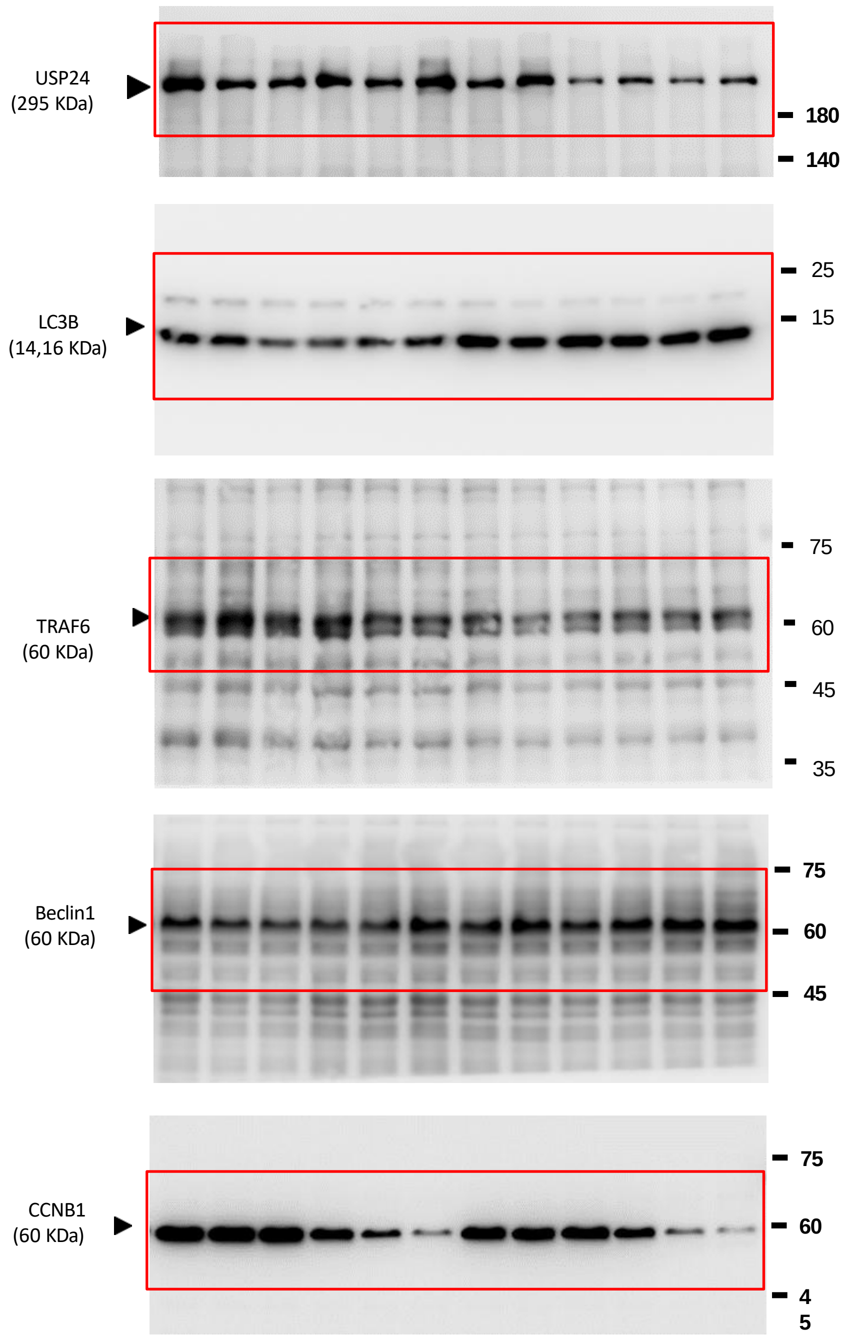

USP24
(295 KDa)
180
140
LC3B
(14,16 KDa)
25
15
TRAF6
(60 KDa)
75
60
45
35
Beclin1
(60 KDa)
75
60
45
CCNB1
(60 KDa)
75
60
4
5

Fig.3.H

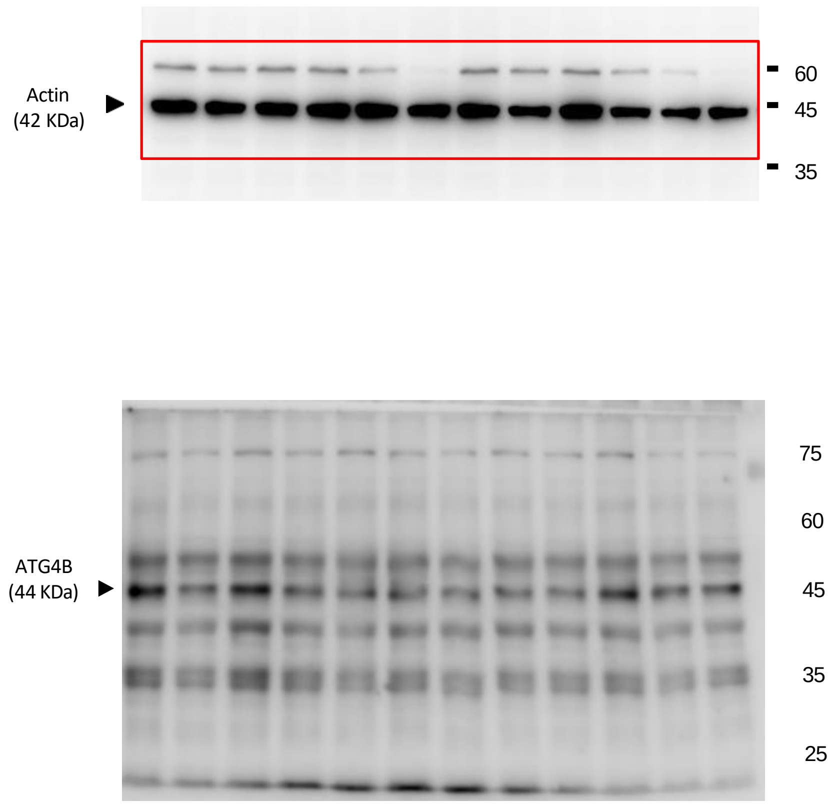

Fig4.B(a)

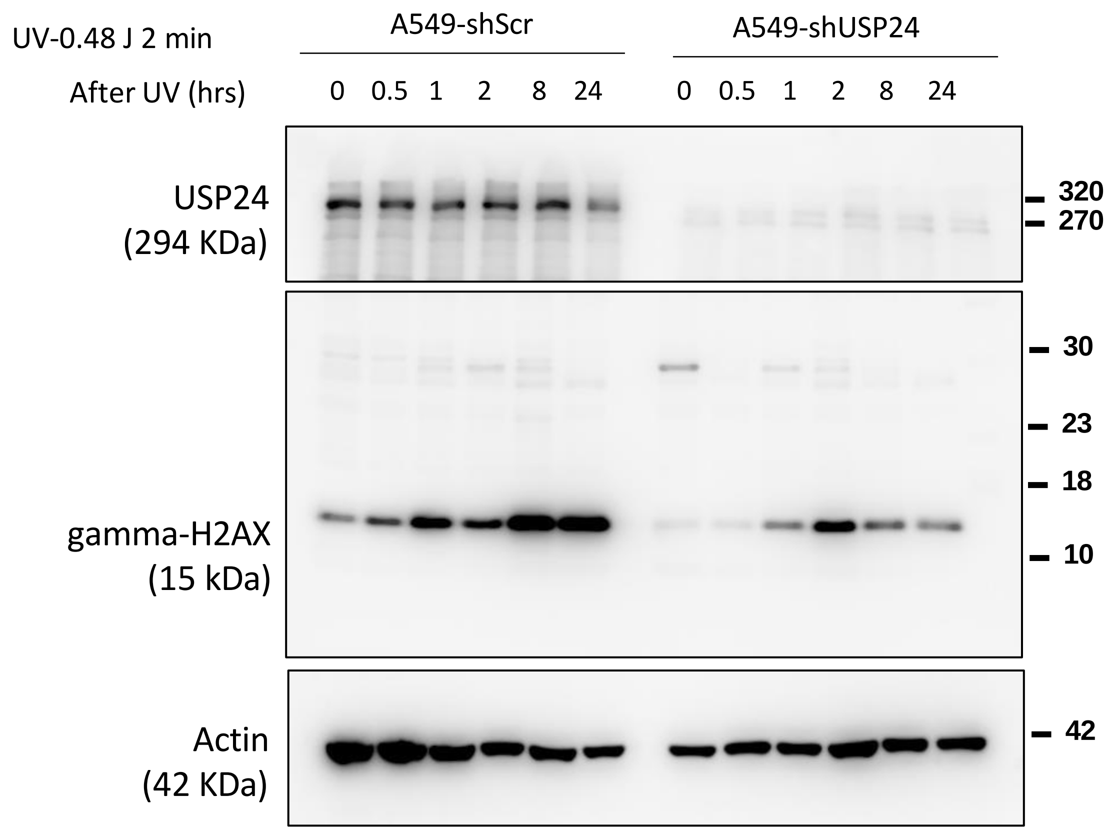
UV-0.48 J 2 min
After UV (hrs)
A549-shScr
A549-shUSP24
0 0.5 1 2 8 24
0 0.5 1 2 8 24
USP24
(294 KDa)
320
270
gamma-H2AX
(15 kDa)
30
23
18
10
Actin
(42 KDa)
42

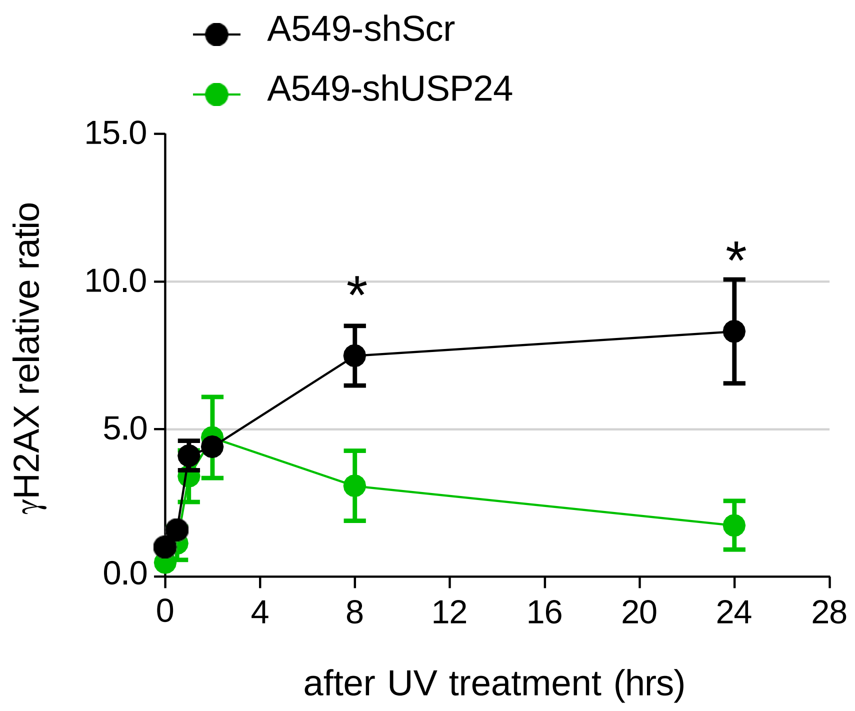
A549-shScr
A549-shUSP24
γH2AX relative ratio
15.0
10.0
5.0
0.0
*
*
0
4
8
12
16
20
24
28
after UV treatment (hrs)

Fig4.B(a)

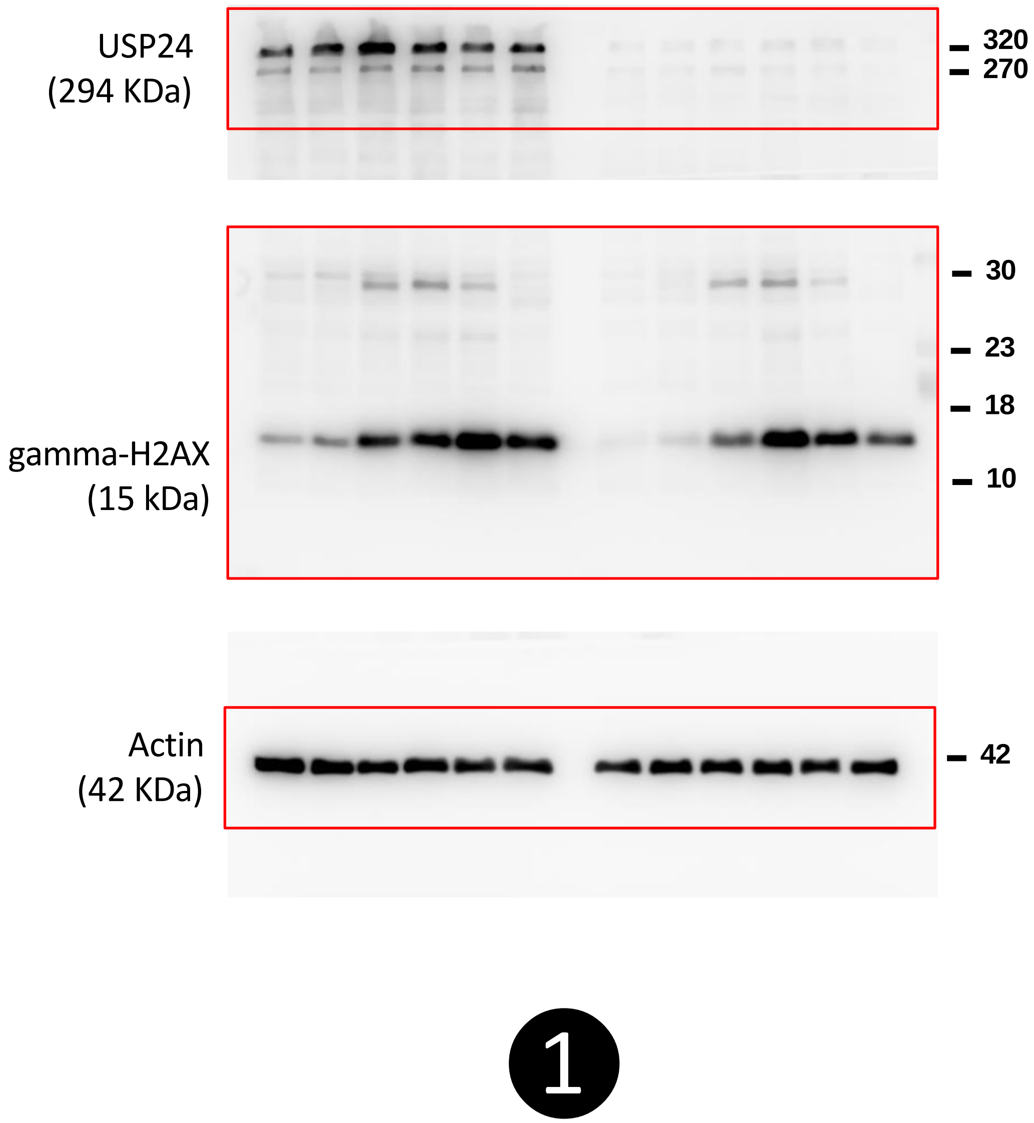
USP24
(294 KDa)
320
270
gamma-H2AX
(15 kDa)
30
23
18
10
Actin
(42 KDa)
42
1

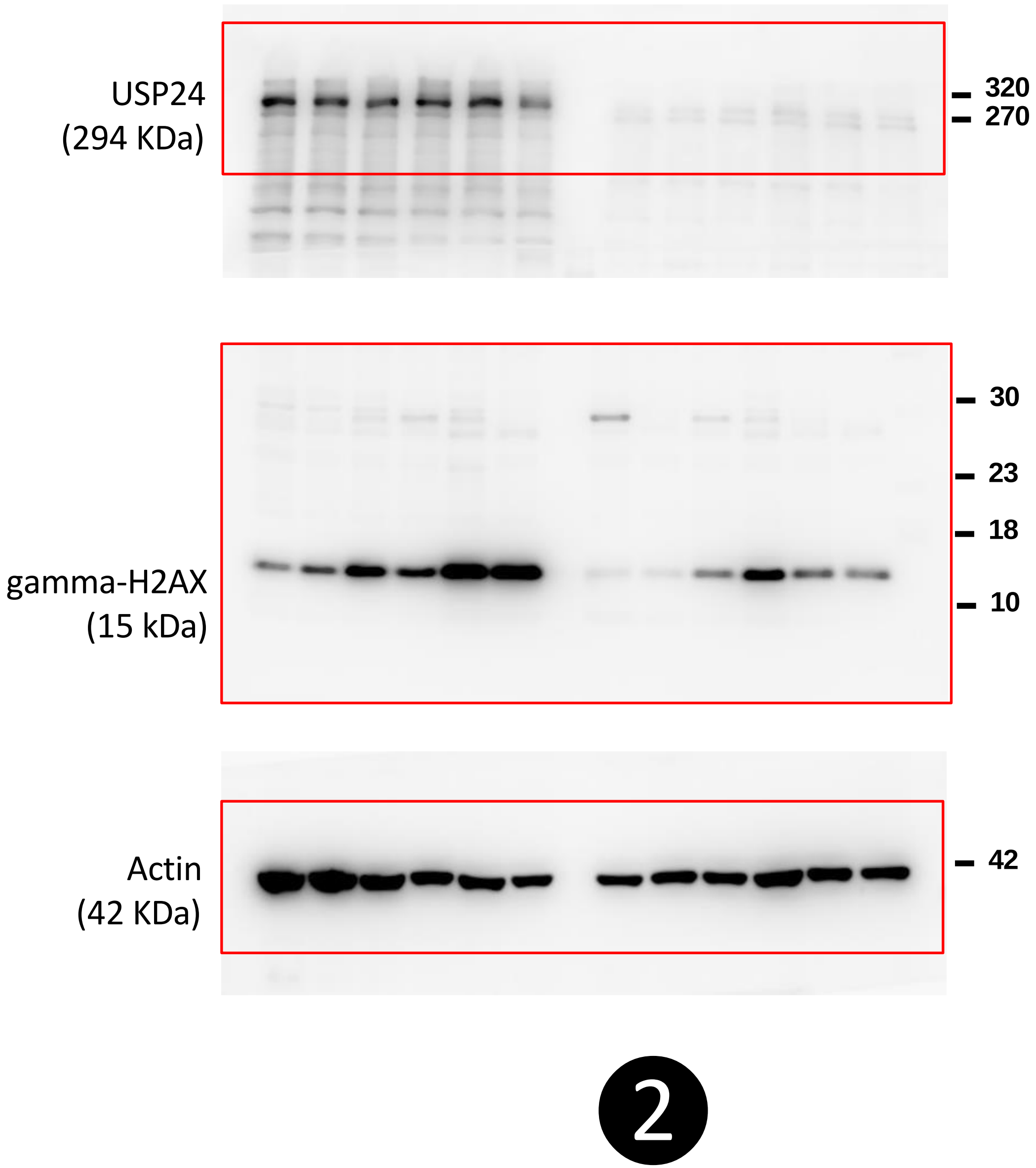
USP24
(294 KDa)
320
270
gamma-H2AX
(15 kDa)
30
23
18
10
Actin
(42 KDa)
42
2

Fig4.B(a)

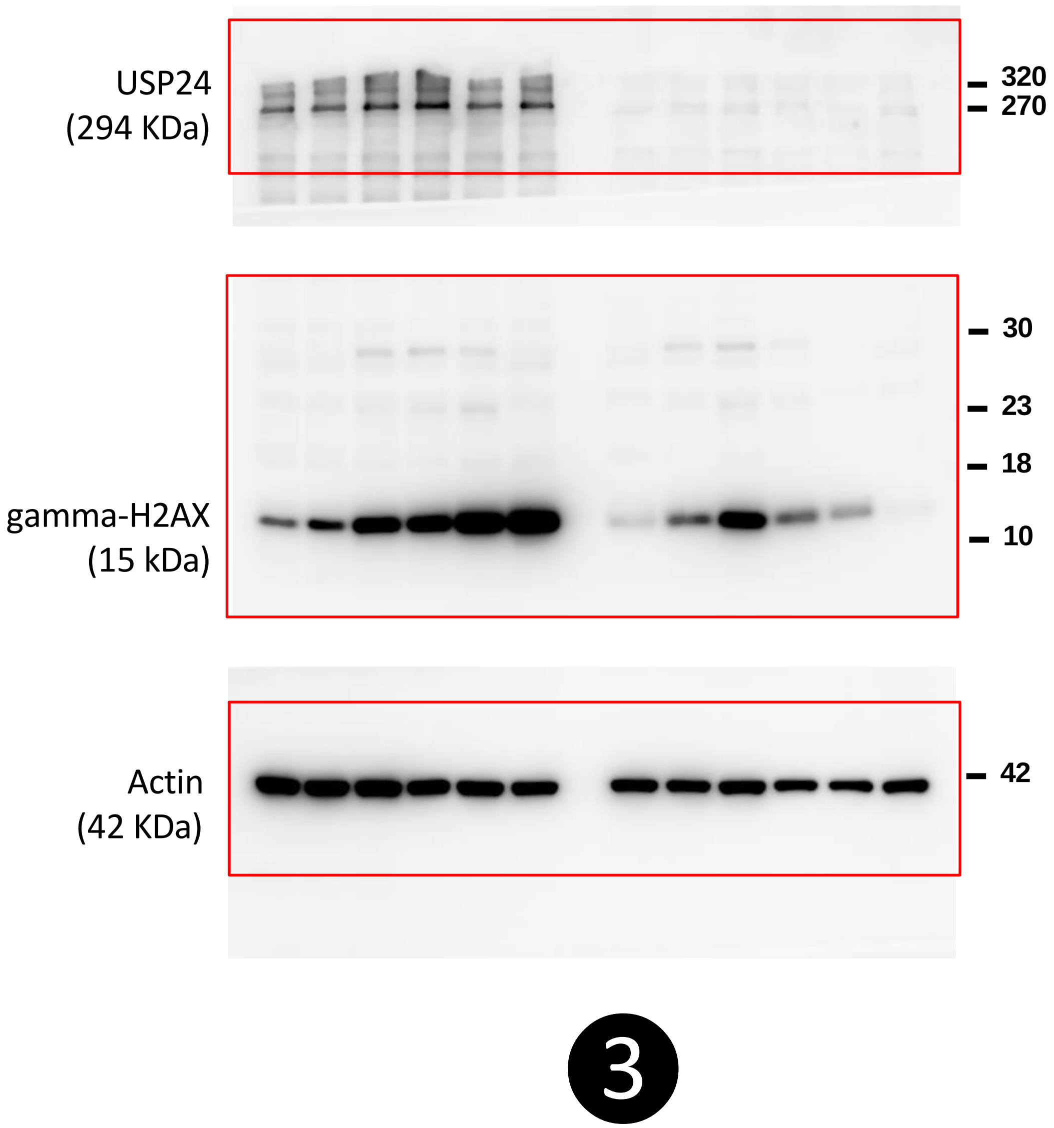
USP24
(294 KDa)
320
270
gamma-H2AX
(15 kDa)
30
23
18
10
Actin
(42 KDa)
42
3

Fig6.A(a)

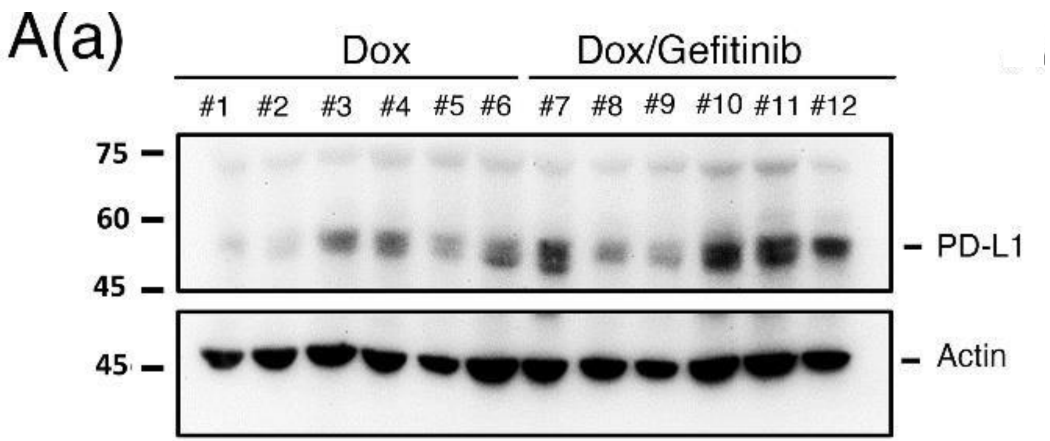

PD-L1

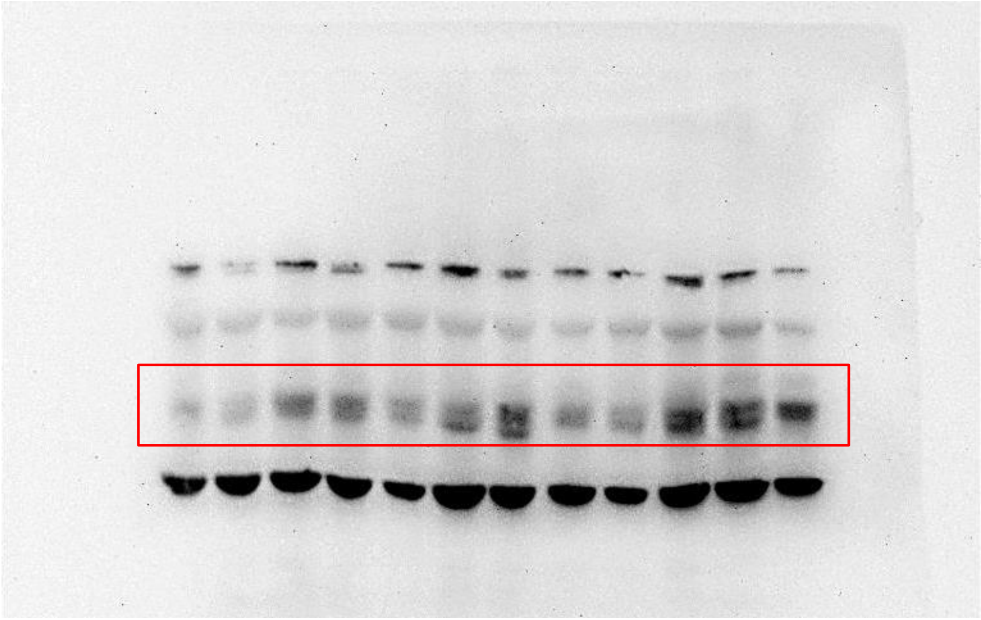

Actin

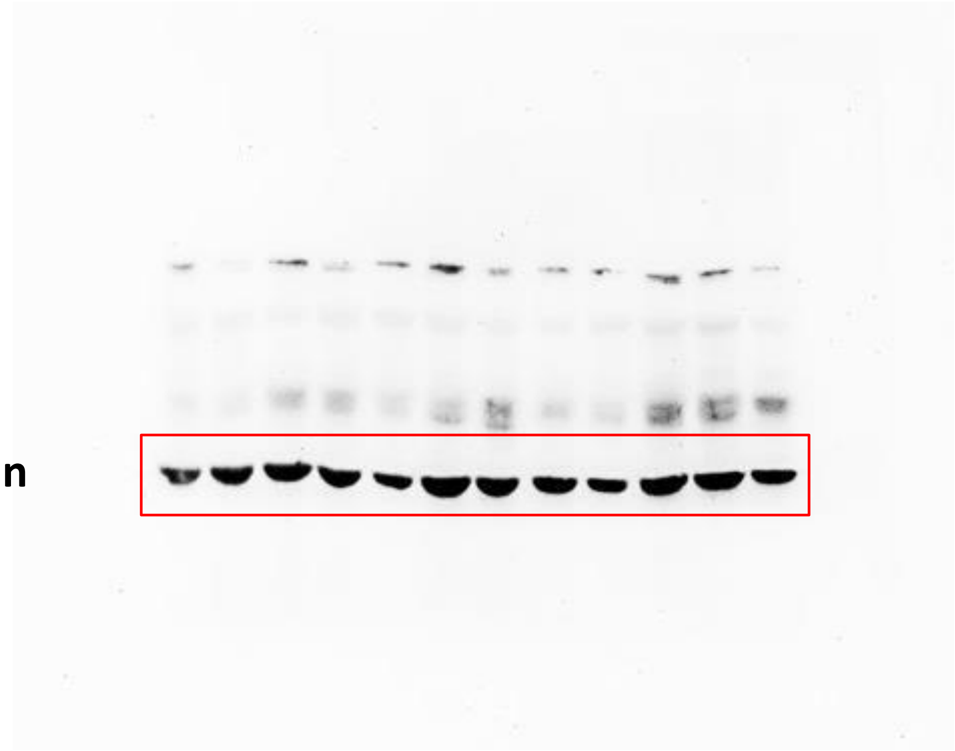

Fig6.B(a)

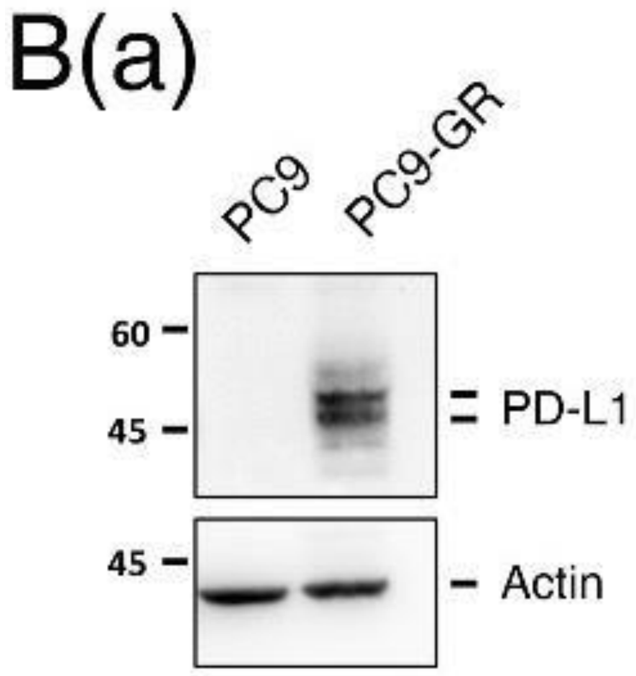

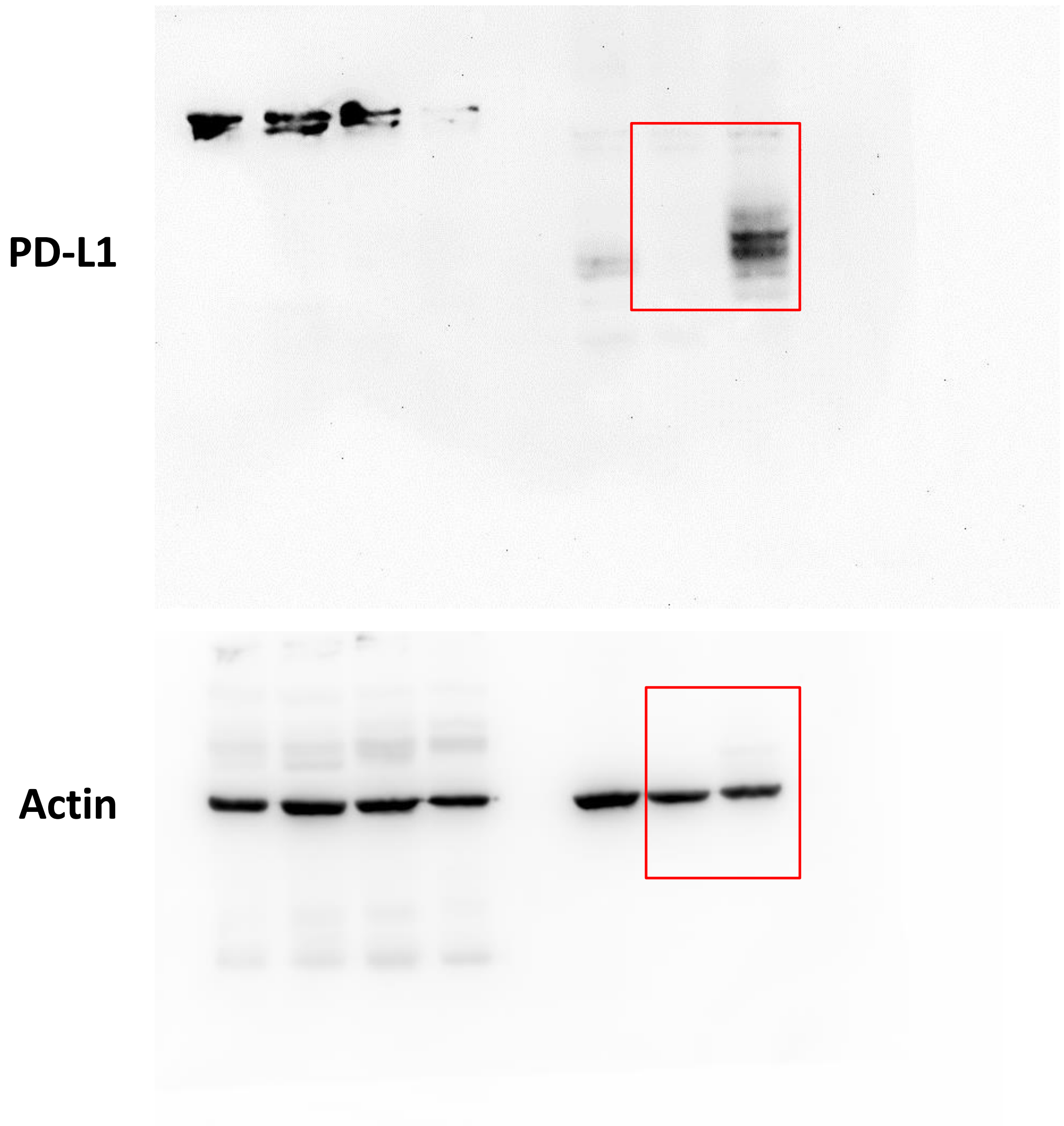

Fig6.C

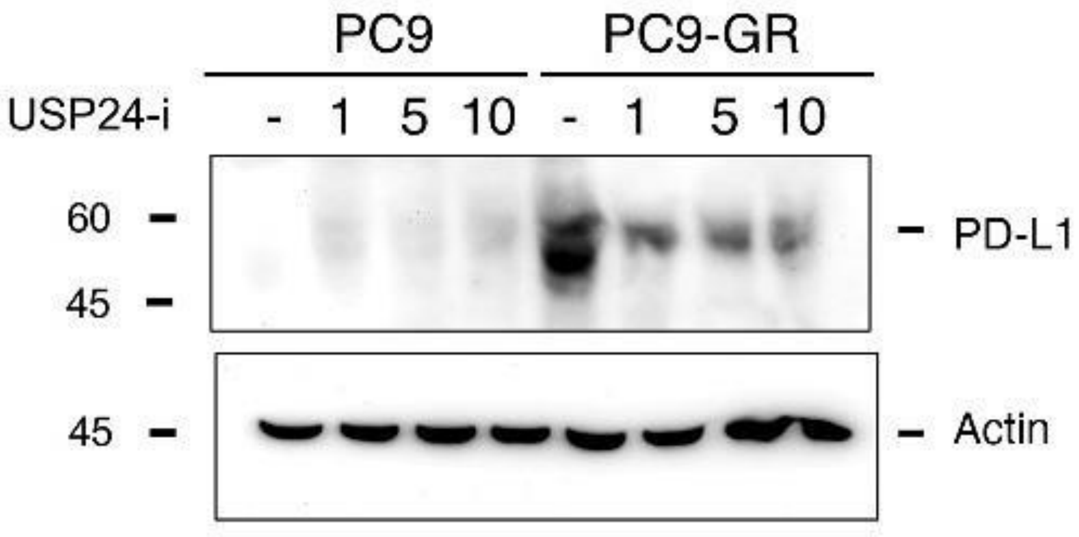

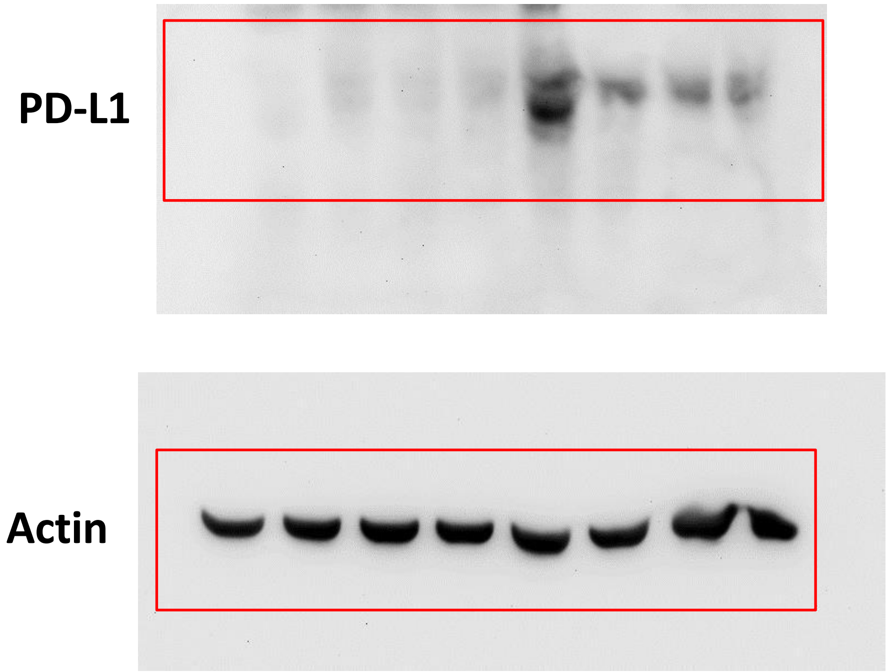

Fig6.D

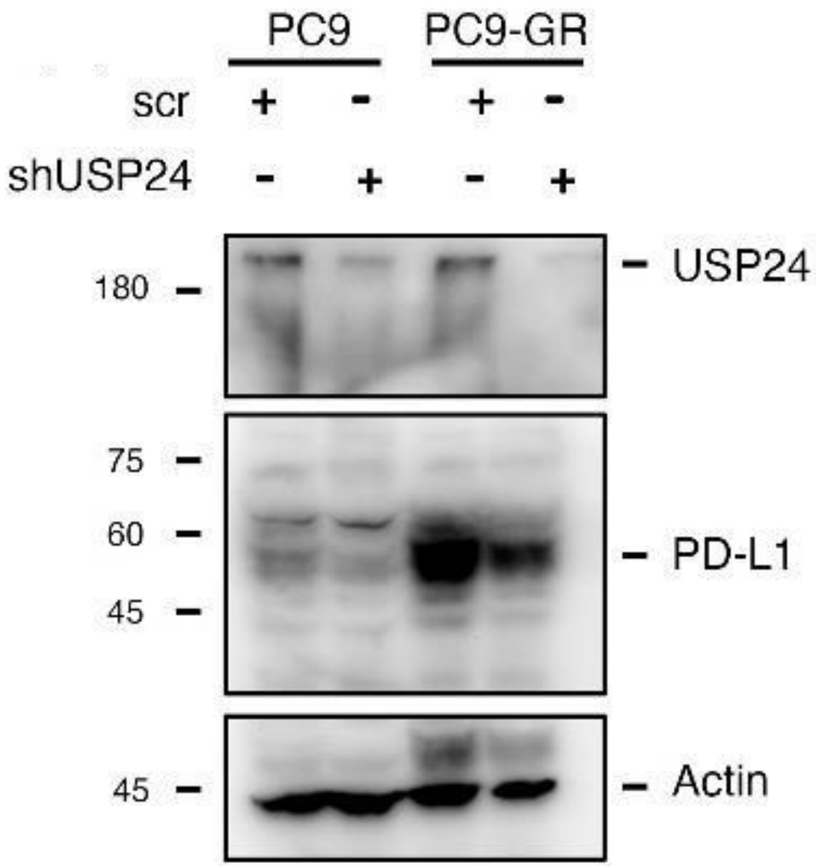
PC9
PC9-GR
scr + - + -
shUSP24 - + - +
180
75
60
45
45
USP24
PD-L1
Actin

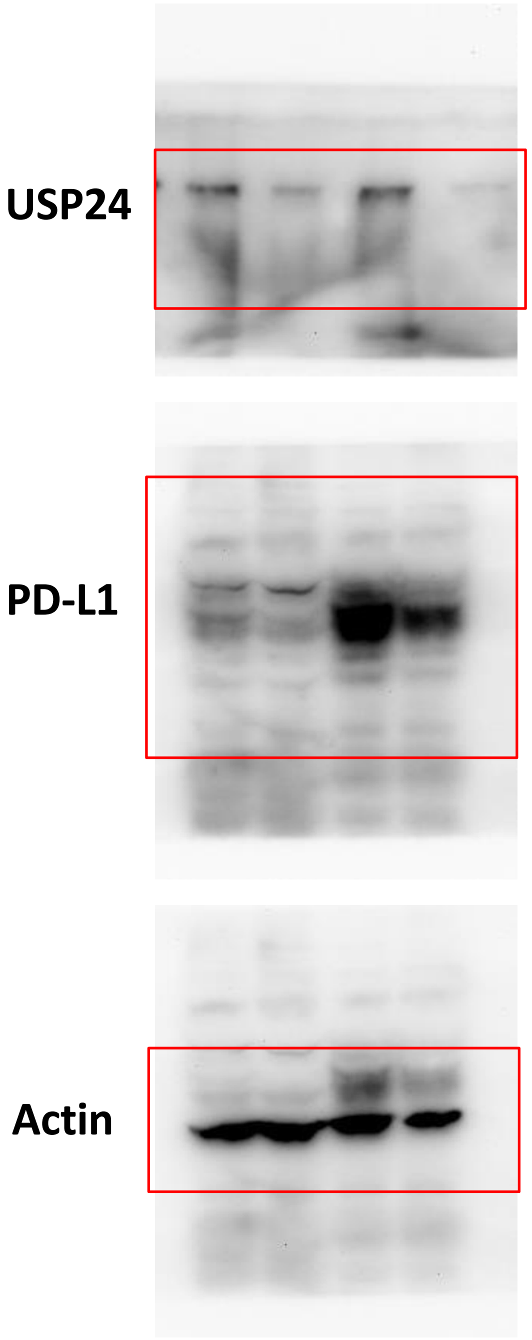
USP24
PD-L1
Actin

Fig6.E

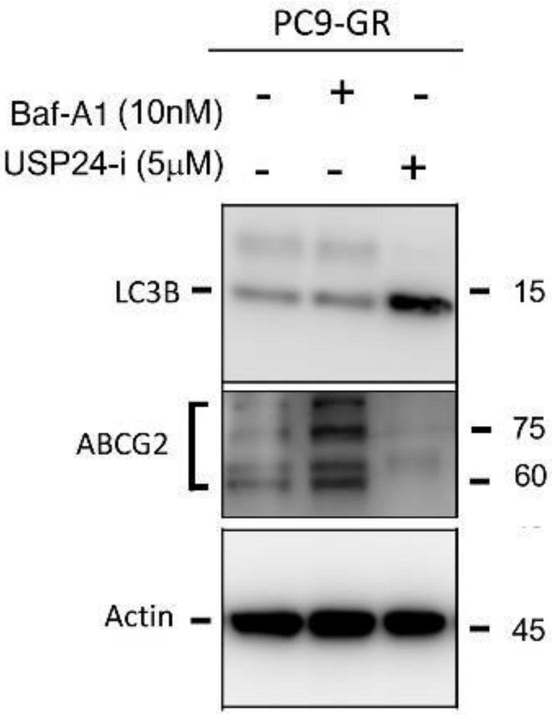
PC9-GR
Baf-A1 (10nM) - + -
USP24-i (5μM) - - +
LC3B
ABCG2
Actin
15
75
60
45

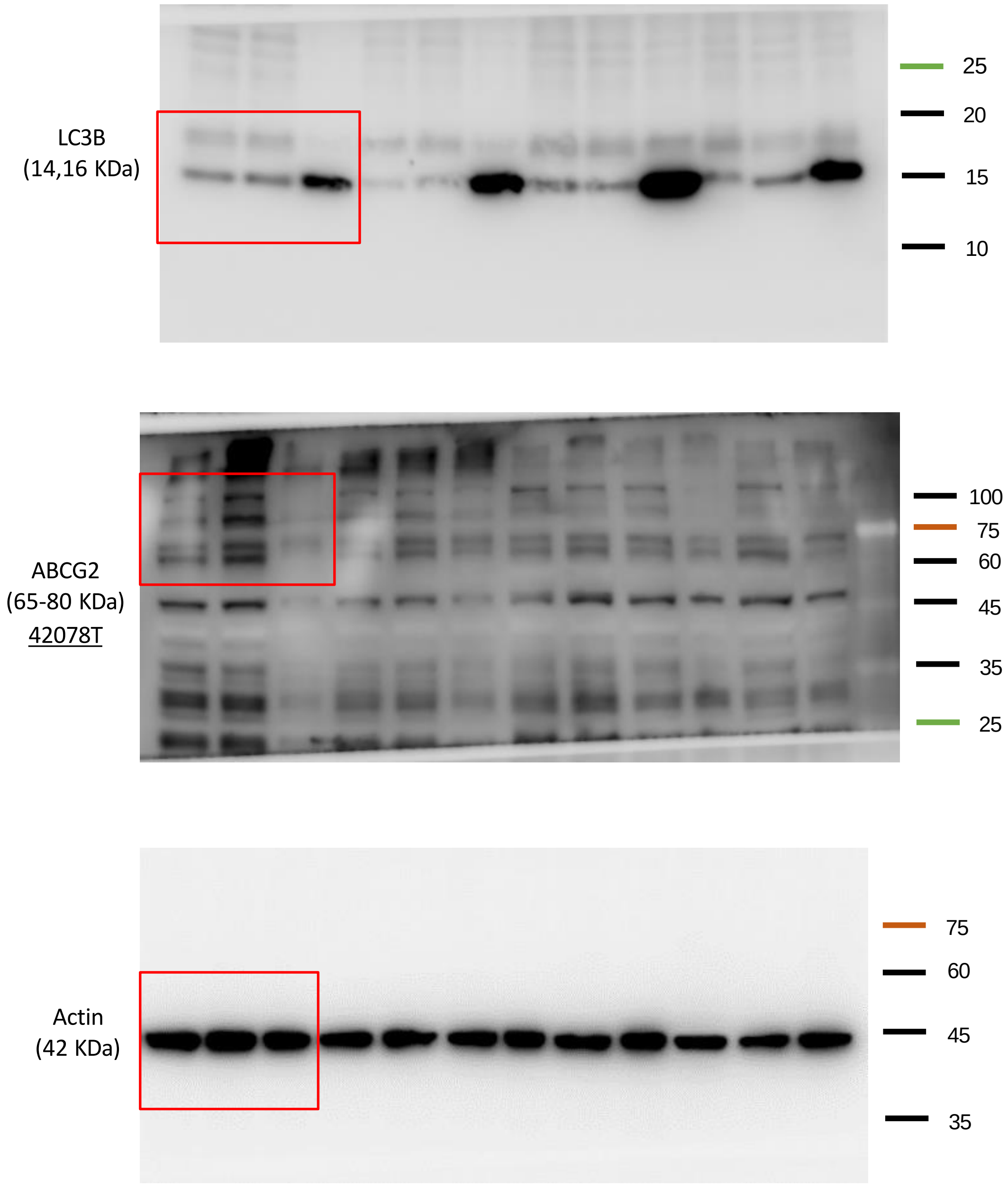
LC3B
(14,16 KDa)
25
20
15
10
ABCG2
(65-80 KDa)
42078T
100
75
60
45
35
25
Actin
(42 KDa)
75
60
45
35

Fig6.G(a)

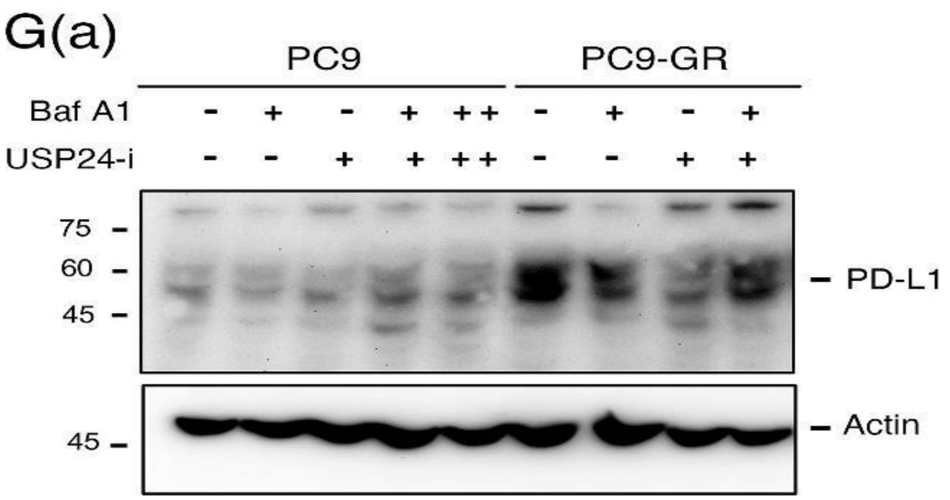

PD-L1

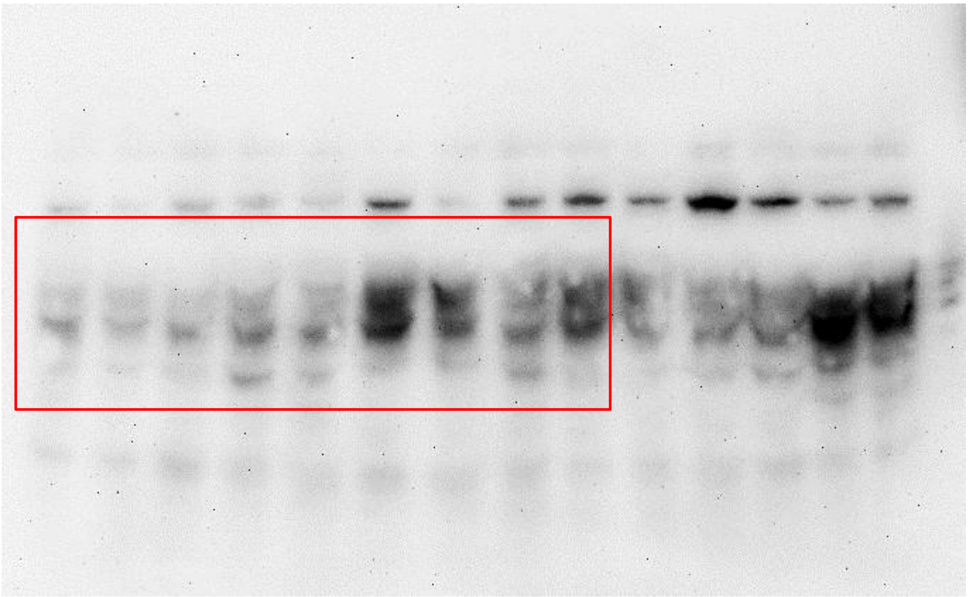

Actin

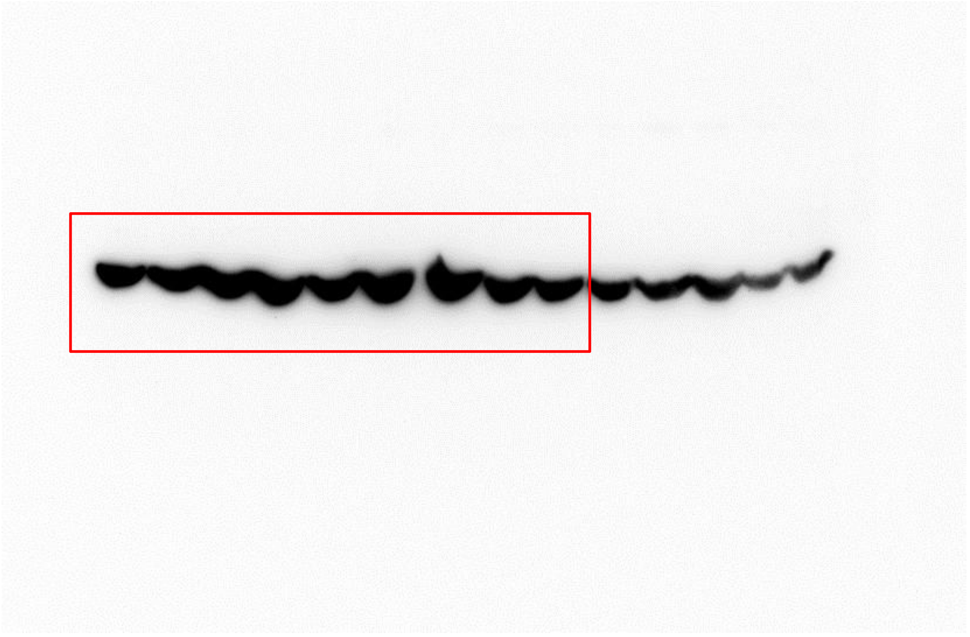

Fig6.H(a)

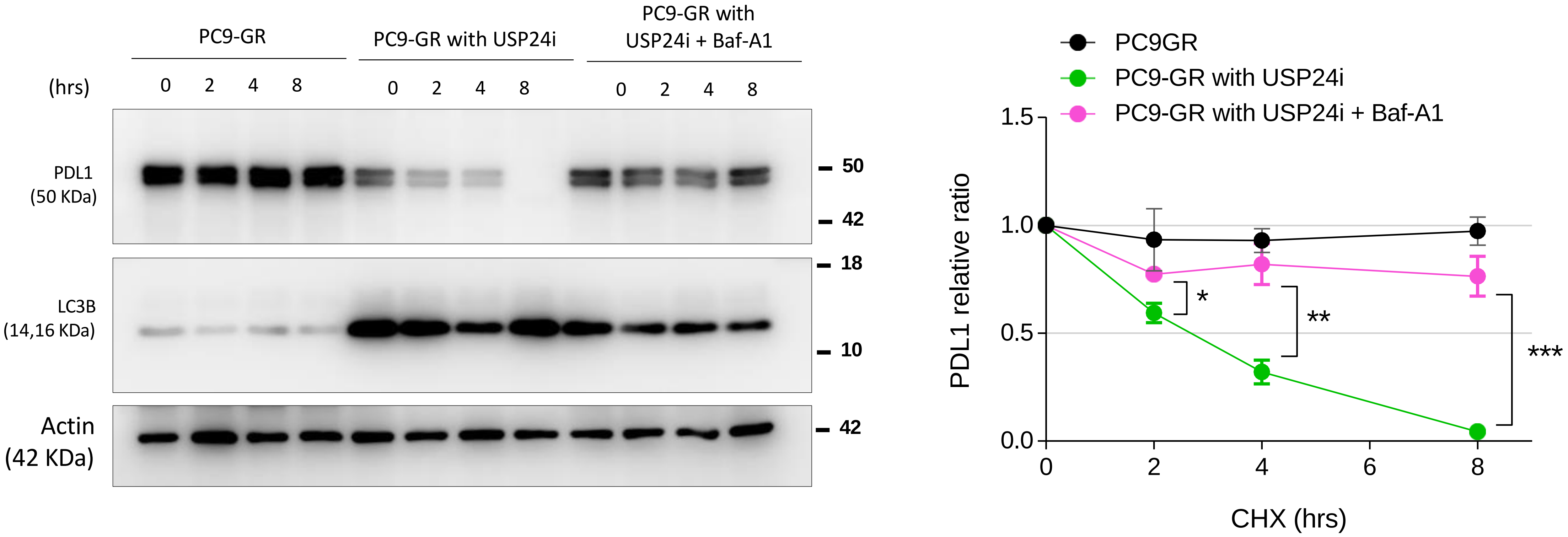
PC9-GR
PC9-GR with USP24i
PC9-GR with USP24i + Baf-A1
(hrs)
0 2 4 8
0 2 4 8
0 2 4 8
PDL1 (50 KDa)
LC3B (14,16 KDa)
Actin (42 KDa)
50
42
18
10
42
PC9GR
PC9-GR with USP24i
PC9-GR with USP24i + Baf-A1
PDL1 relative ratio
1.5
1.0
0.5
0.0
0
2
4
6
8
*
**
***
CHX (hrs)

Fig6.H(a)

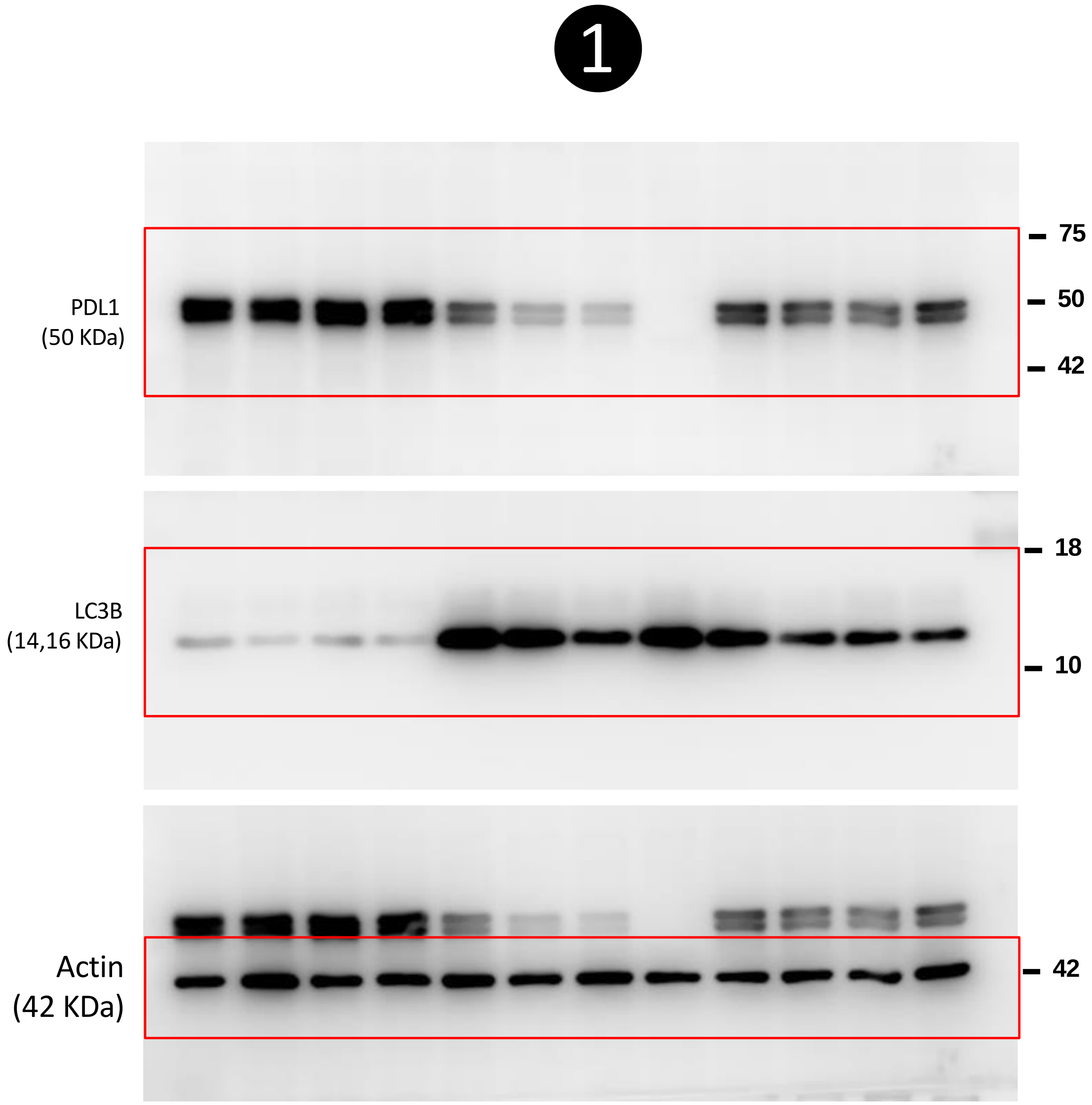

Fig6.H(a)

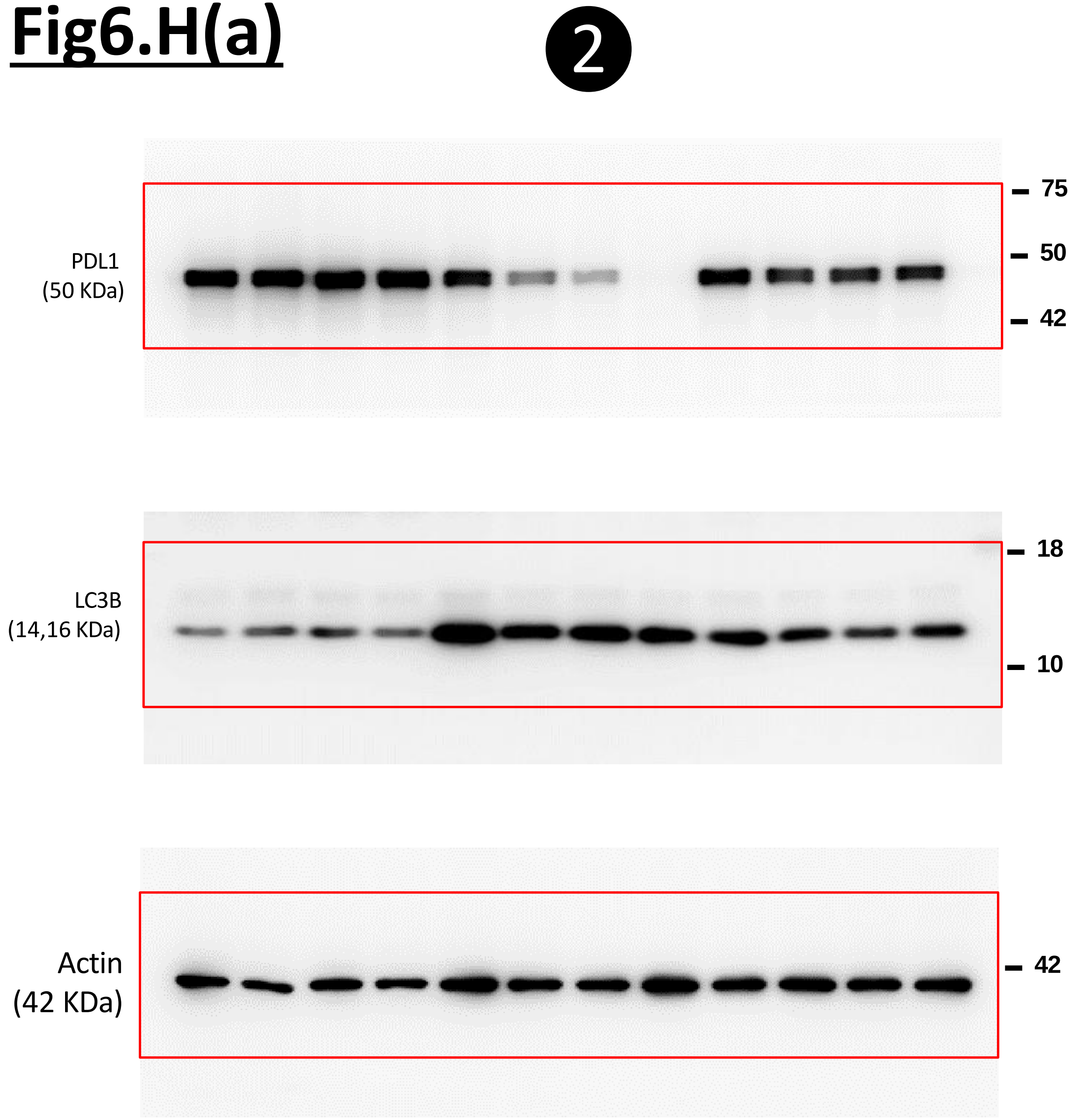

2
PDL1
(50 KDa)
75
50
42
LC3B
(14,16 KDa)
18
10
Actin
(42 KDa)
42

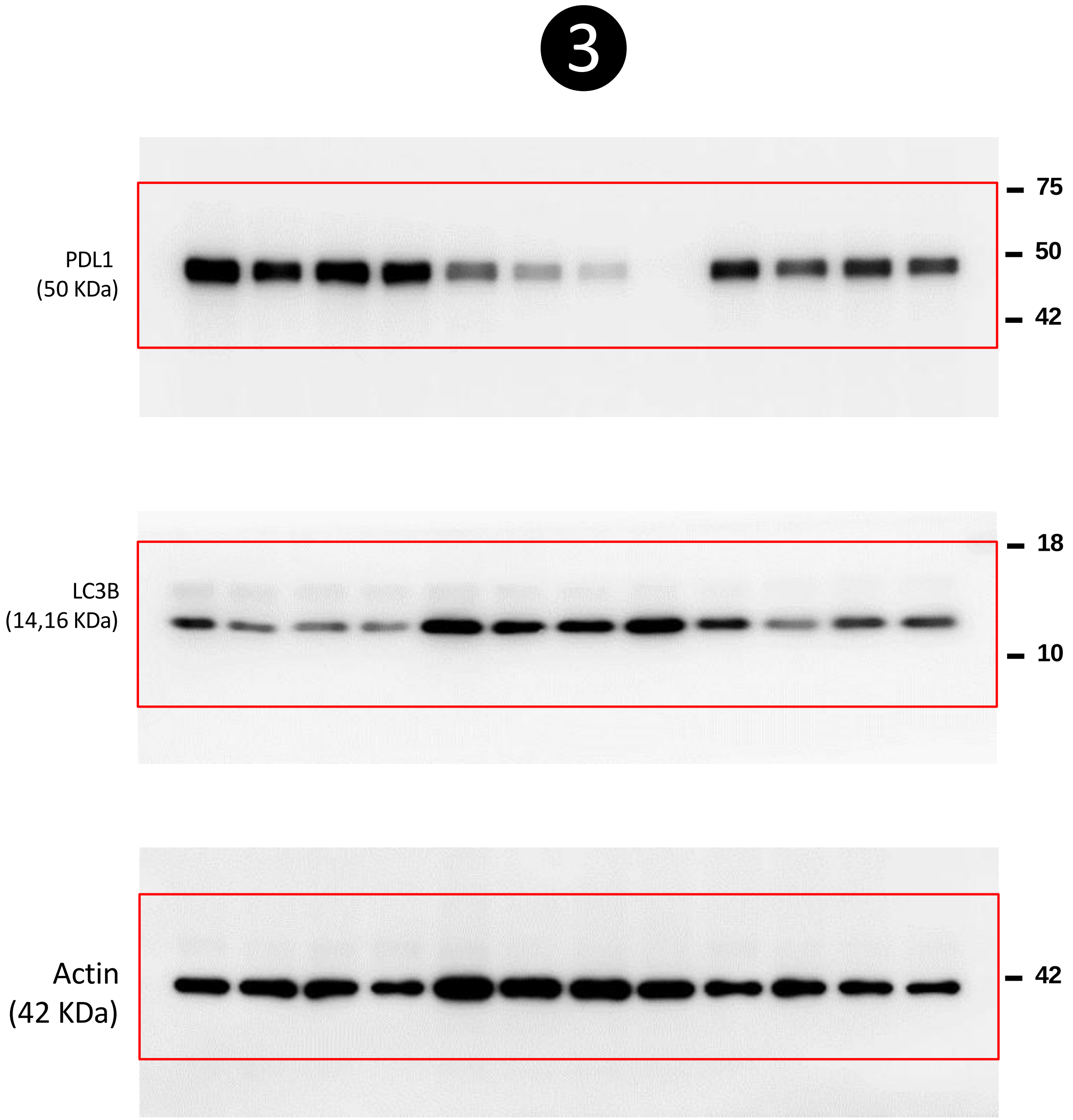

3
PDL1
(50 KDa)
75
50
42
LC3B
(14,16 KDa)
18
10
Actin
(42 KDa)
42

Fig6.I(a)

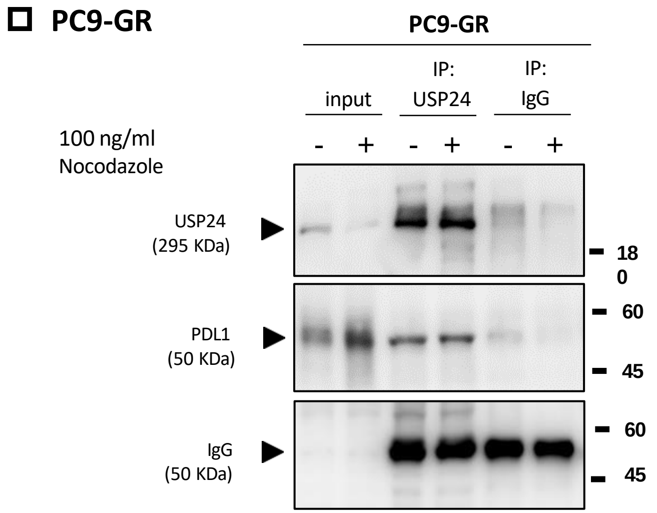

PC9-GR
PC9-GR
input
IP: USP24
IP: IgG
100 ng/ml Nocodazole
- + - + - +
USP24 (295 KDa)
PDL1 (50 KDa)
IgG (50 KDa)
18
0
60
45
60
45

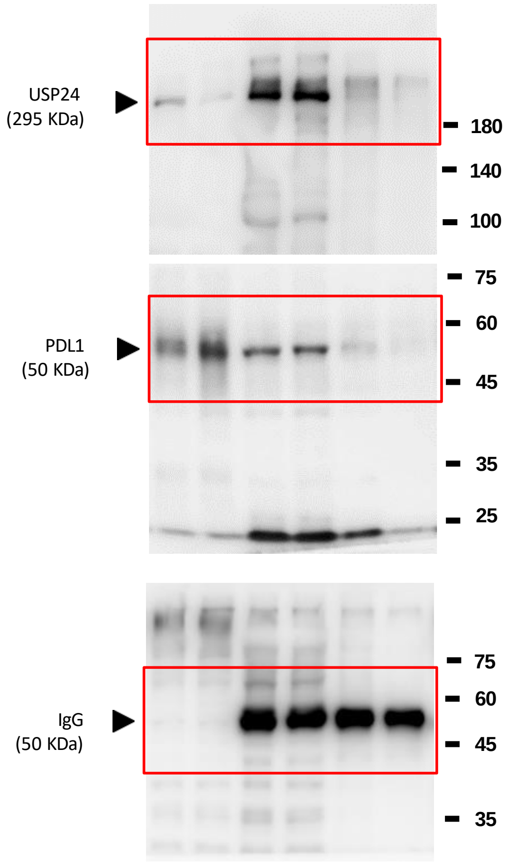

USP24 (295 KDa)
180
140
100
75
60
PDL1 (50 KDa)
45
35
25
75
60
IgG (50 KDa)
45
35

Fig6.I(b)

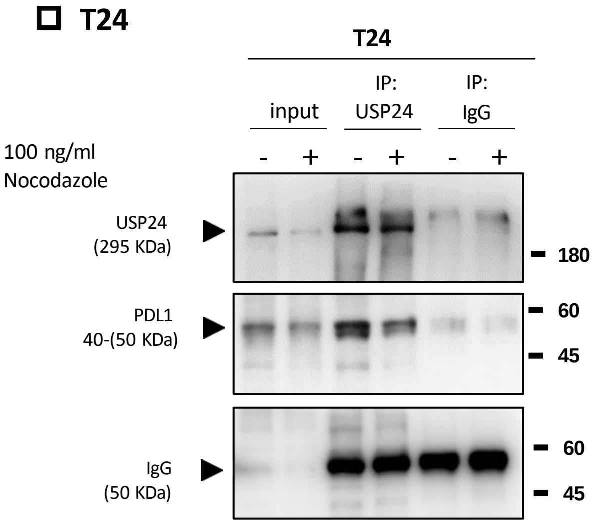

T24
T24
input
IP: USP24
IP: IgG
100 ng/ml Nocodazole
- + - + - +
USP24 (295 KDa)
180
PDL1 40-(50 KDa)
60
45
IgG (50 KDa)
60
45

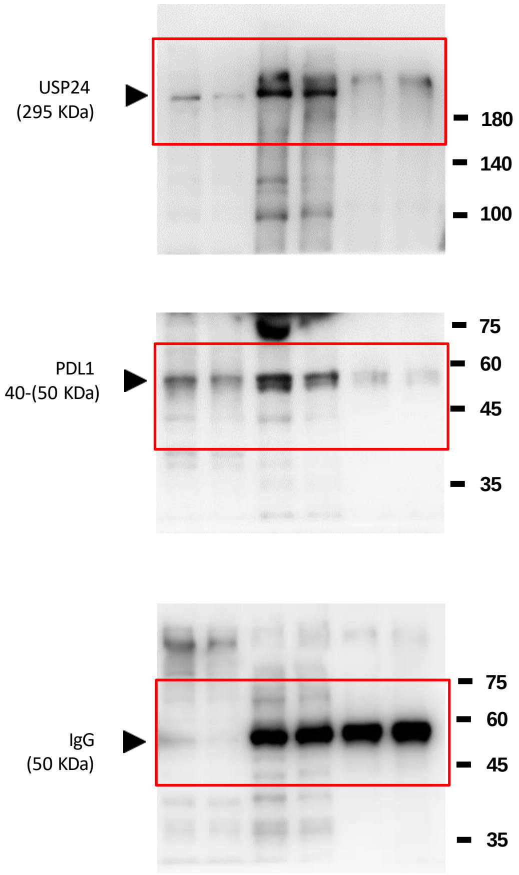

USP24 (295 KDa)
180
140
100
PDL1 40-(50 KDa)
75
60
45
35
IgG (50 KDa)
75
60
45
35

Fig6.J

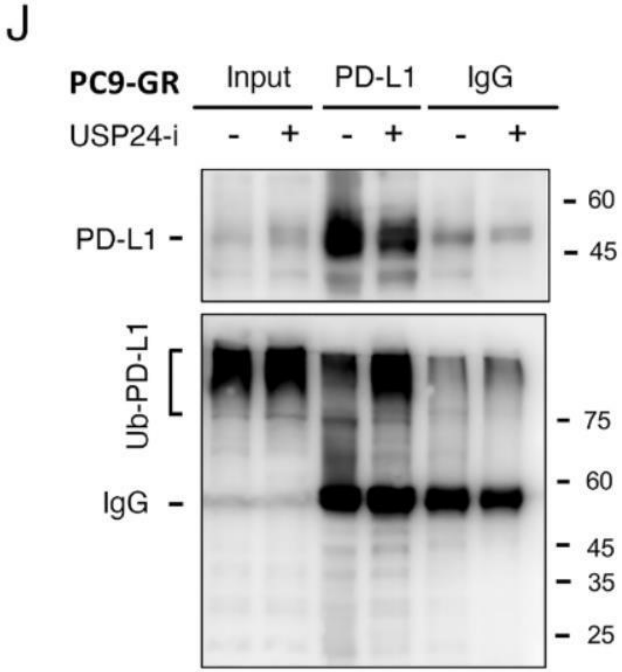
J
PC9-GR
Input
PD-L1
IgG
USP24-i
- + - + - +
PD-L1
60
45
Ub-PD-L1
IgG
75
60
45
35
25

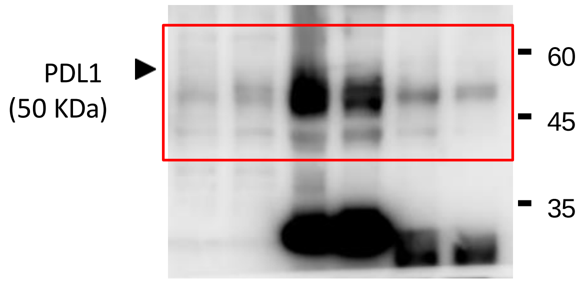
PDL1
(50 KDa)
60
45
35

Fig6.K

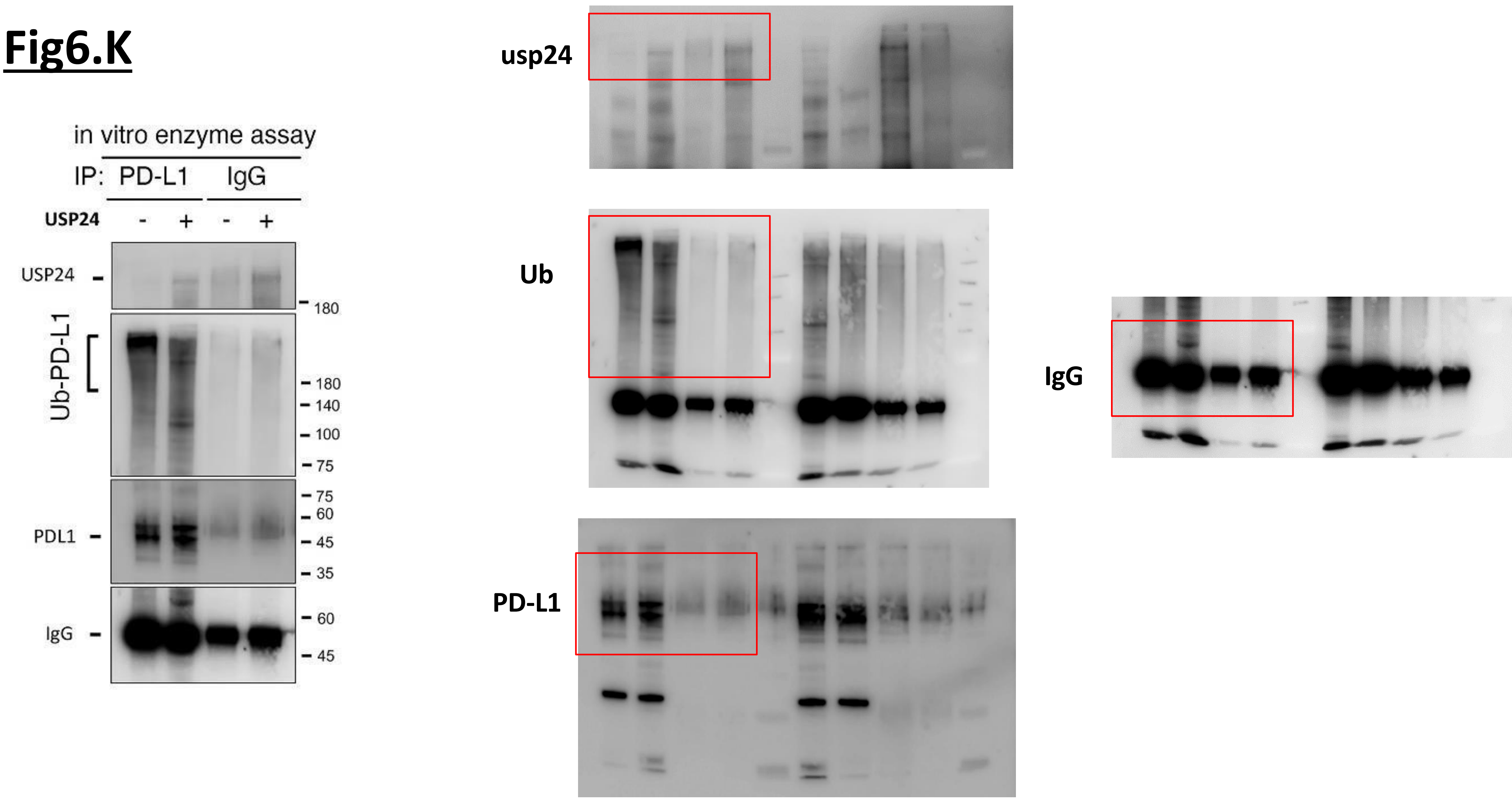
in vitro enzyme assay
IP: PD-L1 IgG
USP24 - + - +
USP24
Ub-PD-L1
PDL1
IgG
180
180
140
100
75
75
60
45
35
60
45
usp24
Ub
PD-L1
IgG

Fig.8.B(a)

USP24WT
#1 : 1222
#2 : 1223
#3 : 1227
#4 : 1249
#5 : 1357
#6 : 1363

USP24KO
#7 : 141
#8 : 142
#9 : 152
#10 : 158
#11 : 159
#12 : 160

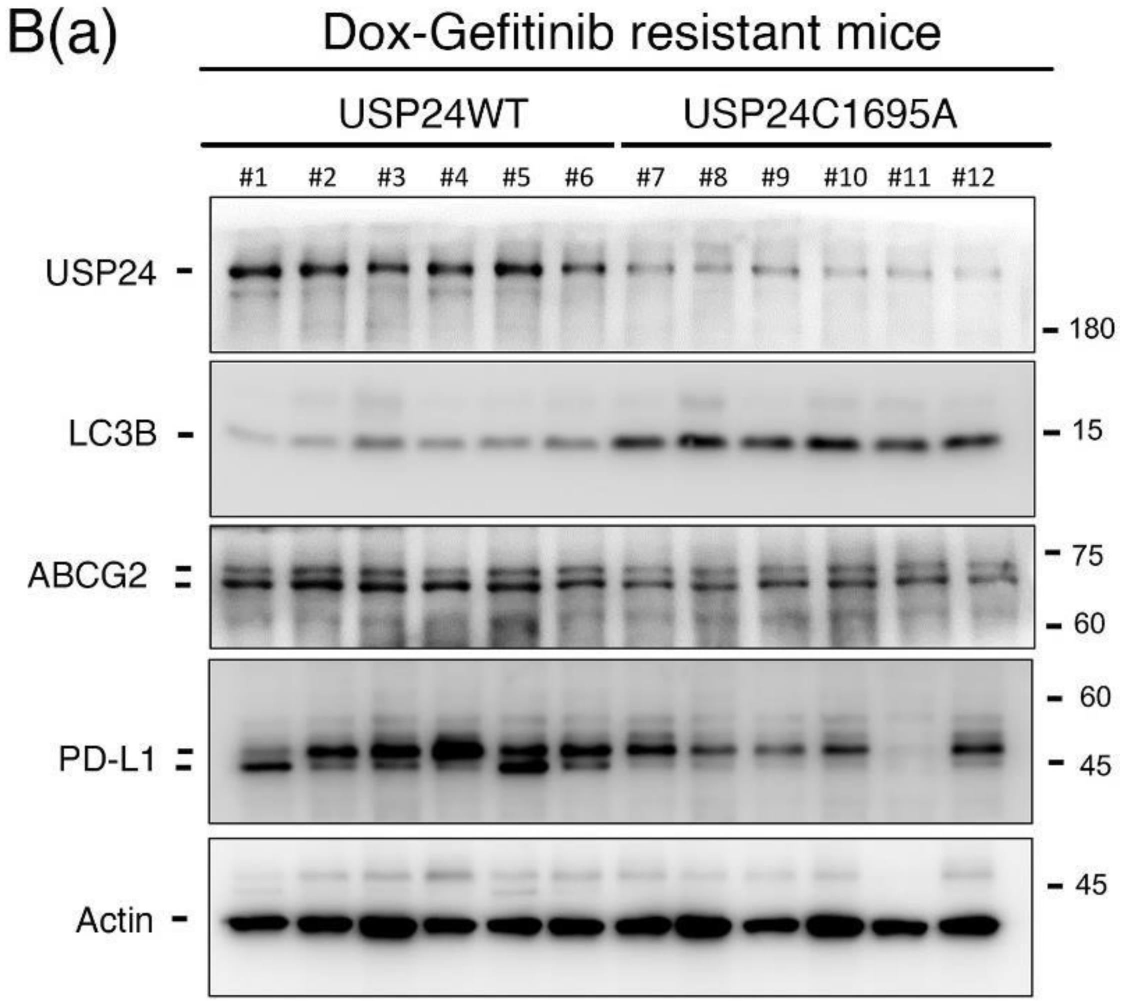

Fig.8.B(a)

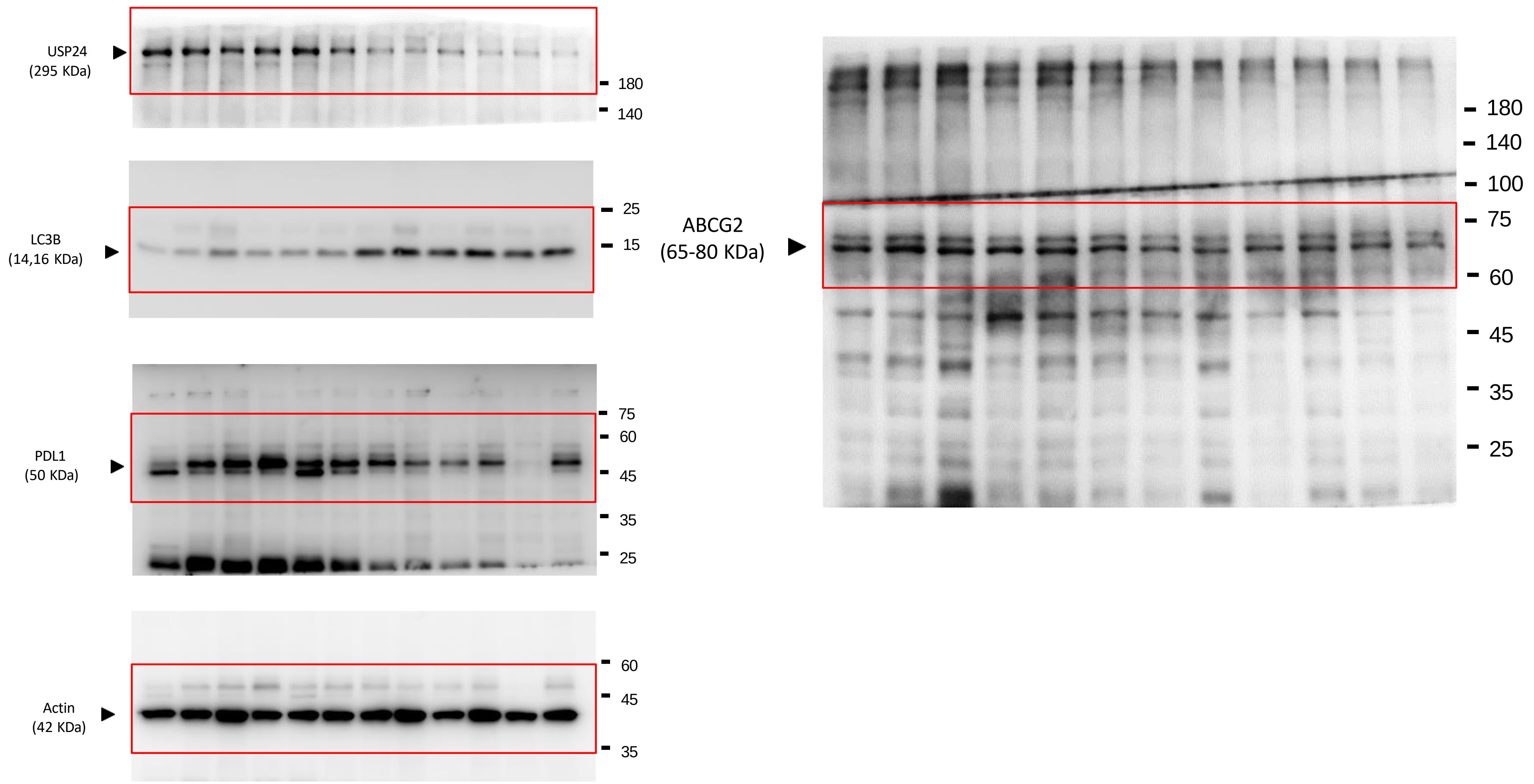
USP24
(295 KDa)
180
140
LC3B
(14,16 KDa)
25
15
PDL1
(50 KDa)
75
60
45
35
25
Actin
(42 KDa)
60
45
35
ABCG2
(65-80 KDa)
180
140
100
75
60
45
35
25

Suppl.Fig.2.E

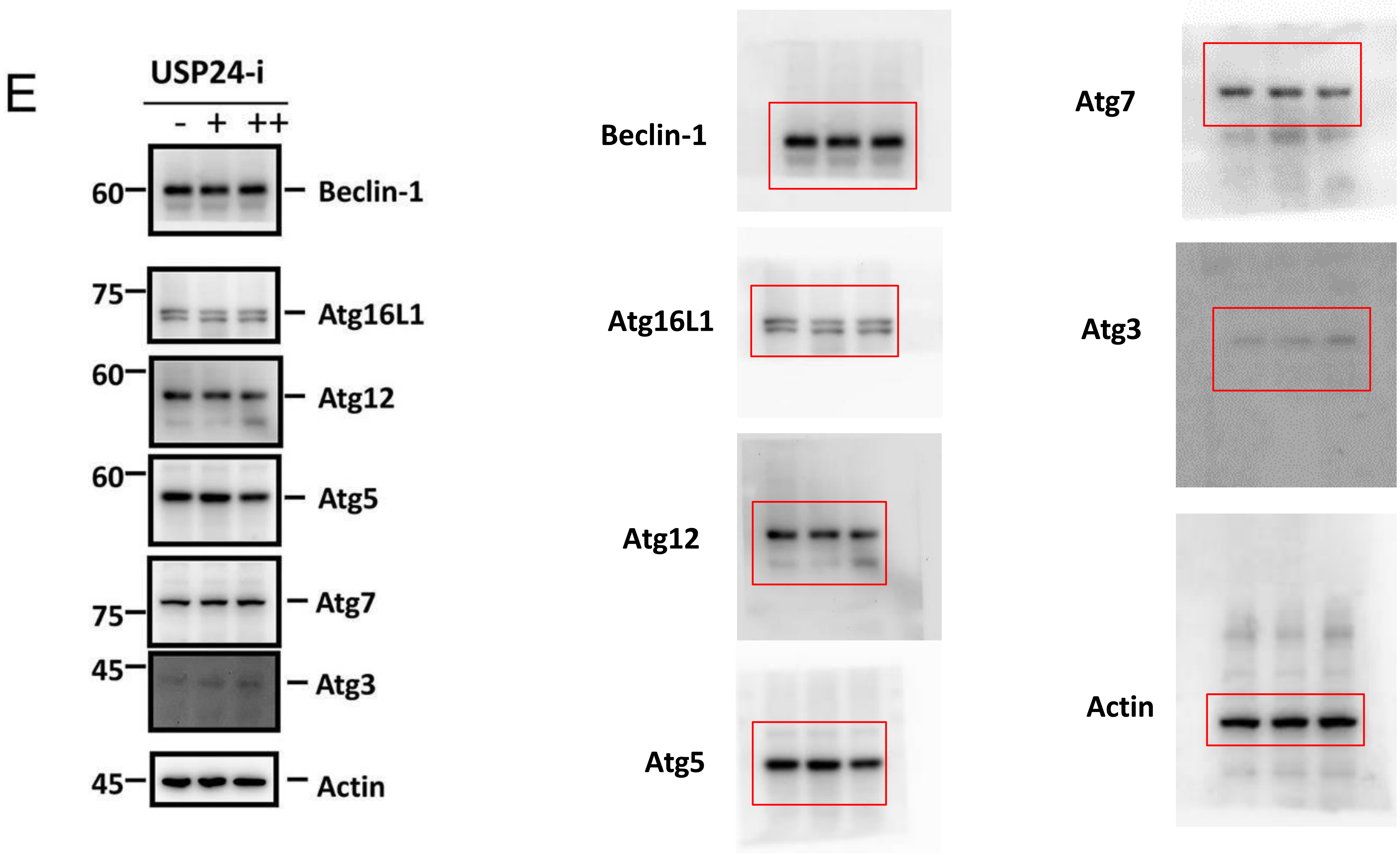
E
USP24-i
- + ++
60
Beclin-1
75
Atg16L1
60
Atg12
60
Atg5
75
Atg7
45
Atg3
45
Actin
Beclin-1
Atg16L1
Atg12
Atg5
Atg7
Atg3
Actin

Suppl.Fig.2.F

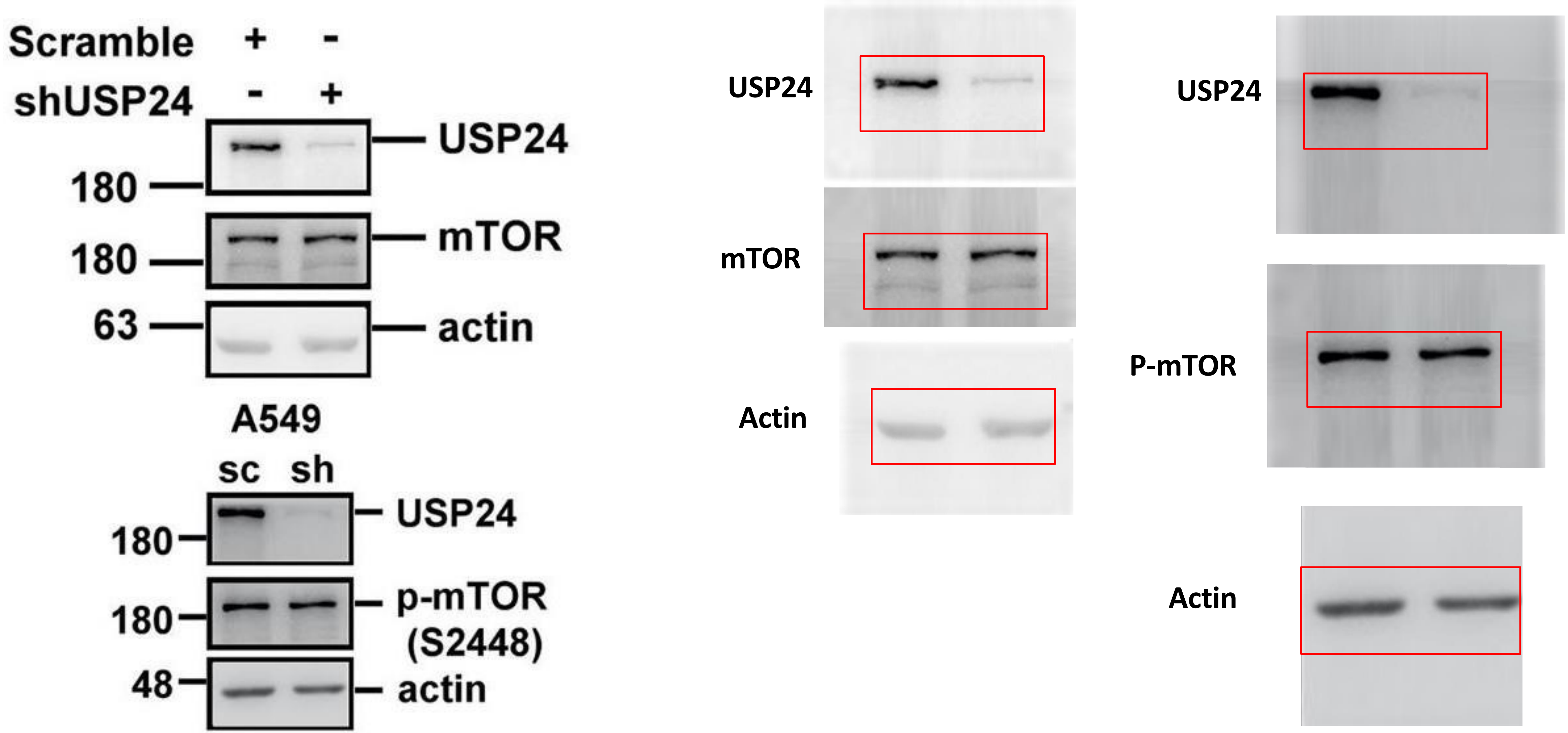
Scramble + -
shUSP24 - +
USP24
180
180
mTOR
63
actin
A549
sc sh
USP24
180
180
p-mTOR
(S2448)
48
actin
USP24
mTOR
Actin
USP24
P-mTOR
Actin
